# Light-Driven Membrane Assembly, Shape-Shifting, and Tissue Formation in Chemically Responsive Synthetic Cells

**DOI:** 10.1021/jacs.3c09894

**Published:** 2023-11-14

**Authors:** Youngjun Lee, Alessandro Fracassi, Neal K. Devaraj

**Affiliations:** Department of Chemistry and Biochemistry, University of California, San Diego, 9500 Gilman Drive, La Jolla, California 92093, United States

## Abstract

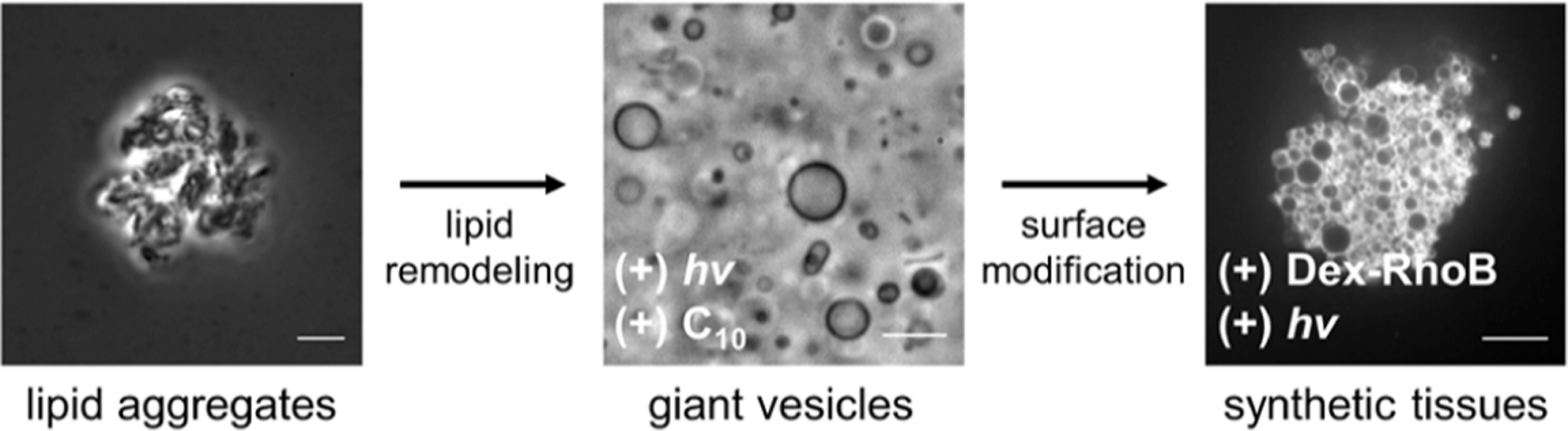

Living systems create remarkable complexity from a limited repertoire of biological building blocks by controlling assembly dynamics at the molecular, cellular, and multicellular level. An open question is whether simplified synthetic cells can gain similar complex functionality by being driven away from equilibrium. Here, we describe a dynamic synthetic cell system assembled using artificial lipids that are responsive to both light and chemical stimuli. Irradiation of disordered aggregates of lipids leads to the spontaneous emergence of giant cell-like vesicles, which revert to aggregates when illumination is turned off. Under irradiation, the synthetic cell membranes can interact with chemical building blocks, remodeling their composition and forming new structures that prevent the membranes from undergoing retrograde aggregation processes. The remodeled light-responsive synthetic cells reversibly alter their shape under irradiation, transitioning from spheres to rodlike shapes, mimicking energy-dependent functions normally restricted to living materials. In the presence of noncovalently interacting multivalent polymers, light-driven shape changes can be used to trigger vesicle cross-linking, leading to the formation of functional synthetic tissues. By controlling light and chemical inputs, the stepwise, one-pot transformation of lipid aggregates to multivesicular synthetic tissues is feasible. Our results suggest a rationale for why even early protocells may have required and evolved simple mechanisms to harness environmental energy sources to coordinate hierarchical assembly processes.

## Introduction

Self-assembly of synthetic building blocks has often been used to emulate complex biological structures and functions.^1,2^ Typically, synthetic self-assembly is thermodynamically driven and governed by the intrinsic interactions between chemical building blocks to produce stable ensembles.^3,4^ However, from a biological context, thermodynamically favored assemblies triggered by misregulated molecules can lead to the formation of nonfunctional aggregates or, even worse, assemblies that disrupt normal biological processes.^5^ Living systems are therefore constantly using external energy to drive assemblies away from equilibrium and control their thermodynamic states, which can lead to the creation of remarkably complex and functional structures, whose existence and properties are maintained by energy consumption. The recent development of chemical- or light-fueled dissipative systems,^6–11^ oscillatory chemical networks,^12–14^ and dynamic natural–synthetic hybrid complexes^15,16^ has begun to address the discrepancy between synthetic and biological assembly. Despite these advances, our ability to create abiological assemblies that show more complex functions, such as collective behavior in response to external stimuli or hierarchical transformations across multiple length scales, still significantly lags behind that of living organisms.

Here, we show that integrating light-responsive lipid building blocks with chemical processes allows artificial cells to manifest emergent assembly properties reminiscent of those observed in living cells. Photoswitchable lipids enable the de novo light-driven generation of dynamic lamellar vesicles from disordered structures. The metastable vesicles can be transformed in a stepwise manner by interacting with chemical building blocks through dynamic covalent chemistry and noncovalent multivalent interactions.^17,18^ Overall, we demonstrate that light combined with chemical building blocks enables the stepwise transformation of simple lipid aggregates toward more ordered assemblies with increasing hierarchical complexity, encompassing protocell membrane formation, membrane shape-shifting, and, ultimately, the development of tissue-like synthetic cell networks.

**Figure 1:**
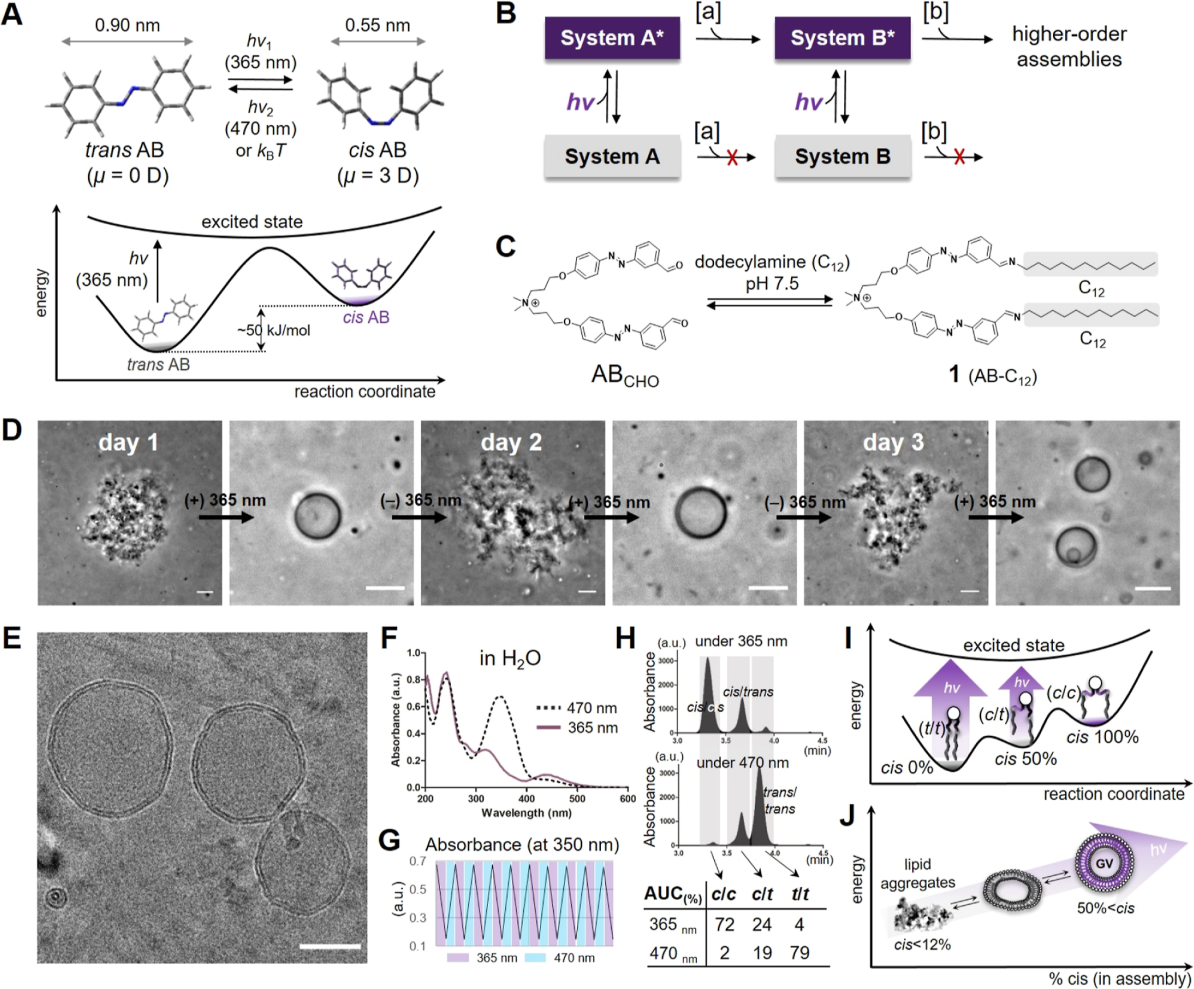
Light-driven lipid vesicle assembly using azobenzene (AB) photoswitches. (A) Top: chemical structures and reported molecular properties of the trans- and cis-AB isomers. *h*, Planck constant; *v*, frequency; *k*_B_, Boltzmann constant; *T*, temperature. Bottom: scheme depicting the relative energy states of AB isomers. (B) Flow diagram depicting a series of system transformations through interactions with light (*hv*) and external stimuli (a,b). Purple, light-driven states. Gray, equilibrium states. Note that higher-order assemblies are accessible through light-driven interactions. (C) Imine formation reaction between the aldehyde precursor (**AB**_**CHO**_) and dodecylamine to form lipid product **1** (**AB-C**_**12**_) in pH 7.5 buffer solution. (D) Time-series micrographs of in situ **1** products under the irradiation of 365 nm LED light followed by overnight exposure to ambient conditions for three alternating cycles. Measured ambient conditions: temperature, 19–21 °C; room light, 32 W-4100 K-fluorescent bulbs, 250–350 lux. Scale bars: 10 μm. (E) Representative cryogenic electron microscopy (cryo-EM) image of vesicles of **1**. Scale bars: 50 nm. (F) Absorption spectra of **1** under the 365 (solid line) or 470 nm (dashed line) LED light in H_2_O. [**1**] = 20 μM. (G) Absorbance changes of **1** at 350 nm as a function of ten alternating cycles of light irradiation (for 1 min) using 365 and 470 nm LED light in H_2_O. [**1**] = 20 μM. (H) Chromatograms using a liquid chromatography-diode array detector (LC-DAD) method at 250 nm for in situ **1** products after being exposed to 365 (top) or 470 nm (middle) light. Bottom: table of the relative isomeric ratios of **1** calculated from the chromatograms. c/c, cis/cis; c/t, cis/trans; and t/t, trans/trans. (I,J) Scheme depicting light-dependent changes in the relative energy states for monomeric **1** (I) and the assemblies of **1** (J).

The use of light as an energy source offers significant benefits such as precise spatiotemporal control and lack of waste products.^19^ As light-driven actuators, azobenzene (AB) functional groups have been well-studied.^20,21^ Briefly, the thermodynamically stable trans-AB isomer can absorb 365 nm light, forming a higher energy cis-isomer (Δ ∼ 50 kJ/mol, trans → cis).^22^ In the absence of 365 nm light, spontaneous cis-to-trans thermal isomerization occurs over time, ranging from milliseconds to days.^23^ This conversion (cis → trans) can also be rapidly triggered by exposure to a longer wavelength of light (470 nm). By applying a 365 nm light stimulus, it has been shown that photosensitive AB-based amphiphiles can exhibit transient physicochemical properties, owing to the distinct molecular geometry, length, and dipole moment of cis isomers, which are different from the trans (Figure 1A).^21^ Several studies have explored light-responsive AB supramolecular assemblies, such as photosensitive lipid vesicles.^24–26^ However, in these studies, light is typically coupled to the disruption of assemblies, for instance, by creating highly soluble monomers or inhibiting intermolecular interactions, ultimately creating less ordered structures. This stands in contrast to how light is utilized by living matter to drive chemical transformations for maintaining order while simultaneously avoiding thermodynamic traps. As a result, harnessing light energy as a driving force to generate and maintain dynamic structures that can undergo further transformations has remained an elusive goal.

## Results and Discussion

### Light-Driven Formation of Giant Vesicle Assemblies

While single-chain AB containing amphiphiles have been well-studied,^27–29^ lipids containing two AB chains are much less explored. We synthesized a series of AB amphiphiles containing two hydrophobic tails and found that a monomer composed of two symmetrical AB tails with a quaternary ammonium headgroup can spontaneously self-assemble into cell-sized giant vesicles (1–20 μm) in saline solution (Figure S1). Unsurprisingly, under 365 nm light, these assemblies were disrupted (Figure S1), similar to previous reports.^30–32^ Several studies have shown that various AB amphiphiles can be embedded into phospholipid vesicles, creating membranes that are ruptured by UV light.^25,26,30^ To make more lipid-like molecules, we introduced hydrophobic alkyl groups through in situ imine formation on the AB building blocks. In addition, by employing reversible covalent chemistry, we also sought to explore the potential of light to shift chemical equilibrium and facilitate membrane assemblies to interact with alternative chemical building blocks (Figure 1B).

The reaction between AB-aldehyde (**AB**_**CHO**_) and dodecylamine (C_12_) was carried out in HEPES-buffered saline at pH 7.5 overnight (Figure 1C), and formation of imine **1** (**AB-C**_**12**_) was confirmed by characterization of the reductive amination product (Figure S2).^33,34^ When generated in situ, **1** formed nonuniform insoluble aggregates without producing any visible lamellar structures or vesicles (Figure 1D). However, in the presence of 365 nm LED light, aggregates of **1** underwent a spontaneous transformation from irregular structures to giant vesicles within 30 min (Movie S1 and Figure S3), resulting in clearer and more vividly colored reaction solutions. Aggregate assemblies appeared to directly transform to lamellar membranes, suggesting that soluble monomers are not a necessary intermediate state. Vesicle transformation coincided with an increase in the cis-**1** composition (Figure S3). The bilayer membrane structure of the vesicles was confirmed by cryogenic electron microscopy (cryo-EM) analysis (Figure 1E), and dynamic light scattering (DLS) analysis additionally confirmed the submicrometer-size assemblies before and after 365 nm light irradiation (Figure S4). The membrane thickness was estimated to be 3.31 ± 0.20 nm (Figure S4E). If light-activated vesicles were left in ambient conditions without a 365 nm light source, they reverted to aggregates over 4 h (Figure S3). The reversibility of the assembly transformation was demonstrated by three alternating cycles of light-induced aggregate-to-vesicle transformation (Figure 1D).

To determine the light-responsive properties of **1** in aqueous media, UV–vis spectroscopy was performed on isolated **1** under 365 and 470 nm LED light in water (Figure 1F,G). For ten cycles of light irradiation, **1** exhibited robust photoreversibility without any detectable photodegradation (Figure 1G). The isomers of **1** and their relative abundance were characterized by liquid chromatography (Figure 1H). We found three existing isomers, cis/cis (c/c), cis/trans (c/t), and trans/trans (t/t), as expected, given that **1** has two AB tails. Under the 365 nm irradiation, the c/c isomer was the dominant lipid species (72%), and the total proportion of cis-AB in **1** was 84% (Figure 1H, bottom and Table S1). In contrast, the t/t isomer was most abundant (79%) under 470 nm illumination, and 88% of the lipid tails consisted of trans-AB. Kinetic analysis of spontaneous cis → trans isomerization revealed that the half-life of the cis isomer is around 0.6 h under ambient room lighting (Figure S5 and Table S2). Four h after stopping the 365 nm irradiation, an equilibrium cis:trans lipid tail ratio of 12:88 was reached.

The emergence of giant vesicles after exposing aggregates of **1** to 365 nm light is likely due to an increase in the cis-AB composition of the lipid tails. Cis-ABs have been shown to have larger dipole moments and out-of-plane bending geometry (Figure 1A).^28^ Exposure of **1** to 365 nm light would be expected to result in reduced hydrophobicity and a higher packing parameter.^35^ Such properties may promote the formation of a more liquidlike lamellar lipid assembly compared to the disordered solid aggregate formed by **1** in the absence of light.

Overall, assemblies of **1** display light-dependent assembly characteristics (Figure 1I,J). Under ambient reaction conditions, the self-assembly of **1** is primarily governed by the trans lipid tails, leading to the formation of aggregates. Once the assembly is illuminated by 365 nm light, trans → cis isomerization occurs (Figure 1I), and the assembly is pushed away from its equilibrium state. The newly formed isomers of **1** with cis lipid tails dominate the phase properties of the system, which leads to the spontaneous self-assembly of giant vesicles with well-defined bilayer structures (Figure 1J). However, in the absence of 365 nm irradiation, the system will return to the more thermodynamically stable trans structures, producing less spherical metastable structures (2 h, Figure S3) and, ultimately, resulting in reformation of nonuniform lipid aggregates at equilibrium (after 4 h, Figures 1J and S3).

### Lipid Remodeling in the Presence of Light and Chemical Building Blocks

**Figure 2:**
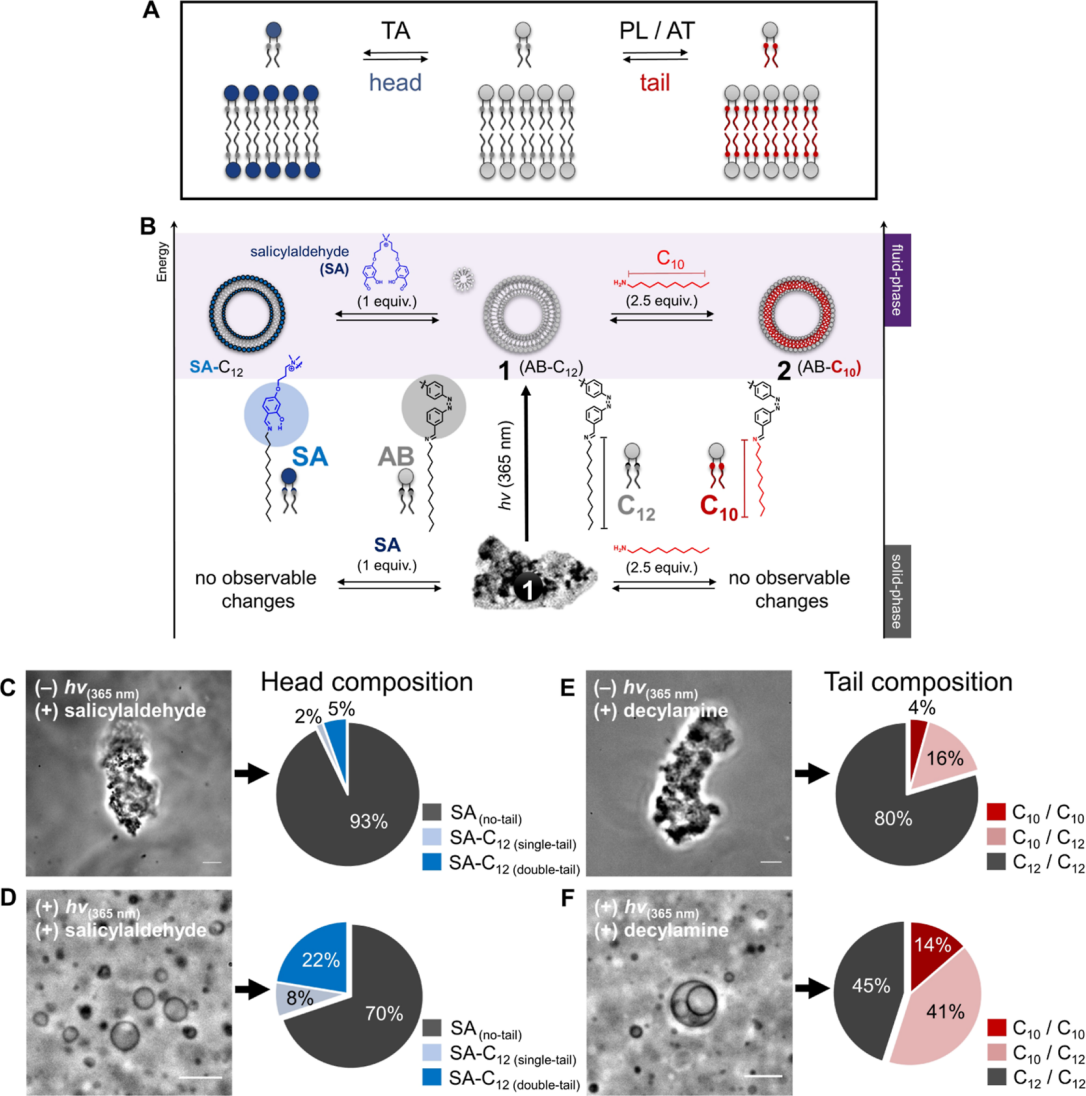
Light-driven remodeling of lipid assemblies through dynamic bond exchange reactions with chemical building blocks. (A) Schematic illustration of enzyme-mediated lipid remodeling processes in biology. TA, transacylase; PL, phospholipase; AT, acyltransferase. (B) Energy diagram of artificial lipid remodeling of assemblies of **1** through reversible imine formation reactions with a synthetic salicylaldehyde derivative (SA, for head-exchange, AB → SA) or decylamine (C_10_, for tail-exchange, C_12_ → C_10_). Top (purple): fluidic phase provided by light activation. Bottom (white): solid phase in the equilibrium state. (C,D) Left: phase-contrast micrographs 24 h after treating **1** with SA (1 equiv) in the absence of light (C) and after light stimulation (D). Scale bars, 10 μm. Right: SA compositions measured by LC-DAD analysis. (E,F) Left: phase-contrast micrographs 24 h after treating **1** with decylamine (2.5 equiv to aldehyde) in the absence of light (E) and after light stimulation (F). Scale bars, 10 μm. Right: exchanged tail compositions calculated from extracted ion chromatograms.

Living cell membranes are highly dynamic structures that are constantly undergoing chemical reactions.^36^ For instance, cell membranes remodel their lipid composition by a series of enzymatic reactions, catalyzed by transacylases, phospholipases, and acyltransferases (Figure 2A).^37^ Lipid remodeling reactions help cells to adjust the membrane properties for subsequent metabolic processes. For instance, cells can exchange lipid head groups or lipid tails to better adapt to temperature changes or to evade cytotoxins.^38,39^ Inspired by these processes in living cells, we characterized how light-responsive membranes of **1**, which possess dynamic imine linkers, remodel their lipid composition when interacting with chemical building blocks (Figure 2B).

To trigger lipid headgroup exchange reactions, we treated **1** with a synthetic salicylaldehyde (SA), which is known to form highly stable imine bonds when compared to simple aldehydes due to intramolecular hydrogen bonding (AB → SA exchange, Figure 2B, left).^40^ Treating aggregates of **1** with SA led to no change in the assembly structure as observed by microscopy (Figure 2C, left), and liquid chromatography–mass spectrometry (LC–MS) analysis showed that <7% of the AB headgroup was exchanged with SA (Figure 2C, right and Table S3). This is presumably due to the restricted access of water-soluble SA amphiphiles in densely packed solid aggregates of trans-**1**. However, if **1** is exposed to 365 nm light to trigger membrane formation during treatment with SA, several giant vesicles are observed under microscopy (Figure 2D, left) and, unlike the transiently stable vesicles consisting of **1**, the remodeled vesicles persisted in the absence of light for 24 h (Figure S6A,B). Additionally, LC–MS showed that under illumination, approximately 22% of the AB headgroup was exchanged with SA (SA-C_12_) (Figure 2D, right, Figure S7 and Table S4).

Next, we explored exchanging the lipid tails of **1** by using excess decylamine (for C_10_ tails) to trigger a transamination reaction (C_12_ → C_10_ exchange, Figure 2B, right). As observed with glycerophospholipids,^41^ we anticipated that even minor changes in tail length could lead to significant differences in phase-related properties for synthetic lipid assembly. Like headgroup exchange, attempting tail exchange reactions in the absence of 365 nm light did not produce observable changes in aggregate morphology or the formation of giant vesicles (Figure 2E, left). LC–MS confirmed the limited efficiency in transamination, suggesting that approximately 12% of the lipid tails are exchanged (Figure 2E, right; Table S5). In contrast, microscopic analysis and extracted ion chromatogram analysis verified that exchange was significantly enhanced under 365 nm light. The newly exchanged products formed giant vesicles containing approximately 34% C_10_-tailed lipids (Figures 2F and S8 and Table S6). As before, the remodeled lipid membranes did not revert to aggregates, even after 24 h (Figure S6C). Overall, our results demonstrate a key advantage of driving the lipid assemblies into a more energetic vesicular state: the greater accessibility that membrane assemblies of **1** have to chemical building blocks, which facilitates dynamic changes in lipid composition.

### Shape-Shifting of Synthetic Cells Driven by Light

**Figure 3:**
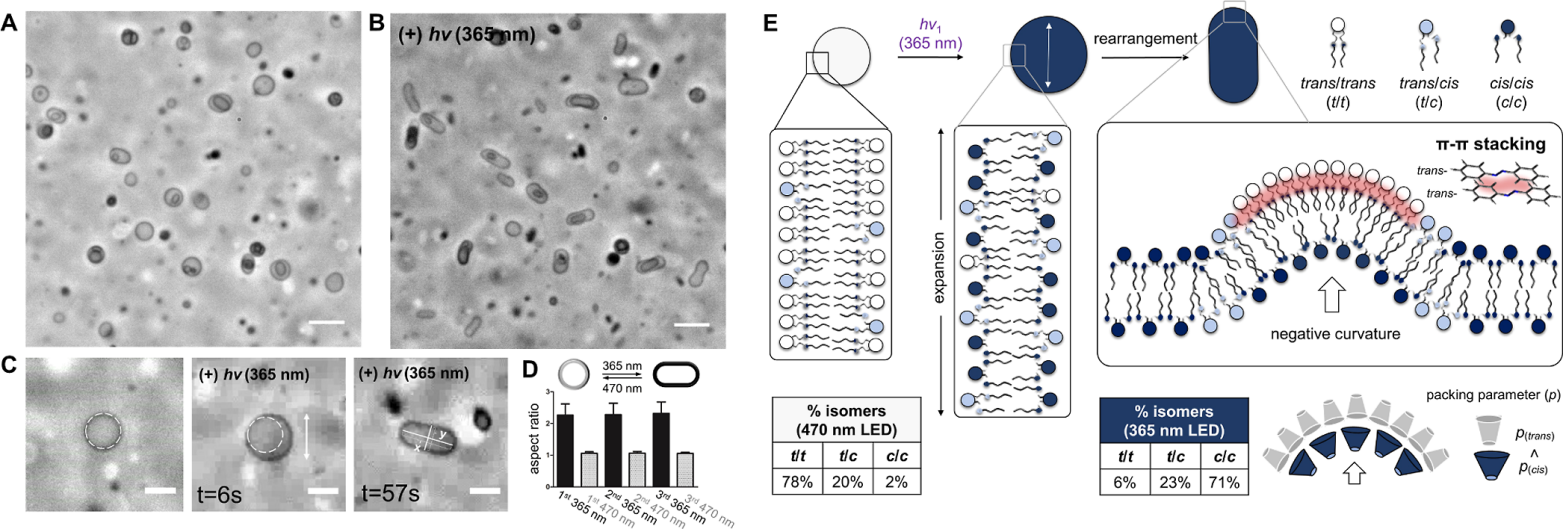
Light-dependent reversible shape-shifting of vesicles consisting of **2**. (A,B) Phase-contrast micrograph of vesicles consisting of **2** under ambient conditions (A) and under 365 nm illumination (B) in HEPES-buffered saline solution (pH 7.5, [HEPES] = 25 mM; [NaCl] = 50 mM). Scale bars, 10 μm. (C) Time-dependent morphology changes of vesicles under 365 nm LED light. Scale bars, 5 μm. (D) Graph of the vesicle aspect ratio (between the minor and major axis) measured from the captured micrographs during three alternating cycles of LED light irradiation (black: under 365 nm, gray: under 470 nm). Error bars indicate SD from each independent measurement (*n* ≥ 45, for each). (E) Scheme of proposed expansion and microdomain formation within membranes in response to the 365 nm illumination. Bottom tables indicate the isomeric ratio under the corresponding LED illumination (white: 470 nm, blue: 365 nm) calculated from LC analysis.

Membranes within cells are characterized by the ability to change their collective shape and structure in response to environmental stimuli, primarily through the action of proteins that require the input of chemical energy.^42^ Synthetic membranes energized by light may also be able to access morphologies that are not easily attained at equilibrium. Intriguingly, after light-stimulated giant vesicles of **1** underwent lipid tail exchange, forming C_10_-tailed lipids, we noticed that the newly remodeled vesicles exhibited striking reversible morphological changes in response to 365 and 470 nm LED lights (Figure S9). To further characterize this unexpected phenomenon, we generated pure **AB-C**_**10**_ lipids, referred to as **2**, by performing an in situ reaction between AB-aldehyde (**AB**_**CHO**_) and decylamine (C_10_), characterizing the product through reductive amination (Figure S10A,B). In situ formation of **2** led to spontaneous assembly of spherical giant vesicles, which could be observed by light microscopy and cryo-EM analysis (Figures 3A and S10C). In addition, the small-angle X-ray scattering (SAXS) profile, DLS analysis, and the size distribution for giant vesicles were further evaluated (Figure S10D–F). The isomer analysis of **2** demonstrated that under 365 nm light, the approximate ratio of trans:cis was 18:82, whereas under 470 nm light, it was 88:12 (Table S7). When illuminated under 365 nm light, distinctive morphological changes in vesicles consisting of **2** were observed by phase-contrast microscopy (Figure 3B,C). Spherical vesicles first expanded their surface area by approximately 30% (Figure S11 and Movie S2). This membrane expansion was then subsequently followed by the formation of a prolate rod-shaped vesicle (Figure 3C). When we applied the 470 nm LED light, the prolate vesicle switched back to a spherical shape (Figure S12A). We observed an average aspect ratio shift from 1.06 ± 0.05 to 2.29 ± 0.36 (Figure 3D and S12B), and this behavior was highly reversible in response to repeated exposure to alternating wavelengths of UV and visible light (Movie S3).

While it is challenging to ascertain why such reversible shape changes are being observed, previous studies on AB-based supramolecular assemblies have demonstrated that planar trans-ABs exhibit stronger intermolecular interactions due to π–π stacking.^43–45^ Therefore, it is possible that the observed shape changes are due to the formation of lipid microdomains triggered by the trans → cis isomerization. Under 365 nm irradiation, the enriched cis isomers (82%, Table S7), due to their higher packing parameter (Figure S13),^35^ initially trigger the observed lateral expansion of the membrane leaflets, resulting in a transient expansion of the overall surface area of the light-activated membranes (Figure 3E, middle). However, this expansion is presumably immediately followed by the spontaneous formation of lipid microdomains by the remaining trans isomers (18%), driven by stronger π–π stacking interactions (Figure 3E, right). Rearrangement of lipids, for instance through lipid flip-flop, could result in localized regions of negative curvature since the membrane leaflet opposing the trans microdomains would be composed of the more abundant cis isomers that are predicted to have a wider cone shape (Figure 3E, right bottom, and Figure S13B). Due to the curved domains, vesicles may then spontaneously adopt the observed prolate structure. It is also plausible that the reduction in membrane rigidity induced by light may facilitate lipid reorganization and subsequent shape-shifting phenomena.

### Formation of Synthetic Tissues Using Multivalent Polymers and Light

**Figure 4:**
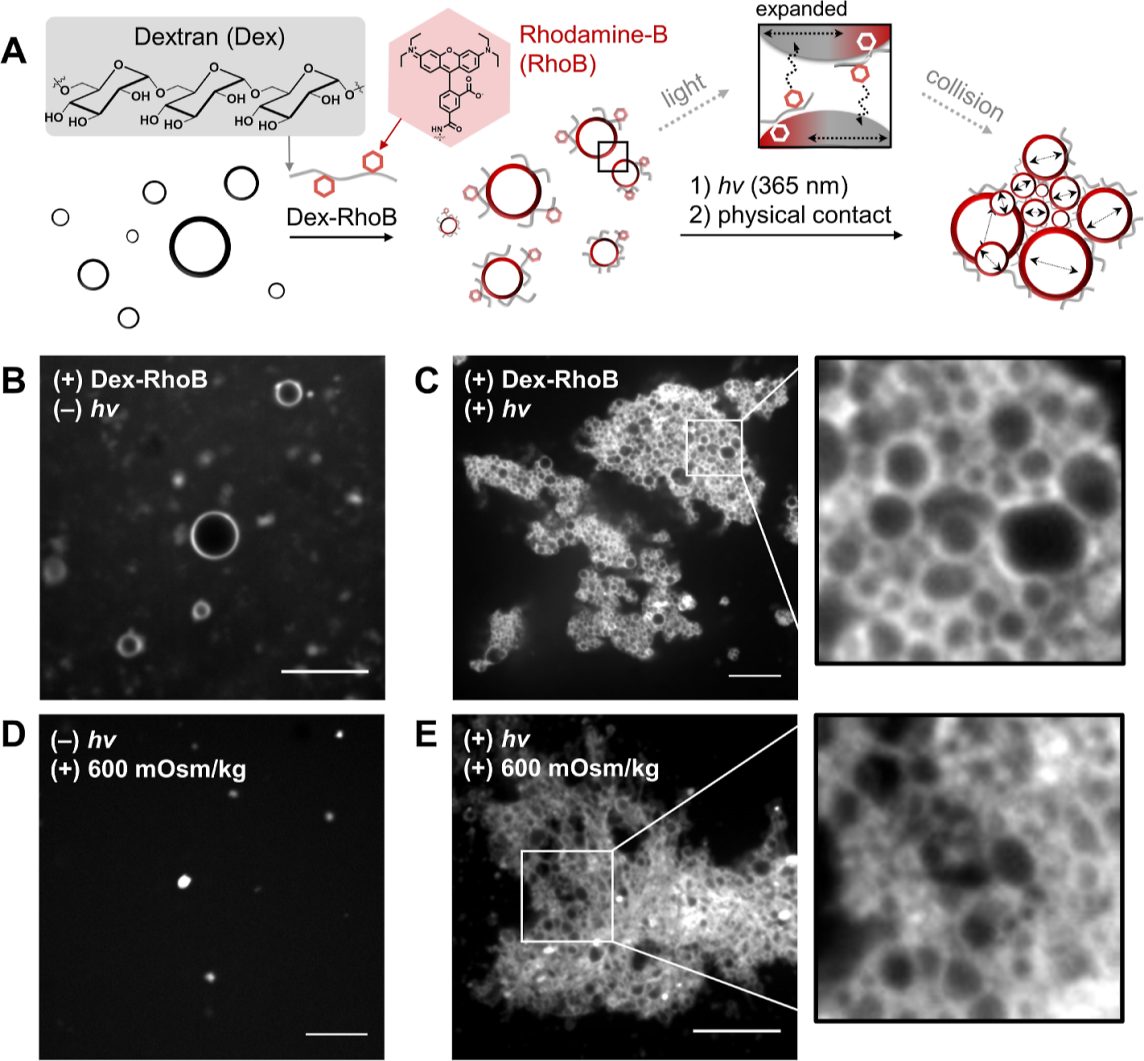
Light-driven membrane interactions mediated by multivalent polymers leading to the formation of higher-order synthetic tissues. (A) Scheme of the proposed process of synthetic tissue formation. First, the surfaces of the vesicles of **2** are saturated by dextran-rhodamine B (Dex-RhoB) polymers. Then, the modified membranes undergo light-driven membrane expansion followed by intermembrane cross-linking mediated by surface-bound Dex-RhoB (inset scheme), forming multivesicular assemblies. (B) Confocal fluorescence micrograph of vesicles of **2** after treatment with 5 mol % of Dex-RhoB. Scale bars, 10 μm. (C) Confocal fluorescence micrographs of synthetic tissues produced from vesicles of **2** after application of 5 mol % Dex-RhoB and 365 nm LED illumination (3 min), followed by physical agitation through centrifugation at 132,000 relative centrifugal force (rcf) for 5 min. Scale bars, 10 μm. Inset: an enlarged image of a local area in (C). Size, 10 μm × 10 μm. (D, E) Confocal fluorescence micrographs of individual vesicles (D) or synthetic tissues (E) after being exposed to 0.6 M glucose solution, which has a higher osmotic pressure compared to the initial self-assembly condition (Δ 603 mOsm/kg). Scale bars, 10 μm. Inset: An enlarged image of a local area in (E). Size, 10 μm × 10 μm.

Membranes consisting of **2** have significant aromatic character, and we observed spontaneous binding between AB-based membranes and charged aromatic compounds, including many fluorescent dyes (Figure S14A–D). We believe that π stacking interactions are partially responsible for causing such aromatic molecules to be trapped in the hydrophobic domains of the lipid bilayers^46,47^ since many of the dyes that stained the membranes formed by **2** do not stain lipid membranes that lack aromatic groups. In contrast, nonaromatic neutral macromolecules, such as dextran and polyethylene glycol, could be encapsulated into the hydrophilic vesicle interior, which could be readily observed by phase-contrast microcopy due to the difference in the refractive index (Figure S14E–H).^48^ Based on these observations, we speculated that a polymer such as dextran, chemically conjugated to multiple Rhodamine B dye molecules (Dex-RhoB), would be able to bind to vesicles of **2** (Figure 4A). Indeed, initial test results demonstrated that 5 mol % Dex-RhoB readily binds to the surface of vesicles of **2** in the absence of light, and this binding can be visualized by fluorescence microscopy (Figure S15A). However, we were surprised to find that subsequent irradiation with 365 nm light led to cross-linking of Dex-RhoB-treated vesicles of **2**, producing multivesicular assemblies (Figure S15A and Movie S4). This phenomenon was found to be dependent on the concentration of Dex-RhoB; concentrations of 8 mol % Dex-RhoB or higher produced fluorescently labeled giant vesicles, but irradiation with light led to no vesicle cross-linking (Figures S15B and S16). In contrast, adding 2 mol % Dex-RhoB or less led to cross-linking even in the absence of UV light. Overall, it was found that the light-driven cross-linking occurred when using 4–6 mol % Dex-RhoB, which presumably represents the optimal concentration of Dex-RhoB that can fully cover the vesicle surfaces without inducing multiple polymer-to-vesicle interactions in the absence of light. We also confirmed that treating vesicles either with rhodamine B or with dextran separately did not result in light-driven multivesicular assemblies (Figure S15C,D). Based on these observations, we speculate that the light-triggered expansion in the membrane surface area, as depicted in Figure 3E, facilitates intermembrane cross-linking for vesicles that bind Dex-RhoB. Interaction would occur between the light-stimulated membranes and free-RhoB on the surface of adjacent vesicles (Figure 4A, inset).

To create light-driven multivesicular assemblies at various length scales, we first prepared Dex-RhoB-coated (5 mol %) membranes of **2** and confirmed that individual vesicles exhibited surface fluorescence (Figure 4B). Irradiation with 365 nm LED light, followed by physical agitation, such as tumbling, vortexing, or centrifugation, led to the formation of tissue-like vesicular assemblies (Figures 4C and S17A).^49^ In many cases, we could generate vesicle networks that are over 100 μm in length (Figure S18). As observed by fluorescence microscopy, the synthetic tissues displayed clear boundary structures consisting of interconnected fluorescently labeled giant vesicles (Figure 4C, inset; Figure S18, inset). In contrast, in the absence of light, no notable networks were observed (Figure S17B).

Living cells often form intercellular networks to gain survival advantages. For instance, microorganisms form biofilms where individual cells are often cross-linked by natural polymers, such as polysaccharides, proteins, and DNA. Biofilms often protect cell populations from external chemical and physical challenges.^50,51^ By analogy, we investigated whether the formed multivesicular assemblies could also exhibit greater resistance to disruptive physical forces, such as osmotic pressure, compared to isolated vesicles. We performed a side-by-side comparison between separated vesicles and their light-driven synthetic tissues under various osmotic pressures. Both vesicles and cross-linked tissues were initially prepared in buffered saline solution having a measured osmolality of 121 mOsm/kg (Table S8). We then applied an osmotic shock by adding the assemblies to glucose solutions (10-fold volume) that have higher osmolality, ranging from 214 to 724 mOsm/kg (Figure S19 and Table S8). Treating isolated vesicles with a 0.4 M glucose solution (Δ 388 mOsm/kg) led to a largely denatured structure (Figure S19A). When vesicles were exposed to 0.6 M glucose solution (Δ 603 mOsm/kg, Figure 4D), no vesicles were observable after 30 min. In contrast, the synthetic tissues can maintain their structure even against an osmotic stress of 600 mOsm/kg (Figures 4E and S19B). Mimicking a function of microbial biofilms, the synthetic matrix created by Dex-RhoB appears to provide additional mechanical benefits by cross-linking and supporting the membranes, thereby highlighting the advanced durability and function of the tissue-like assembly in withstanding external stress.

### Stepwise One-Pot Transformation of Disordered Lipid Aggregates into Synthetic Tissues

**Figure 5:**
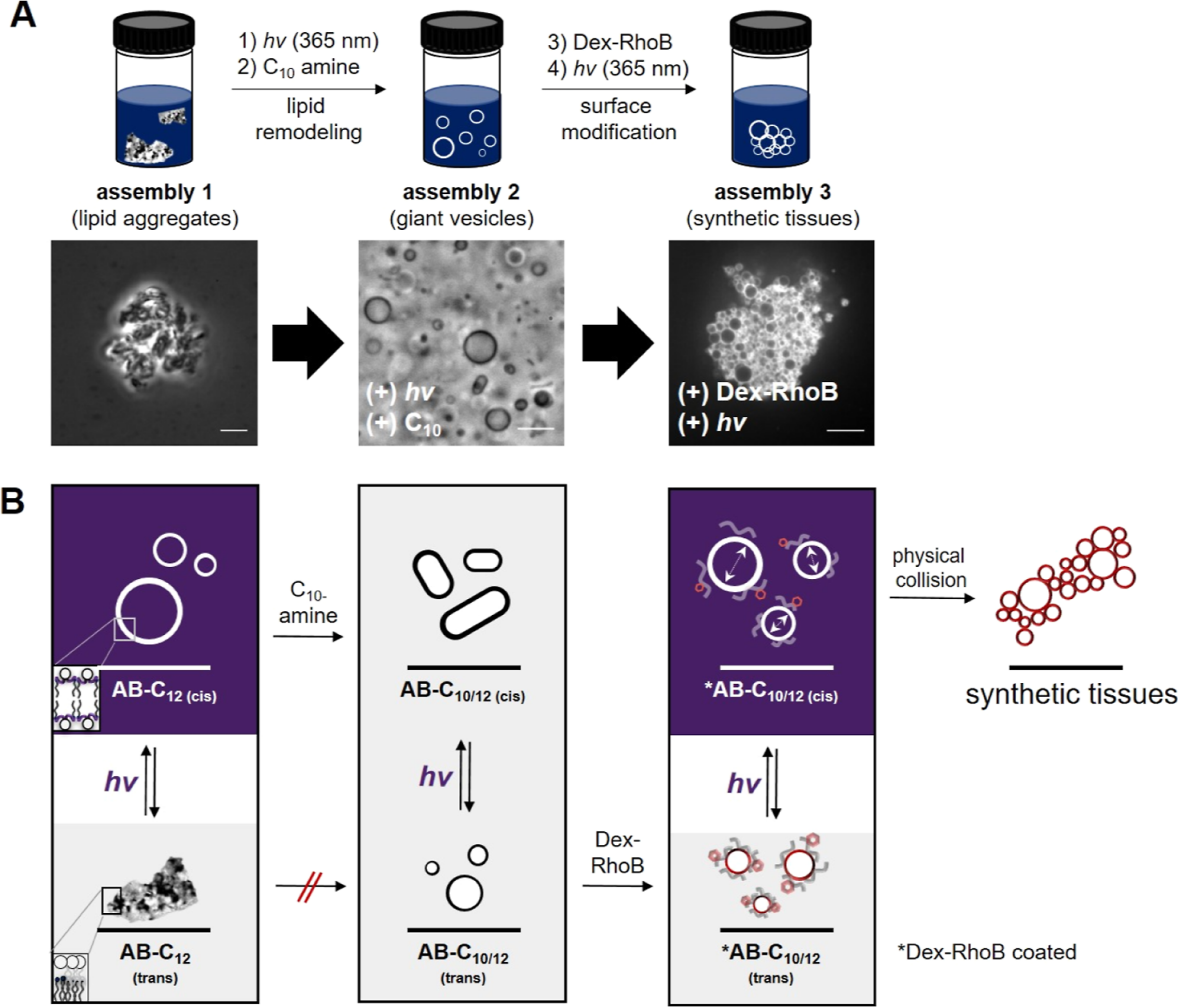
Stepwise one-pot transformation of AB-based lipid assemblies via a series of coupled interactions between light and chemical building blocks. (A) Top: scheme depicting the conversion of disordered lipid aggregates into highly ordered synthetic tissues. Bottom: phase-contrast and fluorescent micrographs that were captured at each stage of the assembly process. Scale bars: 10 μm. (B) Flow diagram depicting the hierarchical transformations achieved through coupling 365 nm light stimulation with addition of chemical building blocks. Purple shading denotes the light-driven stages, leading to subsequent higher-order transformations.

Given our observations, we decided to explore if the one-pot hierarchical transformation, from disordered lipid aggregates to synthetic tissues, could be possible through a series of manipulations using both light energy and synthetic metabolites (Figure 5A). In the first step, illuminating solid aggregates of **1** with 365 nm light and adding a C_10_ amine precursor led to a lipid remodeling process not possible in the absence of light (Figures 5A,B and S20A). As already discussed, exposing **1** to 365 nm light promotes reaction with exogenously added precursors, forming membrane-bound vesicles (Figure 5B, middle). The resulting system, now consisting of a significant fraction of **2**, formed membrane assemblies that did not revert to the aggregated state (Figure S20B,C). The composition and subsequent shape of the newly remodeled membranes can be reversibly controlled by 365 and 470 nm LED lights (Figure S20C,D). Subsequent addition of the multivalent polymer, Dex-RhoB, produced fluorescent membranes, reflecting the noncovalent interaction between the polymer and the light-sensitive membranes (Figure S20E). Upon the application of a second light stimulus, the modified membranes underwent surface expansion, followed by cross-linking with adjacent membranes mediated by surface-bound multivalent polymers, finally producing higher-order tissue-like synthetic structures (Figure 5A,B, right, and Figure S20F).

## Conclusions

We have shown that the combined input of light energy and chemical stimuli allows the stepwise and hierarchical transformation of disordered lipid aggregates into organized compartments and ultimately complex tissue-like vesicular networks. Simple lipid aggregates can transform into assemblies with substantial complexity if nonequilibrium transitions are facilitated. It is tempting to speculate that such coupled mechanisms might have existed during the early evolution of cells, dynamically forming higher-order structures. Such protocellular assemblies may have enjoyed survival advantages, for instance, against physical or chemical injury. Our work suggests that coupling multiple energetic inputs to elaborate supramolecular assemblies could lead to the creation of advanced synthetic cells that more closely resemble the assembly behavior found in living systems.

## Supporting Information Available

Materials and methods, experimental details, chemical synthesis, and NMR spectra (PDF)
**Movie S1.** Time-lapse video showing the transition of in situ formed disordered lipid aggregates of compound **1** (**AB-C_12_**) into fluidic membrane-bound assemblies under exposure to 365 nm LED light for 3 min (MP4)
**Movie S2.** Time-lapse video depicting the temporary expansion of in situ formed vesicles (compound **2**, **AB-C_10_**) under 365 nm LED light for the initial 10 s (MP4)
**Movie S3.** Time-lapse video illustrating the reversible shape changes of in situ formed vesicles consisting of compound **2** (**AB-C_10_**) under alternating illumination of 365 and 470 nm LED light (MP4)
**Movie S4.** Time-lapse video demonstrating light-induced cross-linking of vesicles consisting of **2** (**AB-C_10_**) treated with 5 mol % of Dex-RhoB polymer (MP4)
