# Supplementary material for: Light-Driven Membrane Assembly, Shape-Shifting, and Tissue Formation in Chemically Responsive Synthetic Cells
Source: J Am Chem Soc. 2023 Nov 14;145(47):25815–23. doi: 10.1021/jacs.3c09894 (PMC10690792; doi:10.1021/jacs.3c09894)
Supplement: Supplementary file 1 — ja3c09894_si_001.pdf [file ja3c09894_si_001.pdf]

Supporting Information

Light-driven membrane assembly, shape-shifting, and tissue formation in chemically responsive synthetic cells

Youngjun Lee[1], Alessandro Fracassi[1], Neal K. Devaraj[1*]

[1]Department of Chemistry and Biochemistry, University of California, San Diego, 9500 Gilman Drive, La Jolla, California 92093, United States.

*Corresponding author: ndevaraj@ucsd.edu

Table of Contents

1. Materials and Methods……………………………………………………....... S2
   1.1. General Information………………………………………………....... S2
   1.2. Experimental procedures……………………………………………... S3
2. Supplementary Text…………………………………………......................... S8
   2.1. Extended technical descriptions……………………………………….. S8
   2.2. Synthetic procedures…………………………………….................. S11
3. Appendix: NMR spectra………………………………….................. S24
4. Figures S1 to S20……………………………….................................. S36
5. Tables S1 to S8………………………………….................................. S56
6. Captions for Movies S1 to S4…………………………………............ S60
7. References………………………………….. S61

1. Materials and Methods

1.1. General Information

1.1.1. Materials

Chemicals and solvents were purchased from Sigma-Aldrich, Tokyo Chemical Industry Co., Ltd, Thermo Fisher Scientific, or Combi-blocks Inc, and used without further purification unless otherwise specified. Thin-layer chromatography (TLC) was carried out on E. Merck silica gel 60 F254 analytical plates. Chemical synthesis was monitored by a handheld UV lamp (254 nm). Column chromatography was carried out using Merck silica gel (60 Å, 230−400 mesh, particle size 0.040−0.063 mm) with technical grade solvents. Fluorescent dyes were purchased from the following commercial vendors: Rhodamine B and Cy3-alkyne were obtained from Sigma-Aldrich (catalogue numbers: 83689 and 777331). SynaptoGreen was purchased from AAT bioquest (catalogue number: 21483). Dextran-Rhodamine B conjugate (10,000 MW, Neutral) was purchased from Thermo Fisher Scientific (catalogue number: D1824).

1.1.2. Nuclear magnetic resonance (NMR) spectroscopy

^{1}H and ^{13}C NMR spectra were recorded on Bruker AVA-300 or Varian VX-500 MHz instruments. Chemical shifts were reported in parts per million (δ) and calibrated using internal tetramethylsilane (TMS) standard for ^{1}H NMR spectra or solvent residual signals [chloroform-*d* 7.26 ppm; methanol-d_4 3.31 ppm; dimethyl sulfoxide (DMSO)-d_6 2.50 ppm]. For ^{13}C NMR spectra, calibration was performed using solvent residual signals [chloroform-*d* 77.16 ppm; methanol-d_4, 49.00 ppm; DMSO-d_6 39.52 ppm].[S1] Multiplicity was indicated as follows: s (singlet); d (doublet); t (triplet); q (quartet); qui (quintet); m (multiplet); dd (doublet of doublet); dt (doublet of triplet); brs (broad singlet), etc. Coupling constants were reported in Hz.

1.1.3. Liquid Chromatography-Mass Spectrometry (LC-MS)

LC-MS analysis was performed using an Agilent 1260 Infinity LC System coupled with an Agilent 6120 quadrupole mass spectrometer. The LC system was equipped with a diode array detector (DAD), an evaporative light scattering detector (ELSD), and an Eclipse Plus C8 analytical column. The analysis employed a mixture gradient of solvents as follows: Phase A: H_2O with 0.1% formic acid; Phase B: MeOH with 0.1% formic acid. In all experiments, 20 μL of the corresponding sample was injected.

1.1.4. High-Resolution Mass Analysis (HRMS)

HRMS was performed at UCSD Molecular Mass Spectrometry Facility using an Agilent 1260 Infinity Binary LC coupled with a 6230 Accurate-Mass TOFMS system for HPLC-HR-ESI/APCI-MS analysis.

1.1.5. Microscopy

Phase contrast imaging was performed using an Olympus BX51 optical microscope equipped with a phase contrast condenser (Ph3) and a 100x oil immersion objective. Images were

captured with an ORCA-spark Digital CMOS camera (C11440-36U, Hamamatsu) using Olympus cellSens 3.1 imaging software. To obtain the movie of fluorescently labeled vesicles (movie S4), a fluorescence mercury light source (U-LH100HGAPO, Olympus) was used, coupled with a Texas Red filter cube. Confocal fluorescence analysis was conducted using a Zeiss Axio Observer Z1 motorized inverted fluorescence microscope (Carl Zeiss Microscopy) with a CSU-X1 confocal scanner unit (Yokogawa). A 63x, 1.40 NA oil immersion objective was employed. The respective fluorophores were excited using diode lasers at wavelengths of 405 nm, 488 nm, 561 nm, and 640 nm. Images were captured with an ORCA-Flash4.0 V2 Digital CMOS camera (Hamamatsu) using ZEN Blue 2.3 imaging software (Carl Zeiss Microscopy). In general, samples were prepared by dropping 2 µL of the sample on a microscope glass slide (Fisherbrand), and covered with a coverslip. The obtained images were further analyzed with ImageJ software.

1.1.6. Light Sources

Irradiation with 365 nm light was carried out using an EvoluChem™ LED (9 mW/cm^2, HepatoChem). Handheld 365 nm (Alonefire SV003) and 470 nm flashlights (UltraFire H-B3) were used in the case of acquiring real-time images and movies S1 to S4. For room lighting, a 32 W, 4100 K T8-shape fluorescent bulb (F32T8/SPX41/ECO2, GE) was used.

1.1.7. Instruments for Absorption and Osmolality Analysis

UV-Vis measurements were conducted with a NanoDrop 2000c spectrophotometer (Thermo Scientific), using standard quartz glass cuvettes with a path length of 10 mm and a volume of 3500 µL (Thorlabs, Inc.). The Osmolality of the samples was determined using the Advanced Micro-Osmometer Model 3300 (Advanced Instruments).

1.1.8. Statistical Methods

Statistical analysis was conducted using Prism 5 software. Error bars indicate the standard deviation obtained from a minimum of three replicates.

1.2. Experimental procedures

1.2.1. General procedure for in situ imine formation

In 2 mL Eppendorf tubes, stock solutions of the following were prepared: 2 mM **AB$_{CHO}$** in H_2O, and 8 mM alkylamines (C_{10} or C_{12}) in HEPES buffer (50 mM at pH 7.5), and 200 mM NaCl in HEPES buffer (50 mM at pH 7.5). Before the mixing process, certain pretreatment steps were performed. The stock solution of **AB$_{CHO}$** was subjected to UV treatment for 3 minutes, followed by heating at 50 °C for 3 minutes. This treatment was carried out to disassemble any pre-formed assemblies present in the solution. Prior to use, the **AB$_{CHO}$** stock was cooled to room temperature and treated with 470 nm light for 3 minutes to ensure that the **AB$_{CHO}$** stock was at equilibrium under ambient conditions. Likewise, the stock solution of alkylamines underwent heating at 50 °C for 3 minute then allowed to cool to room temperature to ensure the preparation of a well-dissolved homogeneous stock solution.

To promote the in situ formation of **AB-C$_{12}$**, a mixture was prepared by combining 40 µL of 2 mM **AB$_{CHO}$** in H_2O, 20 µL of 8 mM dodecylamine (C_{12}) in HEPES buffer, and 20 µL of 200 mM NaCl in HEPES buffer in 2 mL glass vials. The total concentration of each component in the 80 µL reaction mixture is as follows: 1 mM **AB$_{CHO}$**, 2 mM C_{12}-amine, 25 mM HEPES (at pH 7.5), and 50 mM NaCl. The resulting mixture was left to tumble overnight using a tube rotator set at 15 revolutions per minute (rpm) for 20−24 hours.

Similarly, for the in situ formation of **AB-C$_{10}$**, a mixture was created by mixing 20 µL of 2 mM **AB$_{CHO}$** in H_2O, 20 µL of blank H_2O, 20 µL of 8 mM decylamine (C_{10}) in HEPES buffer, and 20 µL of 200 mM NaCl in HEPES buffer in glass vials (2 mL). The total concentration of each component in the 80 µL reaction mixture is as follows: 0.5 mM **AB$_{CHO}$**, 2 mM C_{10}-amine, 25 mM HEPES (at pH 7.5), and 50 mM NaCl. The mixture was left to tumble overnight using a tube rotator set at 15 rpm for 20−24 hours.

For imaging analysis, samples were prepared by placing 1.5–2 µL of the relevant reaction mixture onto a microscope glass slide and then covering it with a coverslip. To confirm the complete consumption of **AB$_{CHO}$** and the formation of two-tailed imine compounds using LC-MS analysis, an additional reduction step was performed using sodium borohydride (see '2.1. Extended technical descriptions' section).

1.2.2. General procedure for light irradiation

A 365 nm LED light (9 mW/cm²) was directed onto glass vials containing the corresponding lipid samples for a period of 3 minutes. The transmittance of the borosilicate glass vials at this wavelength exceeds 90%.[S2] The standing light source was positioned at an approximate distance of 20 cm from the vials. To capture real-time images or movies of lipid assemblies during the light-activation process (S1 to S4), handheld 365 nm and 470 nm flashlights were positioned on a tabletop stand adjacent to the light microscope and directed onto the glass slide containing the sample. The distance between the sample and the flashlights was maintained at over 15 cm. It was confirmed that any possible heat impact arising from the irradiation was insignificant (see '2.1. Extended technical descriptions' section). For the samples that were stored at ambient conditions, sample containing glass vials (2 mL) were tumbled using a tube rotator at a temperature of 20 °C under room lighting (32 W, 4100 K T8-shape fluorescent bulb) until the samples were collected for imaging or other analysis.

1.2.3. Time-dependent *cis*→*trans* conversion of light activated AB-C$_{12}$ under ambient conditions

The *cis* form of **AB-C$_{12}$** was generated by exposing the in situ-formed **AB-C$_{12}$** solution [**AB$_{CHO}$**:C_{12}-amine = 1:2 mM, in a HEPES-buffered solution (25 mM) containing 50 mM NaCl at pH 7.5] to a 365 nm LED light for 3 minutes. After irradiation was stopped, the samples were tumbled at room temperature (20 °C) and under normal room lighting (32 W-4100 K-fluorescent bulbs, 250–350 lux). For kinetic analysis, at specific time intervals (0 min, 15 min, 30 min, 45 min, 1 hour, 1.5 hours, 2 hours, 4 hours, 8 hours, and 24 hours), 40 µL samples were taken and mixed with 40 µL of methanol. The mixture was then vortexed for 10 seconds in a dark environment to ensure the dissolution of any potential lipid aggregates. Subsequently, the

samples underwent LC-MS analysis. The entire measurement process was conducted in triplicate.

1.2.4. Exchange of isolated AB-C_{12} with a salicylic aldehyde derivative (SA head)

In 2 mL Eppendorf tubes, stock solutions of the following were prepared: 4 mM isolated **AB-C_{12}** (see '2.2. Synthetic procedures' section) in methanol, 4 mM **SA** in H_2O, 200 mM NaCl in H_2O, and a blank HEPES buffer (50 mM at pH 7.5).

To an empty 2 mL glass vial, 20 µL of the 4 mM **AB-C_{12}** stock solution was added, followed by blowing a stream of argon gas to form a thin lipid film. The vial was further dried under high vacuum for 1 hour.

To a 2 mL vial containing the previously dried lipid film of **AB-C_{12}**, 20 µL of 4 mM **SA** in H_2O, 20 µL of 200 mM NaCl in H_2O, and 40 µL of the HEPES blank were added. The total concentration of each component in the 80 µL reaction mixture is as follows: 1 mM **AB-C_{12}**, 1 mM **SA**, 25 mM HEPES (at pH 7.5), and 50 mM NaCl. For the control condition, the vial was tumbled overnight (24 hours) without any further treatment. For the light-driven condition, irradiation with a 365 nm LED light was performed for 3 minutes, followed by covering the sample with aluminum foil, and tumbling overnight (24 hours). The resulting mixtures were analyzed using LC-MS to validate the relative ratios of unreacted **SA**, single-tailed **SA**, and double-tailed **SA**. This was achieved by integrating the area under the curve of the chromatograms at 230 nm using a LC-DAD method (see '2.1. Extended technical descriptions' section). The entire measurement process was conducted in triplicate.

1.2.5. Exchange of isolated AB-C_{12} with decylamine ($C_{12} \rightarrow C_{10}$ exchange)

In 2 mL Eppendorf tubes, stock solutions of the following were prepared: 4 mM isolated **AB-C_{12}** in methanol, 20 mM decylamine (C_{10}) in HEPES buffer (50 mM at pH 7.5), a blank solution containing 16.6 mM HEPES with 66.6 mM NaCl at pH 7.5. The same **AB-C_{12}** lipid film was prepared as described in section 2.4.

To a 2 mL vial containing the previously dried lipid film of **AB-C_{12}**, 60 µL of previously prepared blank solution and 20 µL of 20 mM C_{10}-amine stock were added. The total concentration of each component in the 80 µL reaction mixture is as follows: 1 mM **AB-C_{12}**, 5 mM C_{10}-amine, 25 mM HEPES (at pH 7.5), and 50 mM NaCl. For the control condition, the vial was tumbled overnight (24 hours) without any further treatment. For the light-driven condition, irradiation with a 365 nm LED light was performed for 3 minutes, followed by covering it with aluminum foil, and tumbling overnight (24 hours).

The resulting mixtures underwent analysis using LC-MS to confirm the relative ratios of lipid products with two C_{10} tails (C_{10}/C_{10}), one C_{10} and one C_{12} tail (C_{10}/C_{12}), and two C_{12} tails (C_{12}/C_{12}), by measuring their relative mass abundance. This analysis involved the following steps: 1) A post-treatment solution was prepared, consisting of 100 mM $NaBH_4$ and 100 mM of cetyltrimethylammonium bromide (CTAB, see '2.1. Extended technical descriptions' section) in methanol. 2) The reaction mixture (80 µL) was combined with the post-treatment solution (80 µL) and subjected to sonication for 20–30 seconds. 3) The resulting sample was exposed

to 365 nm LED light for 1 minute, followed by an additional 1 minute under 470 nm light (see ‘2.1. Extended technical descriptions’ section). 4) Then the sample was subjected to LC-MS analysis.

1.2.6. Encapsulation test using AB-C_{10} vesicles

The capturing of aromatic compounds by **AB-C_{10}** membranes was verified through the following procedure. A stock solution of Rhodamine B, Cy3-alkyne, SynaptoGreen, and Doxorubicin at a concentration of 50 μM in H_2O was prepared. Then, 20 μL of an in situ **AB-C_{10}** solution (**AB_{CHO}** 0.5 mM scale) was combined with 2 μL of the prepared dye stock solution (ratio of **AB-C_{10}**:dye = 100:1 mol%). The mixture was gently vortexed for 5 seconds and then 2 μL of the mixed sample was applied onto a glass slide for imaging without any additional treatment.

To demonstrate the encapsulation of dextran (average molecular weight: 10,000) or polyethylene glycol (PEG 8000, average molecular weight: 8,000) within vesicles, the following steps were conducted: 2 mM of **AB_{CHO}** in H_2O, and 8 mM decylamine (C_{10}) in HEPES buffer (50 mM at pH 7.5), 200 mM NaCl in HEPES buffer (50 mM at pH 7.5), and 20% (w/v, weight/volume percentage) stock solution of PEG 8000, or dextran in H_2O were prepared. In an empty 2 mL glass vial, 20 μL of 2 mM **AB_{CHO}** in H_2O, 20 μL of 8 mM C_{10}-amine in HEPES buffer, 20 μL of 200 mM NaCl in HEPES buffer, and 20 μL of the aforementioned H_2O stock solution (Dextran or PEG 8000) were combined. The total concentration of each component is as follows: 0.5 mM **AB_{CHO}**, 2 mM C_{10}-amine, 5% (w/v) dextran or PEG 8000, 25 mM HEPES, and 50 mM NaCl. The mixture was gently agitated overnight (for over 20 hours). Subsequently, 2 μL of the resulting solution was mixed with 20 μL of 25 mM HEPES blank for a 1 to 10 dilution. The resulting diluted solution (2 μL) was placed onto a glass slide for image analysis.

To obtain phase contrast images of dextran or polyethylene glycol within the external vesicular space, a general in situ **AB-C_{10}** solution (**AB_{CHO}** 0.5 mM scale) and a 5% (w/v) stock solution of dextran or PEG 8000 in HEPES buffer (25 mM at pH 7.5) were prepared. Subsequently, 2 μL of the in situ solution was mixed with 20 μL of the stock solution of dextran or PEG 8000 to achieve a 1 to 10 dilution. The resulting mixed solution (2 μL) was placed onto a glass slide for image analysis.

1.2.7. General procedure for the preparation of multi-vesicular synthetic tissues using Dextran-Rhodamine B (Dex-RhoB) and light

An in situ solution of **AB-C_{10}** (at a concentration of 0.5 mM **AB_{CHO}**) and a stock solution containing 0.5 mM of Dex-RhoB (average molecular weight 10,000, with a degree of Rhodamine B substitution of 2) in H_2O was prepared. After overnight in situ reaction to generate giant vesicles, 40 μL of the in situ **AB-C_{10}** solution was transferred to an Eppendorf tube, followed by the addition of 2 μL from the 0.5 mM Dex-RhoB stock solution (equivalent to 5 mol%). The combined mixture was gently vortexed for 5 seconds. Subsequently, light irradiation using a 365 nm LED was conducted for 3 minutes, after which the mixture was subjected to centrifugation at 132,000 relative centrifugal force (rcf) for 5 minutes. The

separated layer was homogeneously resuspended using gentle pipetting techniques. The resulting sample was then utilized for image analysis or other experiments. For the control experiments, modifications to the Dex-RhoB concentration or the utilization of various physical agitation methods (tumbling and vortexing) were implemented. Despite these alterations, the overall protocol remained consistent with the description provided in this section.

1.2.8. Stability test of vesicles and synthetic tissues under varying osmotic pressures

Stock solutions with varying glucose concentrations (ranging from 0 M to 0.6 M) in a HEPES-buffered saline solution (containing 25 mM HEPES and 50 mM NaCl at pH 7.5) were prepared in 2 mL Eppendorf tubes. The osmolality (mOsm/kg) of each glucose stock solution was measured and recorded. To prepare vesicle samples, in situ **AB-C$_{10}$** solution was prepared as described in section 2.1 (at a scale of 0.5 mM **AB$_{CHO}$** in 25 mM HEPES and 50 mM NaCl at pH 7.5). Subsequently, 5 mol% of Dex-RhoB was added to generate fluorescently labeled giant vesicles, which were used for the vesicle stability test. To prepare synthetic tissues, light irradiation (3 min) and centrifugation (5 min) were additionally performed following the previous protocols. To apply osmotic pressure, 4 μL of either the vesicle or tissue sample was combined with 40 μL of the corresponding glucose stock solution in 0.6 mL Eppendorf tubes. The mixture was then gently mixed using pipetting techniques. The resulting mixture was left to rest for minimum 0.5 hour. Afterward, each sample was examined using microscopy.

1.2.9. One-pot sequential transformation of AB-C$_{12}$ lipid aggregates into synthetic tissues

The subsequent stock solutions were prepared in 2 mL Eppendorf tubes: 2 mM isolated **AB-C$_{12}$** in methanol, 20 mM decylamine (C$_{10}$) in HEPES buffer (25 mM at pH 7.5), 1 mM Dex-RhoB in H_2O, and a HEPES-buffered saline solution (containing 25 mM HEPES and 50 mM NaCl at pH 7.5).

In an empty 2 mL glass vial, 20 μL of 2 mM **AB-C$_{12}$** stock solution was introduced, and a stream of argon gas was blown to generate a thin lipid film. The vial was further dried under high vacuum for 1 hour.

To facilitate the initial step of the reaction (lipid exchange), 40 μL of HEPES-buffered saline solution was introduced into the 2 mL glass vial containing the dried lipid film of **AB-C$_{12}$**. The vial was irradiated with a 365 nm LED light for 3 minutes, following which 10 μL of the 20 mM decylamine (C$_{10}$) stock solution was added. The total concentration of each component is as follows: 0.8 mM **AB-C$_{12}$**, 4 mM C$_{10}$-amine, 25 mM HEPES, and 40 mM NaCl. The resultant mixture was covered with aluminum foil and subjected to tumbling overnight (for 24 hours).

To facilitate the successive second step (formation of synthetic tissue), 2 μL of a 1 mM Dex-RhoB stock solution (equivalent to 5 mol% compared to **AB-C$_{12}$**) was added to the previous reaction mixture (50 μL). The mixture was gently agitated by vortexing for 5 seconds to ensure homogeneity. Following this, the combined mixture was exposed to a 365 nm light for 3 minutes, followed by centrifugation at 132,000 rcf for 5 minutes.

In the case of negative control experiments, the aforementioned protocol was followed with a

slight modification. Instead of applying C_{10}-amine, Dex-RhoB, or light, adjustments were made by introducing a blank solution or omitting the light irradiation step.

1.2.10. Cryo-TEM

Cryo-TEM grids (Lacey Carbon Film, Electron Microscopy Sciences #LC200-Cu) were glow-discharged using an Emitech K350 unit at 20 mA for 45 seconds. Next, 3.5 μL of a 1 mM lipid dispersion of the sample was applied to the grids. The grids were immediately vitrified using a Vitrobot Mark IV (Thermo Fisher Scientific) with 4 second blot times and a blot-force setting of 4 at 4 ºC and 100% humidity. Plunge-freezing into liquid ethane cooled by liquid nitrogen was employed for vitrification. The samples were stored in liquid nitrogen until use. Images were acquired on a Talos Arctica (FEI) operated at 200 kV, collected with a total dose of 40 e/Å^2 at 1.55 Å/pixel, and a 3 µm nominal defocus.

2. Supplementary Text

2.1. Extended technical descriptions

The following descriptions provide additional details regarding the experimental settings and data analysis.

2.1.1. Considerations for in situ reaction conditions

We opted for meta-substitution of the aldehyde group in the azobenzene moiety, because ortho or para substitution often has a negative impact on the half-life of the azobenzene isomerization, as previously described in the literature.[S3]

We observed that the presence of Cl^- ions is crucial for generating stable lipid assemblies, similar to a previously reported cationic gemini surfactant.[S4] Within the range of 0.5–1.0 mM for **AB-C$_{10}$**, concentrations of NaCl between 10 and 100 mM typically resulted in insignificant variations. However, concentrations exceeding 200 mM of NaCl resulted in the formation of much smaller vesicles and it was hard to generate giant vesicles.

The reported critical micelle concentration (CMC) of decylamine (C_{10}) is 1 mM,[S5] which aligns with our measurement (1.3 mM) using the generalized polarization method[S6,S7] under HEPES-buffered saline conditions (25 mM HEPES with 50 mM NaCl at pH 7.5). Therefore, we maintained alkylamine concentrations at 2 mM or higher. For **AB$_{CHO}$**, we consistently observed vesicle formation when the concentration was above 0.1 mM. This empirical observation implies that the CMC of **AB$_{CHO}$** is below 0.1 mM. In all cases, we employed concentrations within the range of 0.5–1.0 mM for **AB$_{CHO}$**.

In terms of the concentration of the in situ product, we endeavored to maintain it within the range of 0.5 to 1.0 mM. This range consistently yielded the most optimal quality microscopic images of lipid assemblies. When the concentration of the in situ product exceeded 2 mM, the resulting images of the assembled products exhibited excessive clutter and congestion.

2.1.2. Analysis of imine-product using LC-MS

As previously reported (*33, 34*), the reversible imine products presented difficulties in detection using LC-MS primarily due to their acid-labile properties. With the use of acid-free LC-MS solvent conditions, we were able to detect these products. Nonetheless, this led to significant tailing in the chromatogram, thereby complicating the analysis of individual components due to considerable signal overlap. For this reason, we conducted an additional reduction step on the imine products using a sodium borohydride solution. This treatment led to the formation of irreversible lipidation products via a reductive amination process.

2.1.3. Time-scales for photoisomerization processes of AB-C_{12} monomers and their assemblies

The photoisomerization process of **AB-C_{12}** monomer lipids occurs on a minute-scale in response to the corresponding LED lights, whereas macroscopic changes in **AB-C_{12}** assemblies take much longer. This discrepancy presumably reflects the period needed for the reassembly or reorganization process of pre-existing **AB-C_{12}** lipid assemblies to reach their energetically most stable states.

2.1.4. In situ formation of AB-C_{12} lipid assemblies at 37 °C

The overall outcomes, including aggregate formation and light-driven vesicle generation, were similar to those observed at room temperature. However, the increase in temperature led to the formation of less dense aggregates at 37 °C compared to those at room temperature (scale bar, 10 μm).

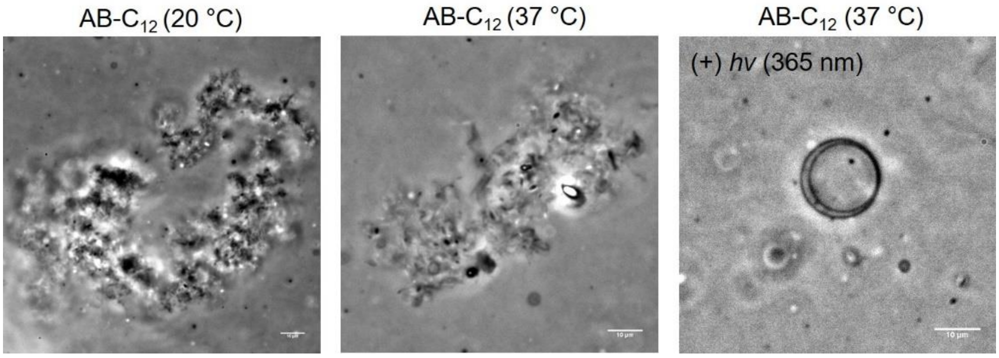

2.1.5. Influence of heat during light irradiation

Using an infrared thermometer (Omega Engineering, Inc.), we monitored the surface temperature changes of the glass vials and the slide glass while subjecting them to light activation, under the illumination setting described in section 2.2. Throughout the 5-minute irradiation period, the recorded surface temperature change remained below 1.5 °C (from 20 °C to 21.5 °C). Additionally, we confirmed that the lipid assemblies (**AB-C_{12}** aggregates and **AB-C_{10}** vesicles) did not show any noticeable morphological changes between 20 °C and 37 °C. Therefore, we can confidently conclude that the heat generated due to surface absorption of light has a negligible impact.

2.1.6. Considerations for analyzing head-group exchange using LC-DAD (diode array detector) method

The absorption spectra of the salicylic acid derivative, **SA**, undergoes a significant bathochromic shift upon the formation of the lipidation product, **SA-C10**. This absorption shift has the potential to introduce inaccuracies in the analysis of population changes (**SA** → **SA-C10**) when analyzing LC-DAD chromatograms at a specific wavelength during the head-group exchange reaction. To avoid this pitfall, we isolated two-tailed **SA-C10** product (see '2.2. Synthetic procedure' section) and compared the absorption spectra of **SA** and **SA-C10** in H_2O (20 µM). We confirmed that both compounds exhibit identical absorbance within the wavelength range of 229–232 nm (isosbestic points). Therefore, we utilized LC-DAD chromatograms at 230 nm for assessing the population change from **SA** to **SA-C12**, to determine the relative ratio between **SA** and **SA-C10** products.

2.1.7. Experimental details regarding tail exchange reaction

During the mass analysis of the tail exchange products, we found that the introduction of cetyltrimethylammonium bromide (CTAB), a non-reactive and non-reducible cationic detergent, resulted in the consistent acquisition of mass values. This phenomenon is likely due to CTAB's capability to disperse reduced lipid aggregates of **AB-C12**, thereby ensuring homogeneity within the lipid samples. Furthermore, a slight discrepancy in mass intensity was noted for identical mol% of *cis* and *trans* AB. To mitigate this effect, a uniform procedure was implemented for all LC-MS samples undergoing mass analysis. The samples were exposed to the same light conditions (1 minute under 365 nm followed by 1 minute under 470 nm) prior to LC-MS injection. This supplementary step was introduced to standardize the isomeric ratio, and we confirmed the consistency in the total summation of mass intensity between the control and light-treated conditions.

2.1.8. Detailed Information Regarding Dextran-Rhodamine B Conjugates

The reported degree of labeling (molar ratio between dextran and rhodamine B) was 2, as indicated in the vendor's analysis certificate. Experimentally measured data estimated the degree of labeling value as 1.5, which closely matched the vendor's report. Furthermore, we confirmed that Dextran-Texas Red conjugates could also yield similar light-driven crosslinking results, as illustrated below.

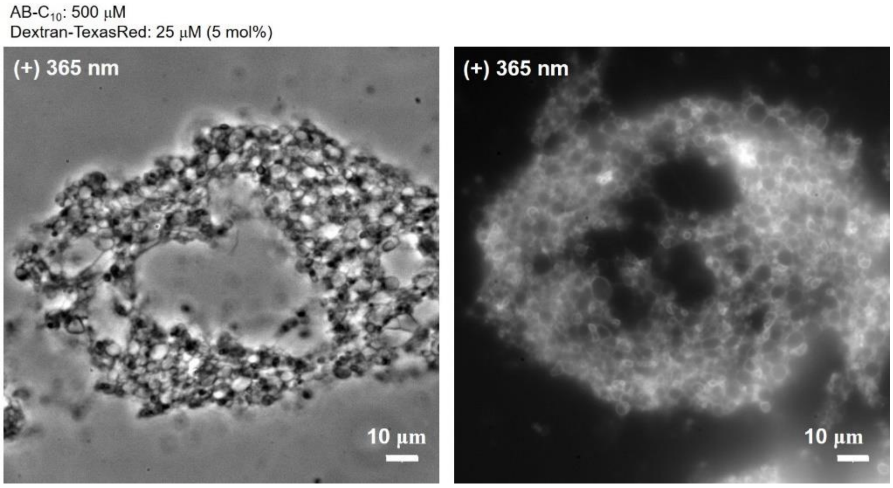

2.2. Synthetic procedure

2.2.1. Preparation of azobenzene aldehyde (AB_{CHO})

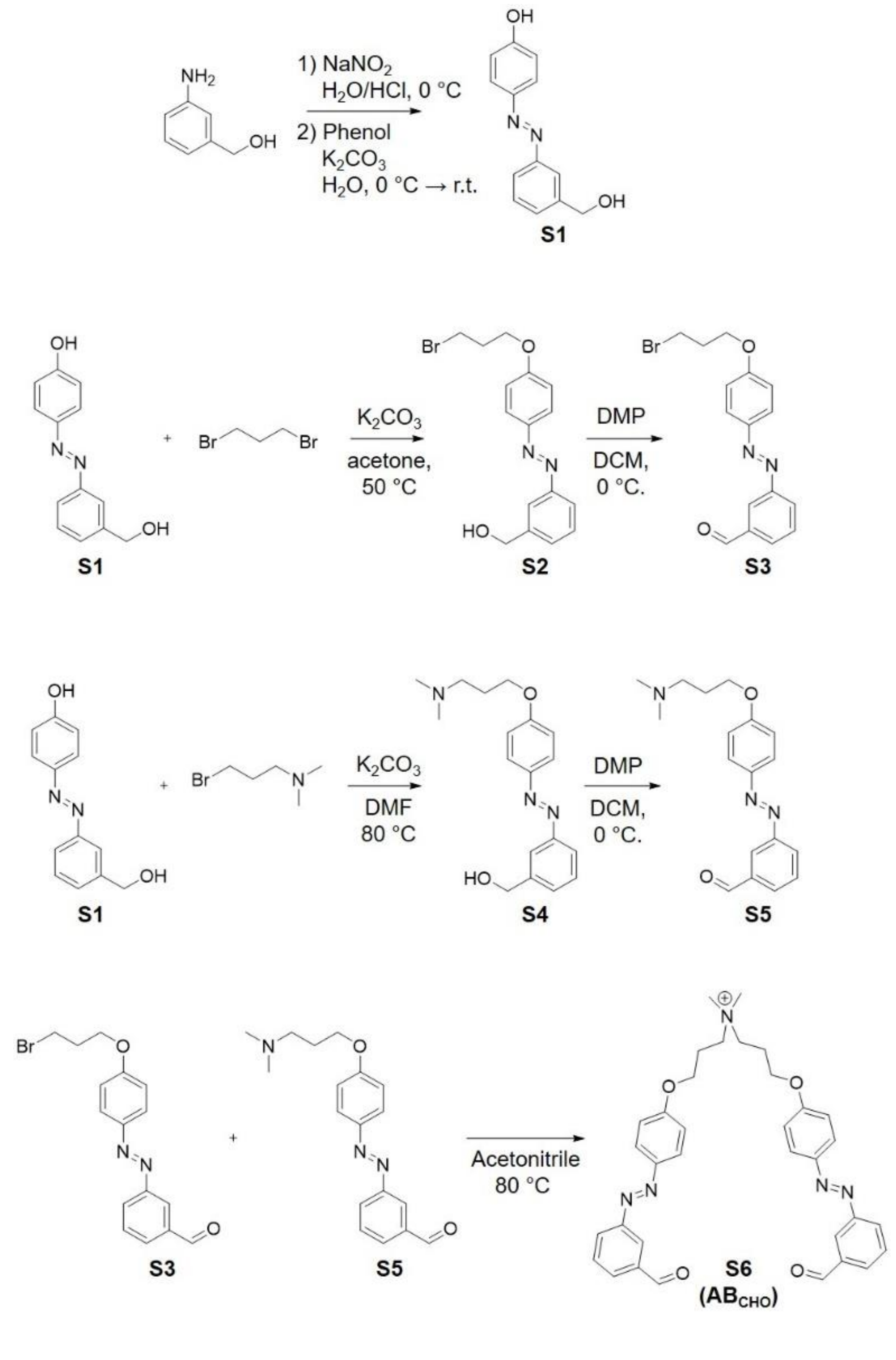

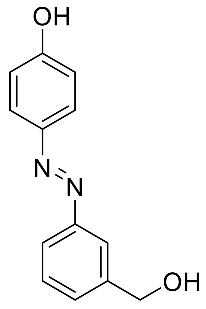

S1

Synthesis of **S1**:

A solution of 3-aminobenzyl alcohol (3.00 g, 24.4 mmol, 1 equiv.) in H_2O (40 mL) containing concentrated hydrochloric acid (36.5–38.0%, 4 mL) was cooled to 0 °C. To the cold mixture, sodium nitrite (1.76 g, 25.6 mmol, 1.05 equiv.) was added portionwise for 10 min. The mixture was stirred at 0 °C for 30 min. After that, another cold H_2O solution (40 mL) containing phenol (2.41 g, 25.6 mmol, 1.05 equiv.) and potassium carbonate (3.53 g, 25.6 mmol, 1.05 equiv.) was slowly added to the previous mixture for 10 min. The resulting mixture was stirred at 0 °C for 30 minutes and then stirred for another 30 minutes at room temperature. The crude mixture was extracted using ethyl acetate (EtOAc), followed by washing with a brine solution. The organic layer was collected, dried over sodium sulfate (Na_2SO_4), filtered, and concentrated under reduced pressure. The product was isolated by flash column chromatography (SiO_2) using an eluent of EtOAc:hexane (1:2, v/v) to yield a yellow solid of **S1** (3.57 g, Y: 64.2%).

The characterization information of **S1** is as follows:

TLC R_f 0.2 (EtOAc:hexane = 1:2).

^{1}H NMR (DMSO-d_6, 500 MHz): δ 7.80 (d, *J* = 8.6 Hz, 2H), 7.77 (s, 1H), 7.69 (d, *J* = 7.8 Hz, 1H), 7.50 (t, *J* = 7.7 Hz, 1H), 7.43 (d, *J* = 7.3 Hz, 1H), 6.94 (d, *J* = 8.8 Hz, 2H), 5.42 (brs., 1H), 4.59 (s, 2H).

^{13}C NMR (DMSO-d_6, 126 MHz): δ 161.1, 152.2, 145.3, 144.1, 129.3, 128.6, 125.0, 121.5, 119.3, 116.1, 62.7.

HRMS (ESI): m/z calcd for $C_{13}H_{13}N_2O_2^+$ $[M+H]^+$: 229.0972, found 229.0975.

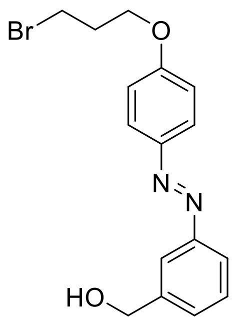

S2

Synthesis of **S2**:

To a solution of **S1** (298 mg, 1.31 mmol, 1 equiv.) in acetone (10 mL) were added 1,3-dibromopropane (2.64 g, 13.1 mmol, 10 equiv.) and potassium carbonate (1.81 g, 13.1 mmol, 10 equiv.). The mixture was stirred at 50 °C for 20 hours. The resulting solution was washed with brine and extracted using EtOAc. The collected organic portion was dried over Na_2SO_4, filtered, and concentrated under reduced pressure. The crude product was purified by flash column chromatography (SiO_2) using an eluent of EtOAc:hexane (1:3, v/v) to yield **S2** as an orange solid (385 mg, Y: 84.2%).

The characterization information of **S2** is as follows:

TLC $\mathbf{R_f}$ 0.3 (EtOAc:hexane = 1:2).

^{1}**H NMR** (chloroform-*d*, 500 MHz): δ 7.93 (d, *J* = 9.0 Hz, 2H), 7.88 (s, 1H), 7.82 (dt, *J* = 7.8, 1.7 Hz, 1H), 7.51 (t, *J* = 7.6 Hz, 1H), 7.46 (d, *J* = 7.6 Hz, 1H), 7.02 (d, *J* = 8.8 Hz, 2H), 4.81 (s, 2H), 4.20 (t, *J* = 5.9 Hz, 2H), 3.64 (t, *J* = 6.4 Hz, 2H), 2.37 (qui, *J* = 6.1 Hz, 2H).

^{13}**C NMR** (chloroform-*d*, 126 MHz): δ 161.3, 153.0, 147.1, 142.1, 129.4, 128.9, 125.0, 122.6, 120.4, 114.9, 65.7, 65.2, 32.3, 30.0.

HRMS (ESI): m/z calcd for $C_{16}H_{18}BrN_2O_2^+$ $[M+H]^+$: 349.0546, found 349.0545.

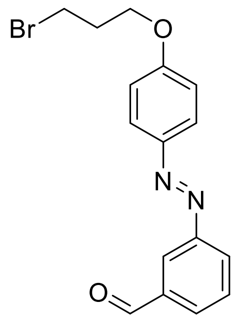

S3

Synthesis of **S3**:

To a dichloromethane solution (DCM, 10 mL) of **S2** (350 mg, 1.00 mmol, 1 equiv.), which was cooled to 0 °C, was added Dess–Martin periodinane (637 mg, 1.50 mmol, 1.5 equiv.) portionwise for 5 min. The resulting solution was stirred at 0 °C for 1 hour. The crude mixture was purified by flash column chromatography (SiO_2) using an eluent of EtOAc:hexane (1:10, v/v) to yield **S3** as an orange solid (257 mg. Y: 74.0%).

The characterization information of **S3** is as follows:

TLC R_f 0.25 (EtOAc:hexane = 1:10).

^{1}H NMR (chloroform-*d*, 500 MHz): δ 10.13 (s, 1H), 8.37 (t, *J* = 1.8 Hz, 1H), 8.10–8.21 (m, 1H), 7.90 –8.04 (m, 3H), 7.68 (t, *J* = 7.7 Hz, 1H), 6.98–7.11 (m, 2H), 4.22 (t, *J* = 5.9 Hz, 2H), 3.64 (t, *J* = 6.4 Hz, 2H), 2.38 (qui, *J* = 6.1 Hz, 2H).

^{13}C NMR (chloroform-*d*, 126 MHz): δ 192.1, 161.8, 153.2, 146.9, 137.4, 130.7, 129.9, 128.8, 125.3, 123.7, 114.9, 65.7, 32.3, 30.0.

HRMS (ESI): m/z calcd for $C_{16}H_{16}BrN_2O_2^+$ $[M+H]^+$: 347.0390, found 347.0392.

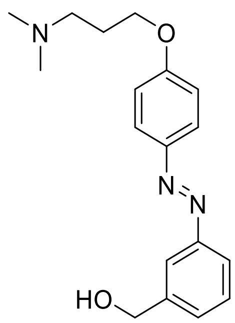

S4

Synthesis of **S4**:

To a solution of **S1** (318 mg, 1.39 mmol, 1 equiv.) in dimethylformamide (DMF, 7 mL) were added 3-dimethylamino-1-propyl chloride hydrochloride (440 mg, 2.79 mmol, 2 equiv.) and potassium carbonate (963 mg, 6.97 mmol, 5 equiv.). The mixture was stirred at 80 °C for 24 hours. The resulting solution was washed with H_2O and then extracted using DCM. The collected organic portion was dried over Na_2SO_4, filtered, and concentrated under reduced pressure. The crude product was purified by flash column chromatography (SiO_2) using an eluent of DCM:methanol (10:1, v/v) to yield **S4** as an orange solid (187 mg, Y: 42.7%).

The characterization information of **S4** is as follows:

TLC R_f 0.15 (DCM:methanol = 10:1).

^{1}H NMR (chloroform-*d*, 300 MHz): δ 7.89 (d, *J* = 9.0 Hz, 3H), 7.80 (dt, *J* = 7.3, 1.9 Hz, 1H), 7.39–7.57 (m, 2H), 6.98 (d, *J* = 9.2 Hz, 2H), 4.80 (s, 2H), 4.11 (t, *J* = 6.1 Hz, 2H), 2.75 (t, *J* = 7.5 Hz, 2H), 2.48 (s, 6H), 2.07–2.23 (m, 2H).

^{13}C NMR (chloroform-*d*, 126 MHz): δ 161.3, 153.0, 147.1, 142.3, 129.4, 129.0, 124.9, 122.5, 120.4, 114.7, 66.0, 65.0, 56.2, 44.7, 26.5.

HRMS (ESI): m/z calcd for $C_{18}H_{24}N_3O_2^+$ $[M+H]^+$: 314.1863, found 314.1860.

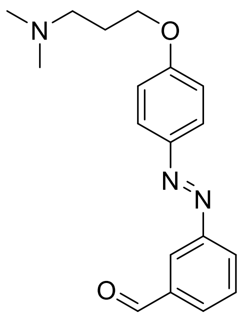

S5

Synthesis of **S5**:

To a cold DCM solution (2 mL) containing **S4** (100 mg, 0.32 mmol, 1 equiv.) at 0 °C was added Dess–Martin periodinane (162 mg, 0.38 mmol, 1.2 equiv.) portionwise for 5 min. The resulting solution was stirred at 0 °C for 1 hour. The crude mixture was purified by flash column chromatography (SiO_2) using an eluent of DCM:methanol (10:1, v/v) to yield **S5** as a red waxy solid (66 mg. Y: 66.4%).

The characterization information of **S5** is as follows:

TLC R_f 0.3 (DCM:methanol = 10:1).

^{1}H NMR (chloroform-*d*, 500 MHz): δ 10.13 (s, 1H), 8.37 (t, J = 1.8 Hz, 1H), 8.12–8.18 (m, 1H), 7.89–8.03 (m, 3H), 7.69 (t, J = 7.7 Hz, 1H), 7.01 (d, J = 9.0 Hz, 2H), 4.21 (t, J = 5.5 Hz, 2H), 3.23–3.37 (m, 2H), 2.81–2.98 (m, 6H), 2.41–2.55 (m, 2H).

^{13}C NMR (chloroform-*d*, 126 MHz): δ 192.1, 161.1, 153.1, 147.2, 137.4, 130.9, 130.0, 128.8, 125.3, 123.7, 114.8, 65.0, 56.0, 43.3, 24.7.

HRMS (ESI): m/z calcd for $C_{18}H_{22}N_3O_2^+$ $[M+H]^+$: 312.1707, found 312.1708.

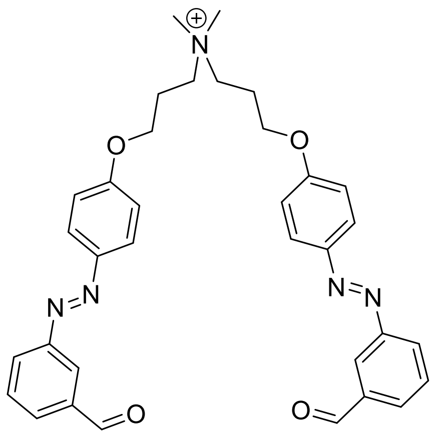

S6

Synthesis of **S6**:

A solution of **S5** (54.2 mg, 0.17 mmol, 1 equiv.) and **S3** (90.7 mg, 0.26 mmol, 1.5 equiv.) in acetonitrile (1.8 mL) was stirred at 80 °C overnight (>20 h). The resulting solution was purified by flash column chromatography (SiO_2) using an eluent of DCM:methanol (20:1→10:1, v/v) to yield **S6** as an orange solid. Further treatments, including dissolving in saturated brine solution, extracting with DCM, drying over Na_2SO_4, and concentration under reduced pressure, were conducted. Lastly, recrystallization by heating the suspension of **S6** in acetone, followed by cooling and concentrating under room temperature, was performed to afford the chloride salts of **S6** as an orange solid (51.2 mg, Y: 47.9%).

The characterization information of **S6** is as follows:

TLC R_f 0.1 (DCM:methanol = 10:1).

^{1}H NMR (chloroform-*d*, 500 MHz): δ 10.11 (s, 2H), 8.34 (s, 2H), 8.12 (d, *J* = 8.3 Hz, 2H), 7.96 (d, *J* = 7.6 Hz, 2H), 7.93 (d, *J* = 9.0 Hz, 4H), 7.67 (t, *J* = 7.7 Hz, 2H), 7.01 (d, *J* = 9.0 Hz, 4H), 4.26 (t, *J* = 5.4 Hz, 4H), 3.87–4.08 (m, 4H), 3.53 (s, 6H), 2.41 (d, *J* = 11.5 Hz, 4H).

^{13}C NMR (chloroform-*d*, 126 MHz): δ 192.0, 160.8, 153.0, 147.3, 137.4, 131.0, 130.0, 128.8, 125.3, 123.6, 114.9, 64.5, 62.0, 51.8, 23.3.

HRMS (ESI): m/z calcd for $C_{34}H_{36}N_5O_4^+$ $[M]^+$: 578.2762, found 578.2769.

2.2.2. Preparation of salicylaldehyde derivative (SA)

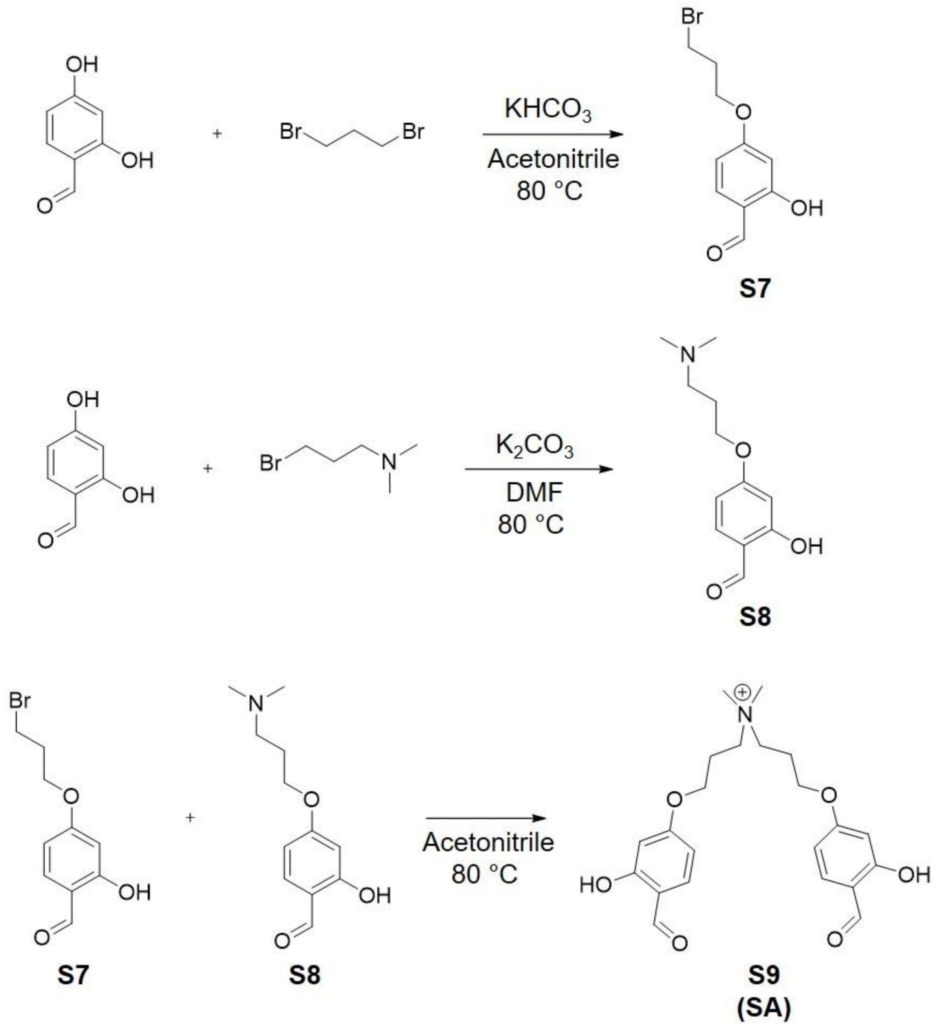

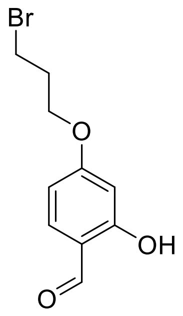

S7

Synthesis of **S7**:

To a solution of 2,4-dihydroxybenzaldehyde (201 mg, 1.46 mmol, 1 equiv.) in acetonitrile (8 mL) were added 1,3-dibromopropane (881 mg, 4.37 mmol, 3 equiv.) and potassium bicarbonate (437 mg, 4.37 mmol, 3 equiv.). The mixture was stirred at 80 °C for 4 hours. The resulting solution was washed with H_2O, extracted using EtOAc, dried over Na_2SO_4, and concentrated under reduced pressure. The crude mixture was purified by flash column chromatography (SiO_2) using an eluent of EtOAc:hexane (1:4, v/v) to yield **S7** as a white solid (190.4 mg, Y: 50.5%).

The characterization information of **S7** is as follows:

TLC R_f 0.5 (EtOAc:hexane = 1:4)

^{1}H NMR (chloroform-*d*, 300 MHz): δ 11.47 (s, 1H), 9.72 (s, 1H), 7.44 (d, J = 8.6 Hz, 1H), 6.54 (dd, J = 8.6, 2.4 Hz, 1H), 6.44 (d, J = 2.4 Hz, 1H), 4.17 (t, J = 5.8 Hz, 2H), 3.59 (t, J = 6.3 Hz, 2H), 2.34 (qui, J = 6.1 Hz, 2H).

Compound **S7** was previously reported. The ^{1}H NMR spectrum was found in accordance with the previously reported synthesis.[S8]

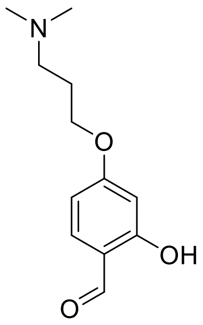

S8

Synthesis of **S8**:

A DMF (8 mL) solution containing 2,4-dihydroxybenzaldehyde (500 mg, 3.62 mmol, 1 equiv.), 3-dimethylamino-1-propyl chloride hydrochloride (1144 mg, 7.24 mmol, 2 equiv.), and potassium carbonate (1500 mg, 10.9 mmol, 3 equiv.) was stirred at 80 °C overnight (>20 h). The resulting solution was washed with brine followed by extraction using EtOAc. The crude mixture was purified by flash column chromatography (SiO_2) using an eluent of DCM:methanol (20:1→10:1, v/v) to yield **S8** as a brown oil (122 mg, Y: 15.1%).

The characterization information of **S8** is as follows:

TLC R_f 0.3 (DCM:methanol = 10:1).

^{1}H NMR (chloroform-*d*, 500 MHz): *δ* 11.46 (brs, 1H), 9.72 (s, 1H), 7.44 (d, *J* = 8.8 Hz, 1H), 6.52 (dd, *J* = 8.7, 2.3 Hz, 1H), 6.41 (d, *J* = 2.2 Hz, 1H), 4.11 (t, *J* = 6.0 Hz, 2H), 2.81 (t, *J* = 7.5 Hz, 2H), 2.54 (s, 6H), 2.12–2.28 (m, 2H).

^{13}C NMR (chloroform-*d*, 126 MHz): *δ* 194.6, 165.8, 164.5, 135.5, 115.4, 108.5, 101.4, 66.1, 56.0, 44.5, 26.0.

HRMS (ESI): m/z calcd for $C_{12}H_{18}NO_3^+$ $[M+H]^+$: 224.1281, found 224.1280.

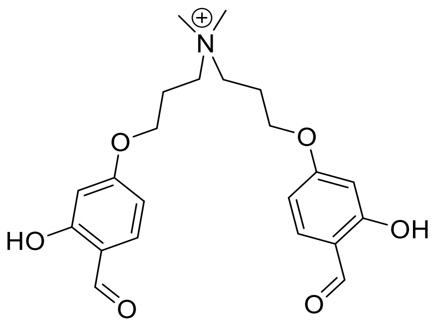

S9 (SA)

Synthesis of **S9**:

The solution of **S8** (59 mg, 0.26 mmol, 1 equiv.) and **S7** (103 mg, 0.40 mmol, 1.5 equiv.) in acetonitrile (2.6 mL) was stirred at 80 °C overnight (>20 h). The crude mixture was purified by flash column chromatography (SiO_2) using an eluent of DCM:methanol (10:1, v/v) to yield the bromide salts of **S9** as a white solid (95 mg, Y: 74.5%).

The characterization information of **S9** is as follows:

TLC R_f 0.25 (DCM:methanol = 10:1).

^{1}H NMR (methanol-d_4, 500 MHz): δ 9.80 (s, 2H), 7.61 (d, J = 8.8 Hz, 2H), 6.63 (dd, J = 8.7, 2.3 Hz, 2H), 6.50 (d, J = 2.2 Hz, 2H), 4.20 (t, J = 5.6 Hz, 4H), 3.54–3.69 (m, 4H), 3.22 (s, 6H), 2.27–2.39 (m, 4H).

^{13}C NMR (methanol-d_4, 126 MHz): δ 195.8, 166.8, 165.3, 136.3, 117.2, 109.1, 102.3, 66.1, 62.5, 51.7, 23.7.

HRMS (ESI): m/z calcd for $C_{22}H_{28}NO_6^+$ $[M]^+$: 402.1911, found 402.1915.

2.2.3. Preparation of isolated imine-products (AB-C_{12}, AB-C_{10}, and SA-C_{12})

Two-tailed imine products were isolated using a modified synthetic procedure based on a previous report.[S9]

General procedure:

To an ethanol solution of an aldehyde derivative (1 equiv.), an alkylamine (1.5–2 equiv. compared to the number of aldehyde group) and a catalytic amount of acetic acid (10 mol%) were added. The mixture was stirred under reflux conditions overnight (>20 h). The reaction mixture was cooled to room temperature and then concentrated under reduced pressure. The resulting residue was washed with a small amount of acetone 2–3 times and collected to yield two-tailed imine products. These products were characterized using proton and carbon NMR spectroscopy, as well as high-resolution mass spectrometry (HRMS).

Synthesis of **AB-C_{12}**:

Under the general procedure, **S6** (14.6 mg, 0.024 mmol) and dodecylamine (17.6 mg, 0.095 mmol) were used to yield the chloride salts of **AB-C_{12}** as an orange solid (14.7 mg, Y: 65.2%).

^{1}H NMR (chloroform-*d*, 500 MHz): δ 8.33 (s, 2H), 8.16 (s, 2H), 7.89 (d, J = 8.6 Hz, 4H), 7.83 (d, J = 7.6 Hz, 2H), 7.51 (t, J = 7.8 Hz, 2H), 6.99 (d, J = 8.8 Hz, 4H), 4.21 (t, J = 5.6 Hz, 4H), 3.76–3.94 (m, 4H), 3.62 (t, J = 7.1 Hz, 4H), 3.50 (s, 6H), 2.30–2.42 (m, 4H), 1.70 (qui, J = 7.2 Hz, 4H), 1.14–1.38 (m, 36H), 0.86 (t, J = 7.0 Hz, 6H).

^{13}C NMR (chloroform-*d*, 126 MHz): δ 160.5, 160.3, 152.9, 147.4, 137.5, 129.7, 129.4, 125.1, 125.0, 122.1, 114.9, 64.5, 62.0, 61.8, 51.8, 32.1, 31.0, 29.82, 29.78*, 29.75, 29.6, 29.5, 27.5, 23.2, 22.8, 14.3. (*overlapped)

HRMS (ESI): m/z calcd for $C_{58}H_{86}N_7O_2^+$ $[M]^+$: 912.6838, found 912.6840.

Synthesis of **AB-C_{10}**:

Under the general procedure, **S6** (13.6 mg, 0.022 mmol) and decylamine (13.9 mg, 0.089 mmol) were used to yield the chloride salts of **AB-C_{10}** as an orange solid (12.0 mg, Y: 60.7%).

^{1}H NMR (chloroform-*d*, 500 MHz): δ 8.34 (s, 2H), 8.16 (s, 2H), 7.90 (d, J = 9.0 Hz, 6H), 7.83 (d, J = 7.6 Hz, 2H), 7.52 (t, J = 7.7 Hz, 2H), 6.99 (d, J = 9.0 Hz, 4H), 4.21 (t, J = 5.3 Hz, 4H), 3.82–3.97 (m, 4H), 3.63 (t, J = 7.0 Hz, 4H), 3.51 (s, 6H), 2.30–2.44 (m, 4H), 1.70 (qui, J = 7.0 Hz, 4H), 1.18–1.41 (m, 28H), 0.86 (t, J = 7.0 Hz, 6H).

^{13}C NMR (chloroform-*d*, 126 MHz): δ 160.5, 160.2, 152.9, 147.4, 137.4, 129.7, 129.5, 125.1, 125.0, 122.1, 114.8, 64.5, 62.0, 61.7, 51.8, 32.0, 31.0, 29.75, 29.74, 29.6, 29.5, 27.5, 23.2, 22.8, 14.3.

HRMS (ESI): m/z calcd for $C_{54}H_{78}N_7O_2^+$ $[M]^+$: 856.6212, found 856.6220.

Synthesis of **SA-C_{12}**:

Under the general procedure, **S9** (16.7 mg, 0.035 mmol) and dodecylamine (19.3 mg, 0.104 mmol) were used to yield the bromide salts of **SA-C_{12}** as a white solid (19.7 mg, Y: 69.7%).

^{1}H NMR (methanol-d_4, 500 MHz): δ 8.18 (s, 2H), 7.13 (d, J = 8.8 Hz, 2H), 6.22 (dd, J = 8.8, 2.4 Hz, 2H), 6.16 (d, J = 2.2 Hz, 2H), 4.10 (t, J = 5.6 Hz, 4H), 3.49–3.65 (m, 8H), 3.19 (s, 6H), 2.22– 2.36 (m, 4H), 1.62–1.77 (m, 4H), 1.24–1.47 (m, 36H), 0.90 (t, J = 7.0 Hz, 6H).

^{13}C NMR (methanol-d_4, 126 MHz): δ 178.0, 167.1, 165.5, 136.2, 112.1, 107.4, 104.0, 65.4, 62.4, 52.8, 51.7, 33.1, 31.4, 30.80, 30.79, 30.71, 30.67, 30.5, 30.3, 27.7, 23.8, 23.6, 14.5

HRMS (ESI): m/z calcd for $C_{46}H_{78}N_3O_4^+$ $[M]^+$: 736.5987, found 736.5999.

3. Appendix: NMR spectra

S1

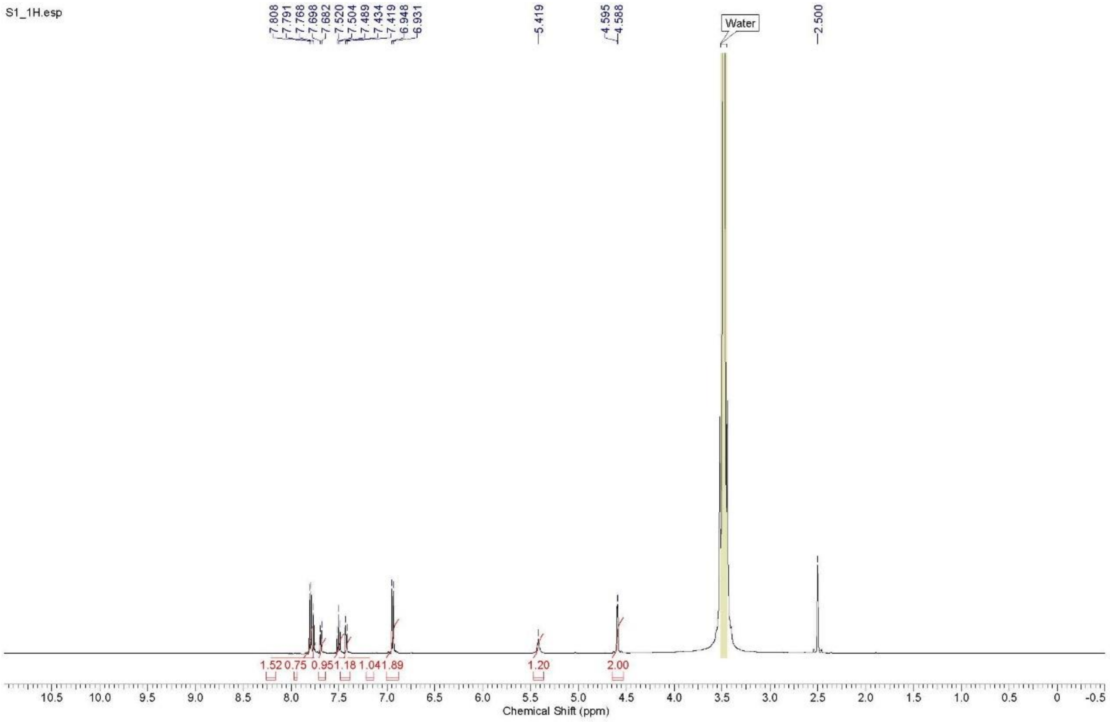

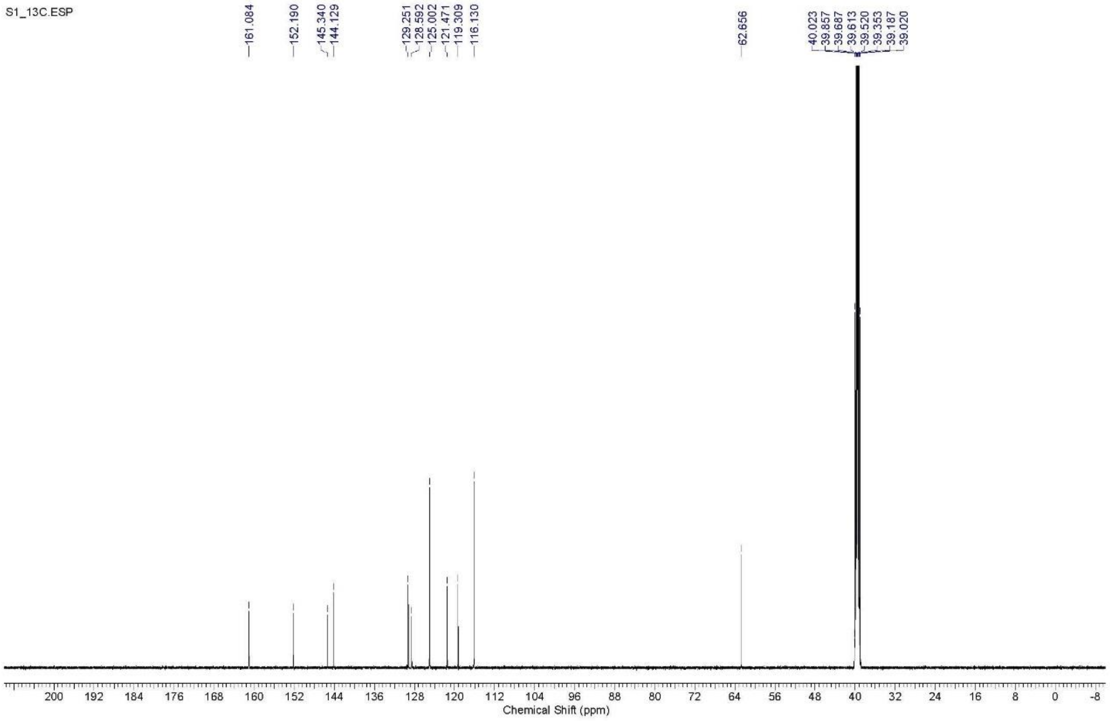

S2

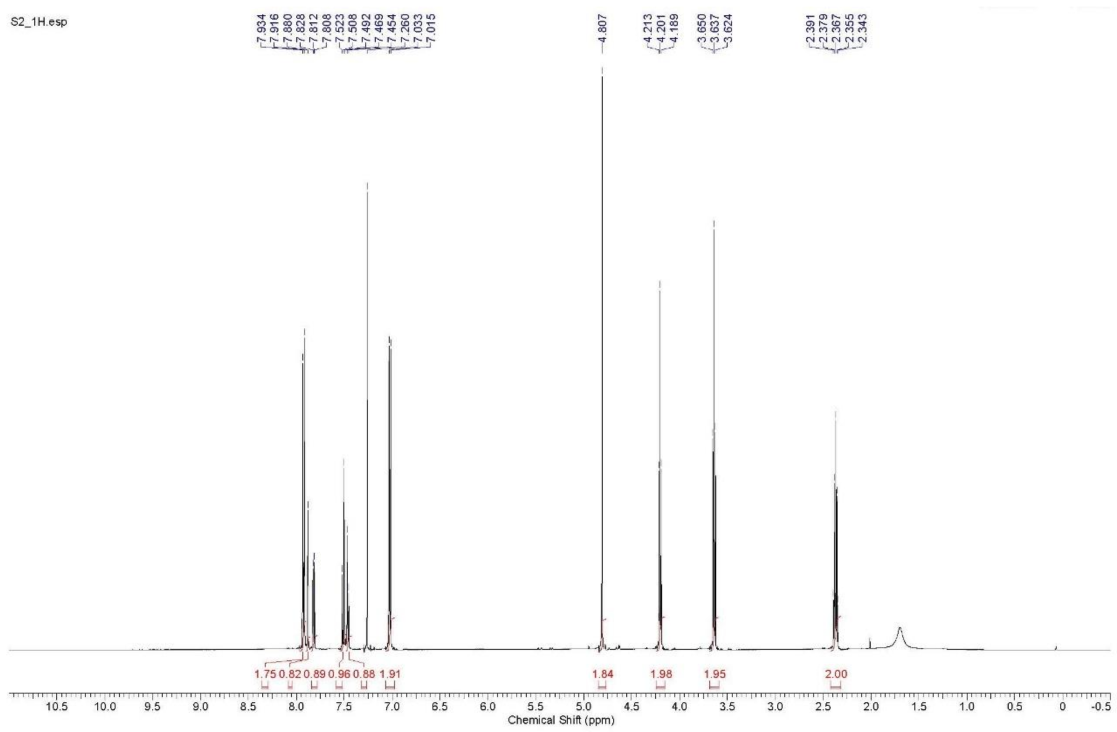
S2_1H.esp
7.934
7.916
7.880
7.828
7.812
7.808
7.523
7.508
7.492
7.469
7.454
7.260
7.033
7.015
4.807
4.213
4.201
4.189
3.650
3.637
3.624
2.391
2.379
2.367
2.355
2.343
1.75
0.82
0.89
0.96
0.88
1.91
1.84
1.98
1.95
2.00
Chemical Shift (ppm)

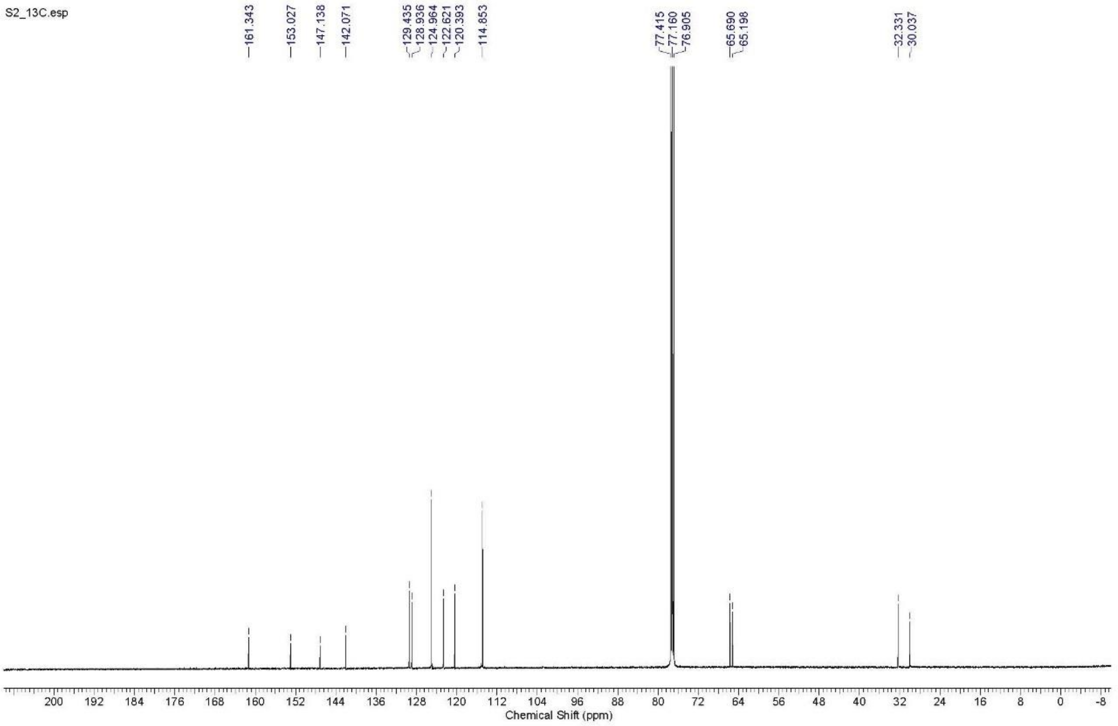
S2_13C.esp
161.343
153.027
147.138
142.071
129.435
128.936
124.964
122.621
120.393
114.853
77.415
77.160
76.905
65.690
65.198
32.331
30.037
Chemical Shift (ppm)

S3

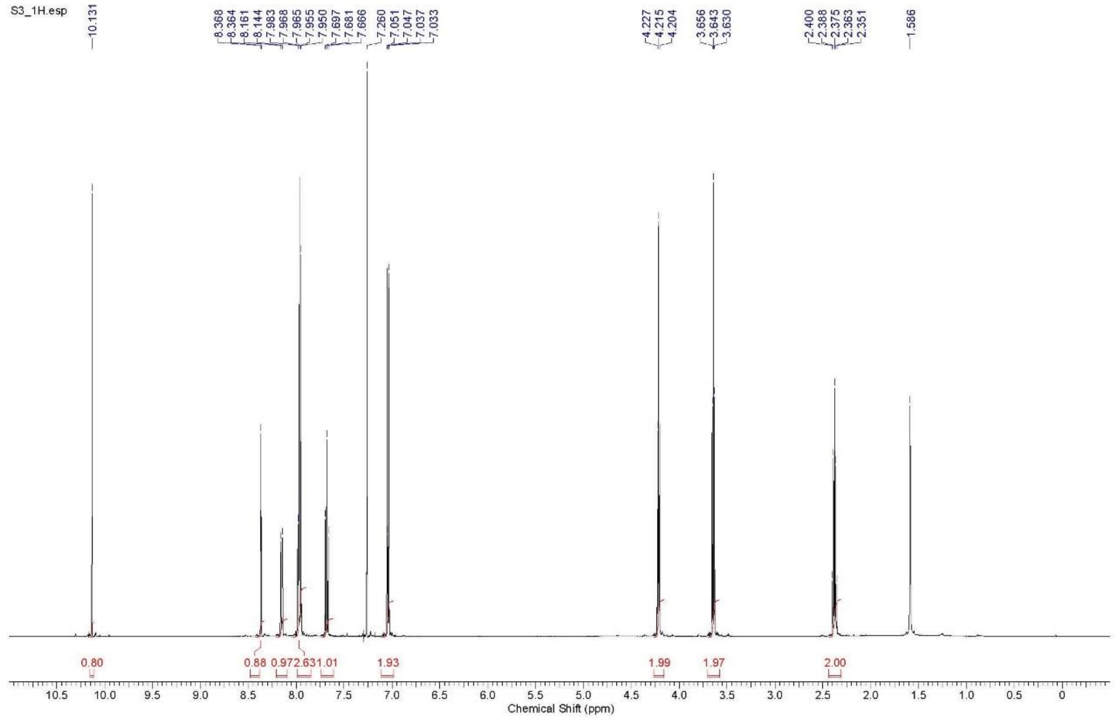
S3_1H.esp
10.131
8.368
8.364
8.161
8.144
7.983
7.968
7.965
7.955
7.950
7.697
7.681
7.666
7.260
7.051
7.047
7.037
7.033
4.227
4.215
4.204
3.656
3.643
3.630
2.400
2.388
2.375
2.363
2.351
1.586
0.80
0.88
0.97
2.63
1.01
1.93
1.99
1.97
2.00
Chemical Shift (ppm)

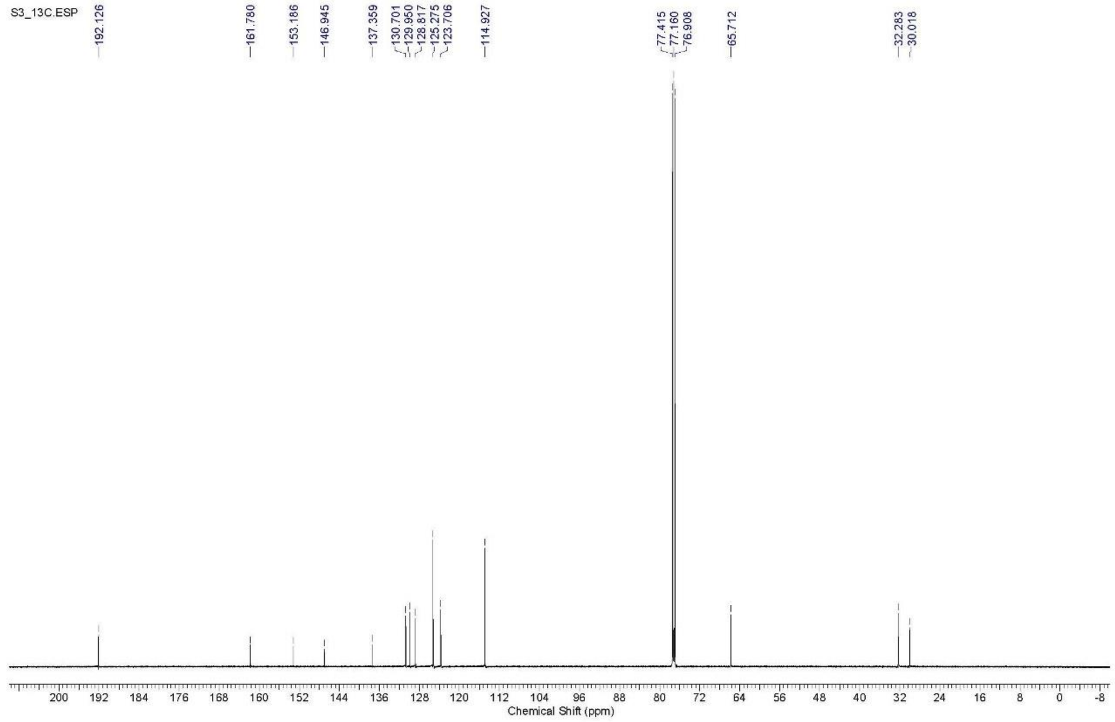
S3_13C.ESP
192.126
161.780
153.186
146.945
137.359
130.701
129.950
128.817
125.275
123.706
114.927
77.415
77.160
76.908
65.712
32.283
30.018
Chemical Shift (ppm)

S4

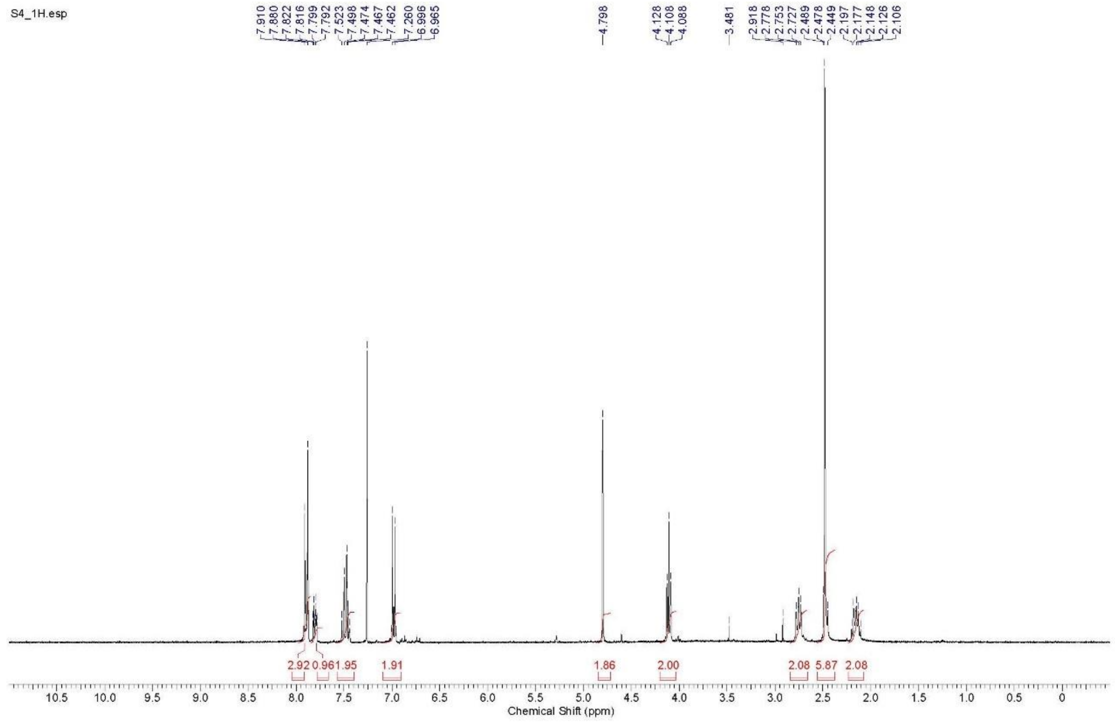

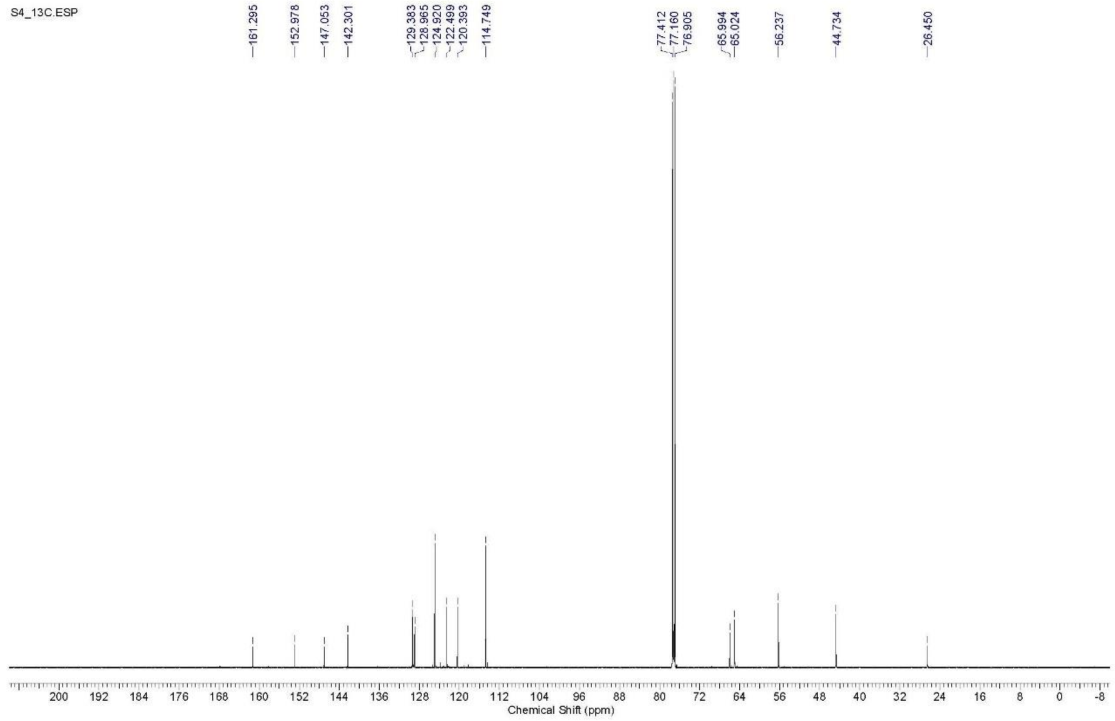

S5

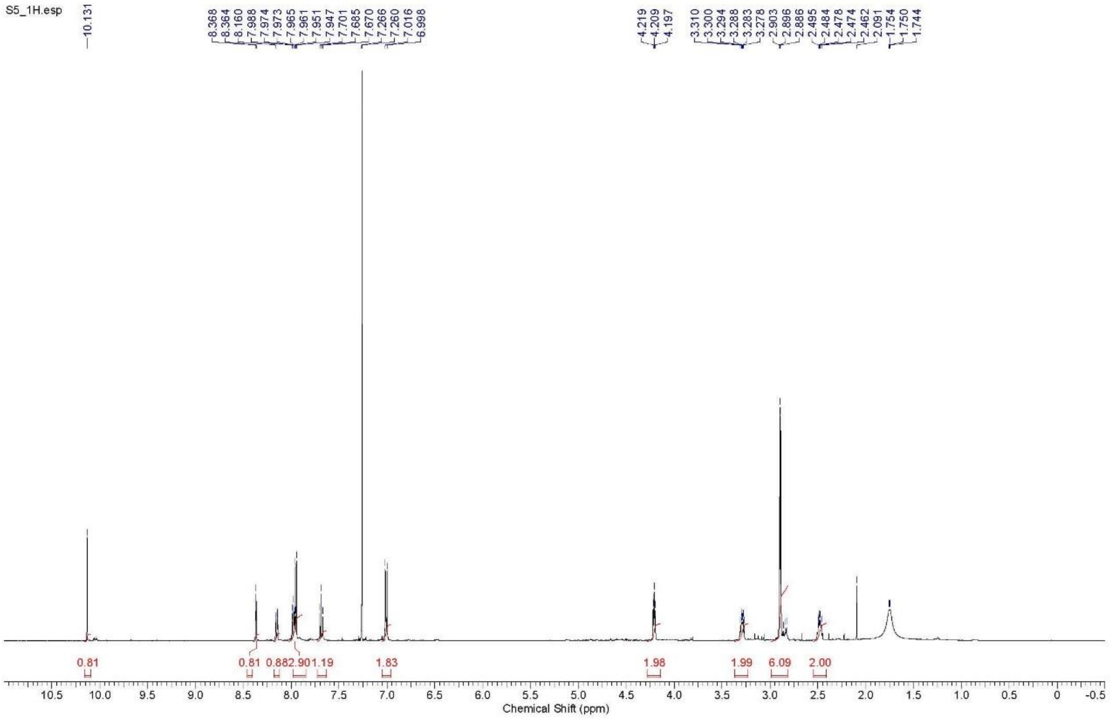
S5_1H.esp
10.131
8.368
8.364
8.160
7.988
7.974
7.973
7.965
7.961
7.951
7.947
7.701
7.685
7.670
7.266
7.260
7.016
6.998
4.219
4.209
4.197
3.310
3.300
3.294
3.288
3.283
3.278
2.903
2.896
2.886
2.495
2.484
2.478
2.474
2.462
2.091
1.754
1.750
1.744
0.81
0.81
0.88
2.90
1.19
1.83
1.98
1.99
6.09
2.00
Chemical Shift (ppm)

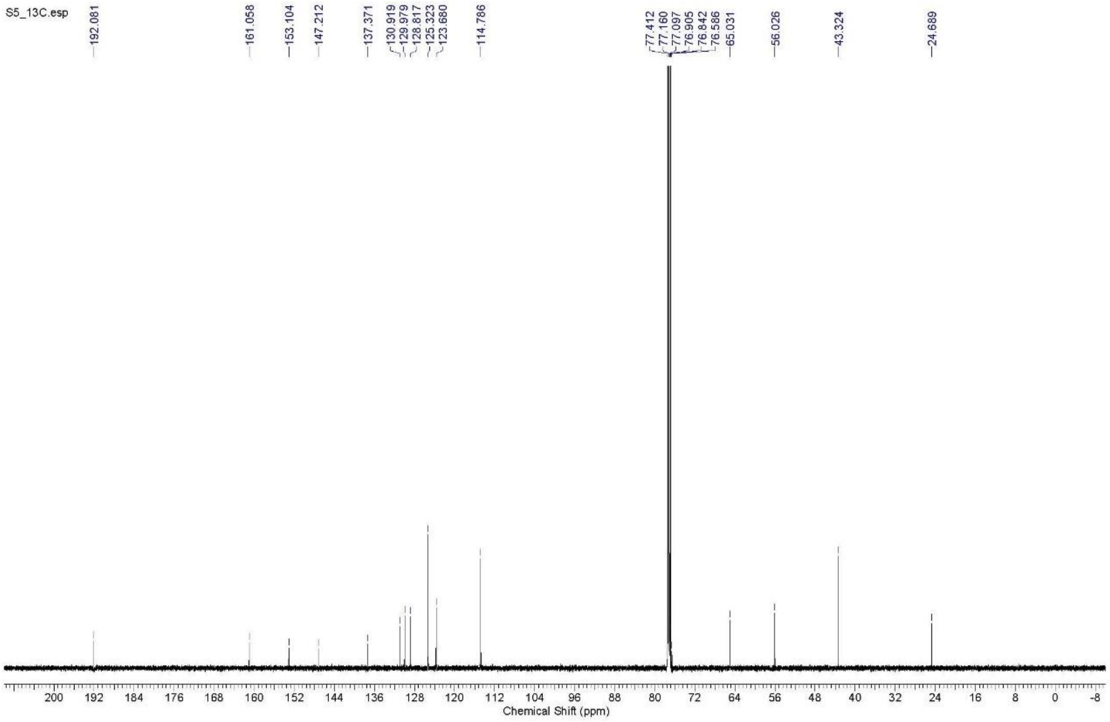
S5_13C.esp
192.081
161.058
153.104
147.212
137.371
130.919
129.979
128.817
125.323
123.680
114.786
77.412
77.160
77.097
76.905
76.842
76.586
65.031
56.026
43.324
24.689
Chemical Shift (ppm)

S6

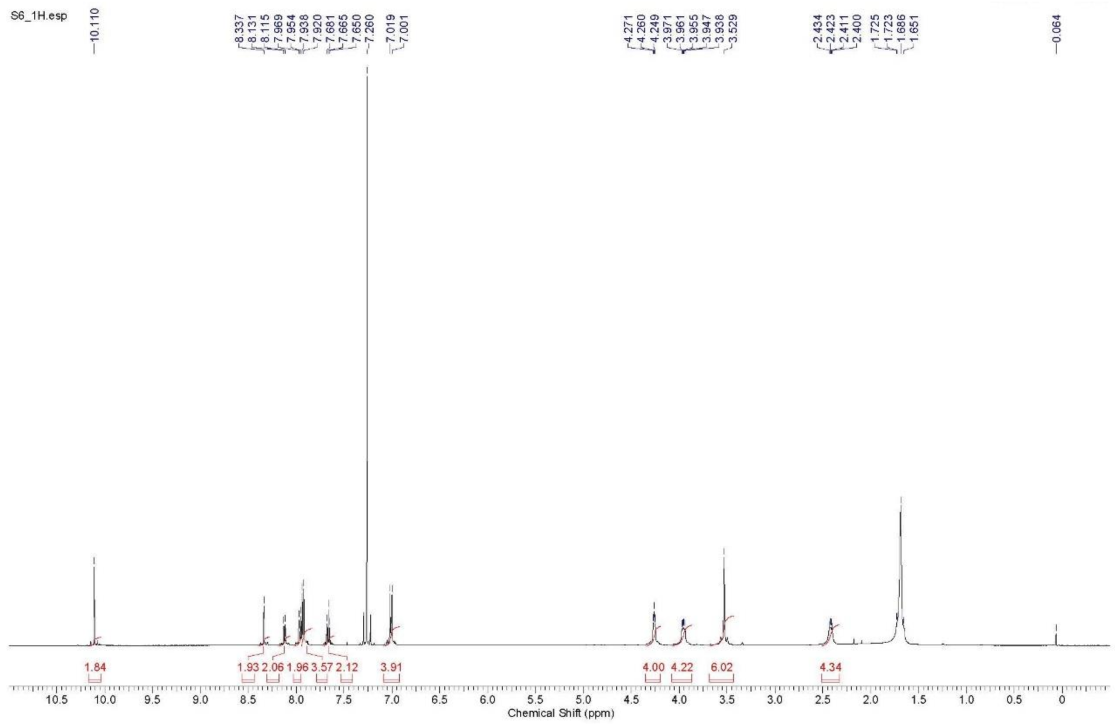
S6_1H.esp
10.110
8.337
8.131
8.115
7.969
7.954
7.938
7.920
7.681
7.665
7.650
7.260
7.019
7.001
4.271
4.260
4.249
3.971
3.961
3.955
3.947
3.938
3.529
2.434
2.423
2.411
2.400
1.725
1.723
1.686
1.651
0.064
1.84
1.93 2.06 1.96 3.57 2.12
3.91
4.00 4.22
6.02
4.34
10.5 10.0 9.5 9.0 8.5 8.0 7.5 7.0 6.5 6.0 5.5 5.0 4.5 4.0 3.5 3.0 2.5 2.0 1.5 1.0 0.5 0
Chemical Shift (ppm)

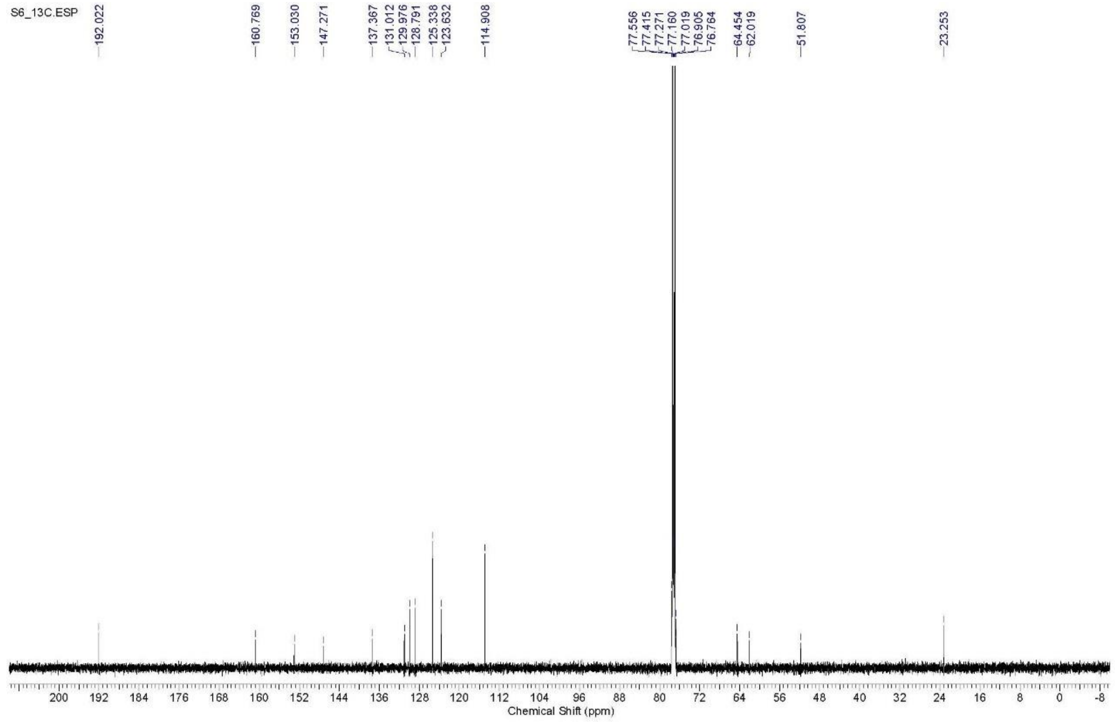
S6_13C.ESP
192.022
160.769
153.030
147.271
137.367
131.012
129.976
128.791
125.338
123.632
114.908
77.556
77.415
77.271
77.160
77.019
76.905
76.764
64.454
62.019
51.807
23.253
200 192 184 176 168 160 152 144 136 128 120 112 104 96 88 80 72 64 56 48 40 32 24 16 8 0 -8
Chemical Shift (ppm)

S7

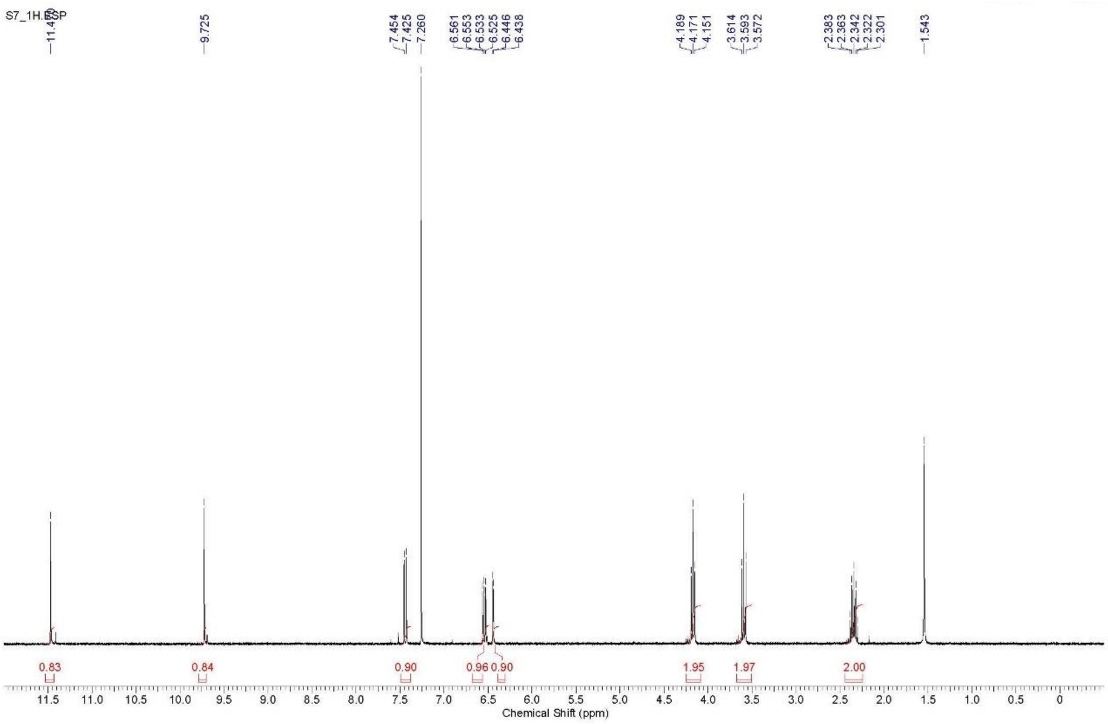
S7_1H.ESP
9.725
7.454
7.425
7.260
6.561
6.553
6.533
6.525
6.446
6.438
4.189
4.171
4.151
3.614
3.593
3.572
2.383
2.363
2.342
2.322
2.301
1.543
0.83
0.84
0.90
0.96
0.90
1.95
1.97
2.00
Chemical Shift (ppm)

S8

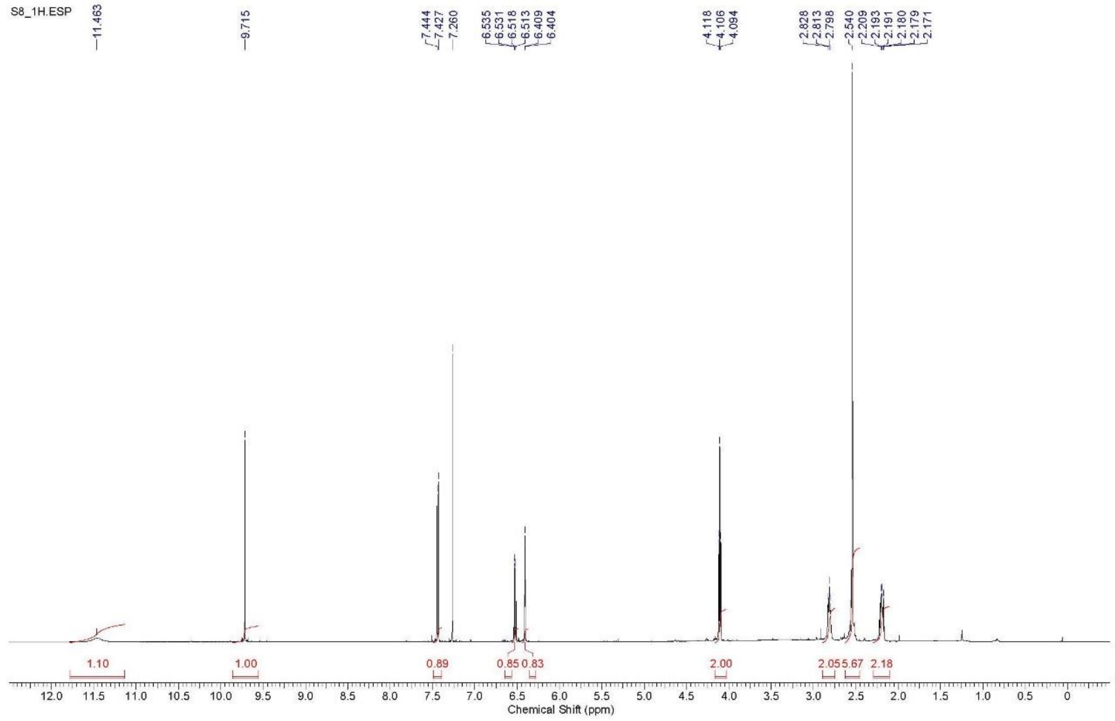
S8_1H.ESP
11.463
9.715
7.444
7.427
7.260
6.535
6.531
6.518
6.513
6.409
6.404
4.118
4.106
4.094
2.828
2.813
2.798
2.540
2.209
2.193
2.191
2.180
2.179
2.171
1.10
1.00
0.89
0.85
0.83
2.00
2.05
5.67
2.18
12.0 11.5 11.0 10.5 10.0 9.5 9.0 8.5 8.0 7.5 7.0 6.5 6.0 5.5 5.0 4.5 4.0 3.5 3.0 2.5 2.0 1.5 1.0 0.5 0
Chemical Shift (ppm)

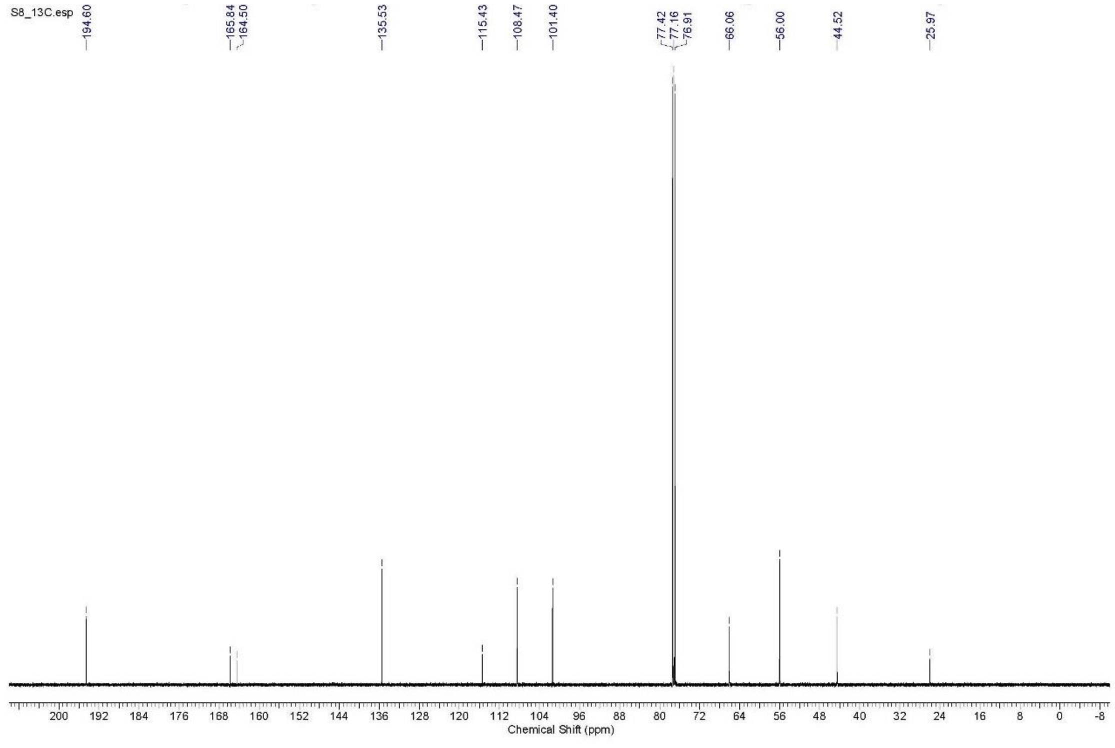
S8_13C.esp
194.60
165.84
164.50
135.53
115.43
108.47
101.40
77.42
77.16
76.91
66.06
56.00
44.52
25.97
200 192 184 176 168 160 152 144 136 128 120 112 104 96 88 80 72 64 56 48 40 32 24 16 8 0 -8
Chemical Shift (ppm)

S9

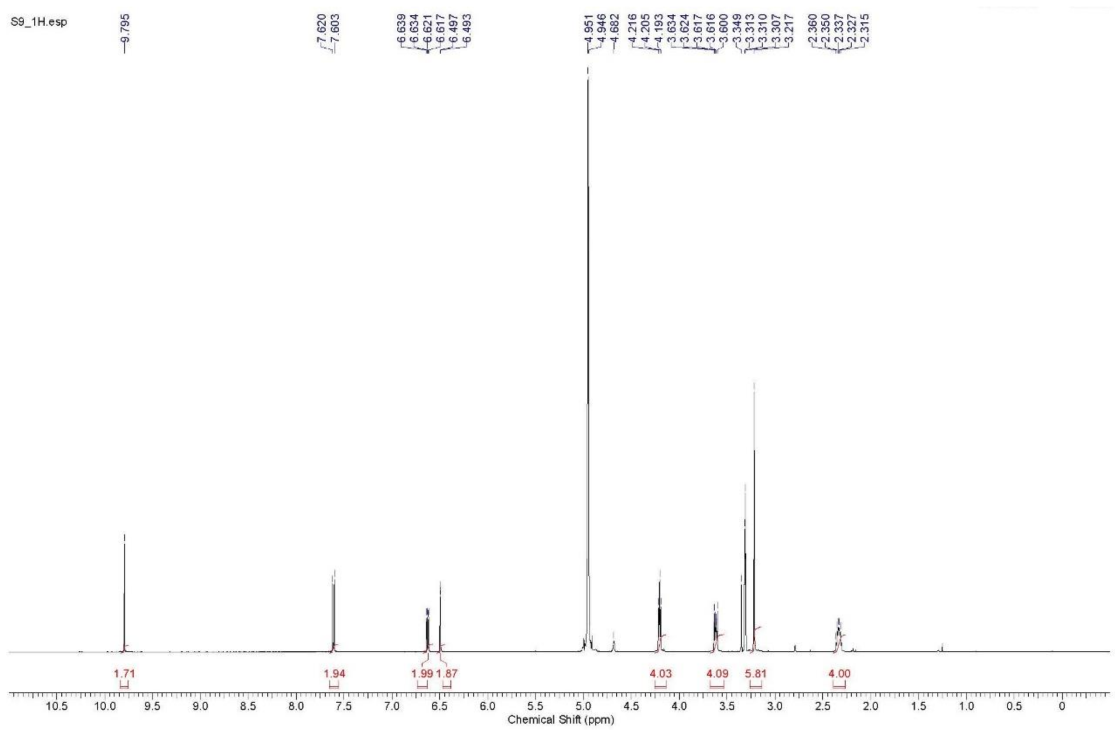
S9_1H.esp
9.795
7.620
7.603
6.639
6.634
6.621
6.617
6.497
6.493
4.951
4.946
4.682
4.216
4.205
4.193
3.634
3.624
3.617
3.616
3.600
3.349
3.313
3.310
3.307
3.217
2.360
2.350
2.337
2.327
2.315
1.71
1.94
1.99
1.87
4.03
4.09
5.81
4.00
Chemical Shift (ppm)

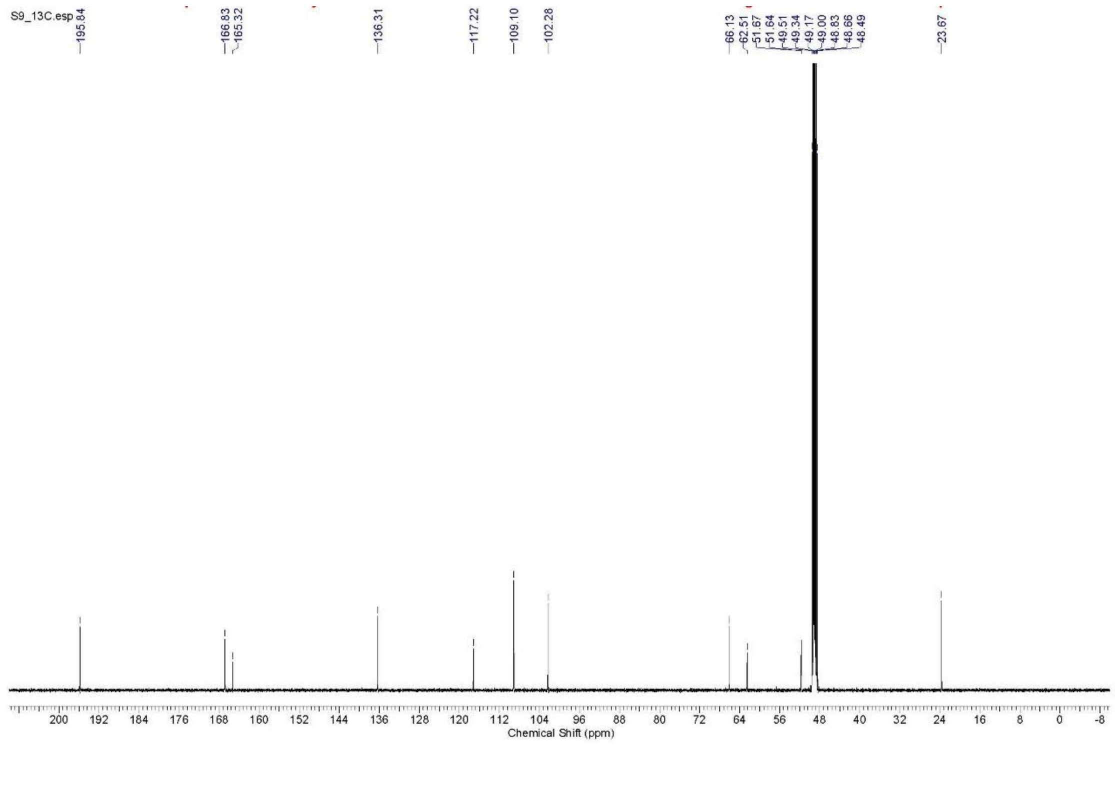
S9_13C.esp
195.84
166.83
165.32
136.31
117.22
109.10
102.28
66.13
62.51
51.67
51.64
49.51
49.34
49.17
49.00
48.83
48.66
48.49
23.67
Chemical Shift (ppm)

AB-C12

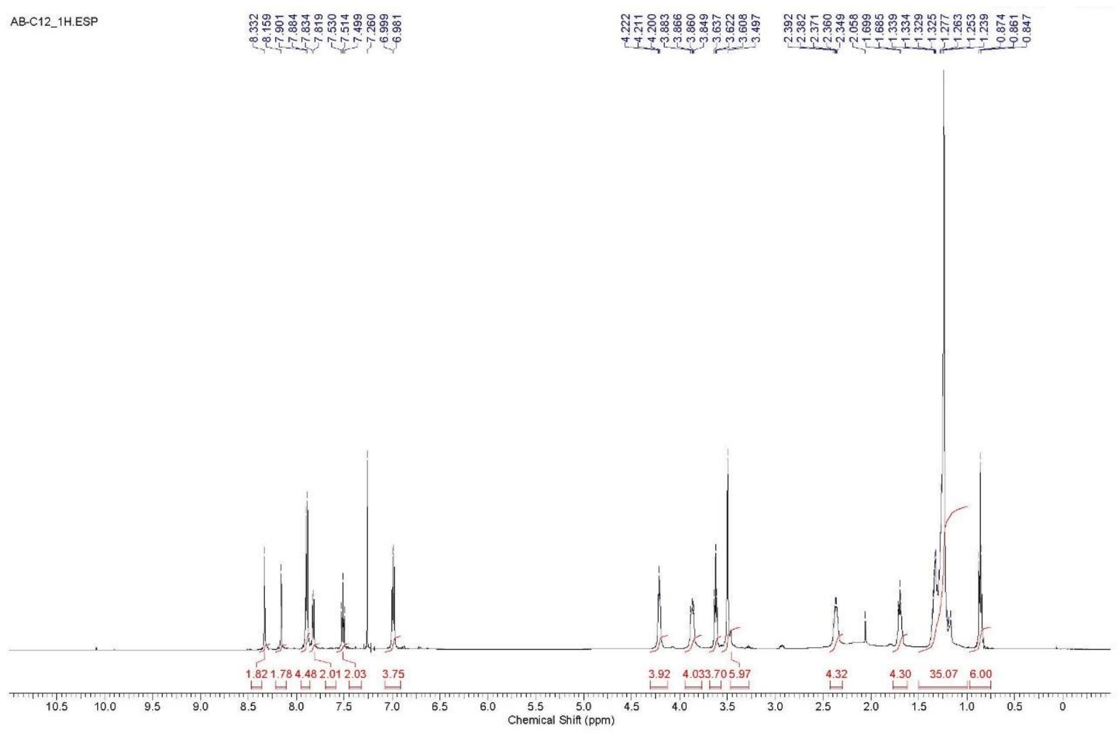
AB-C12_1H.ESP
Chemical Shift (ppm)

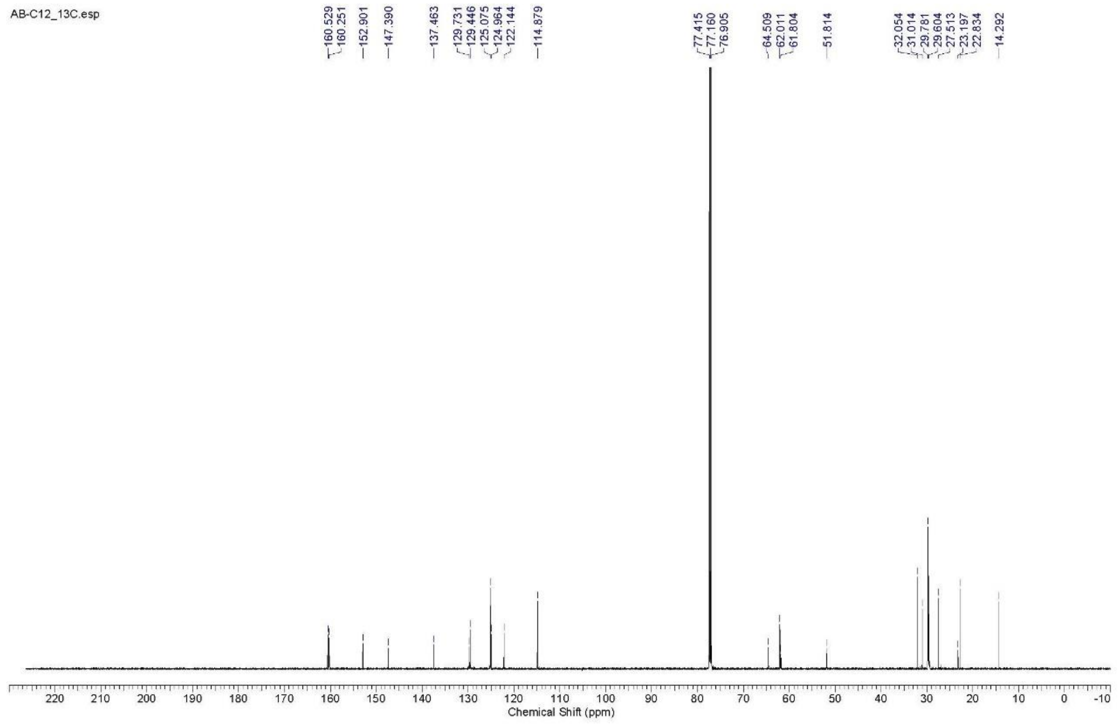
AB-C12_13C.esp
Chemical Shift (ppm)

AB-C10

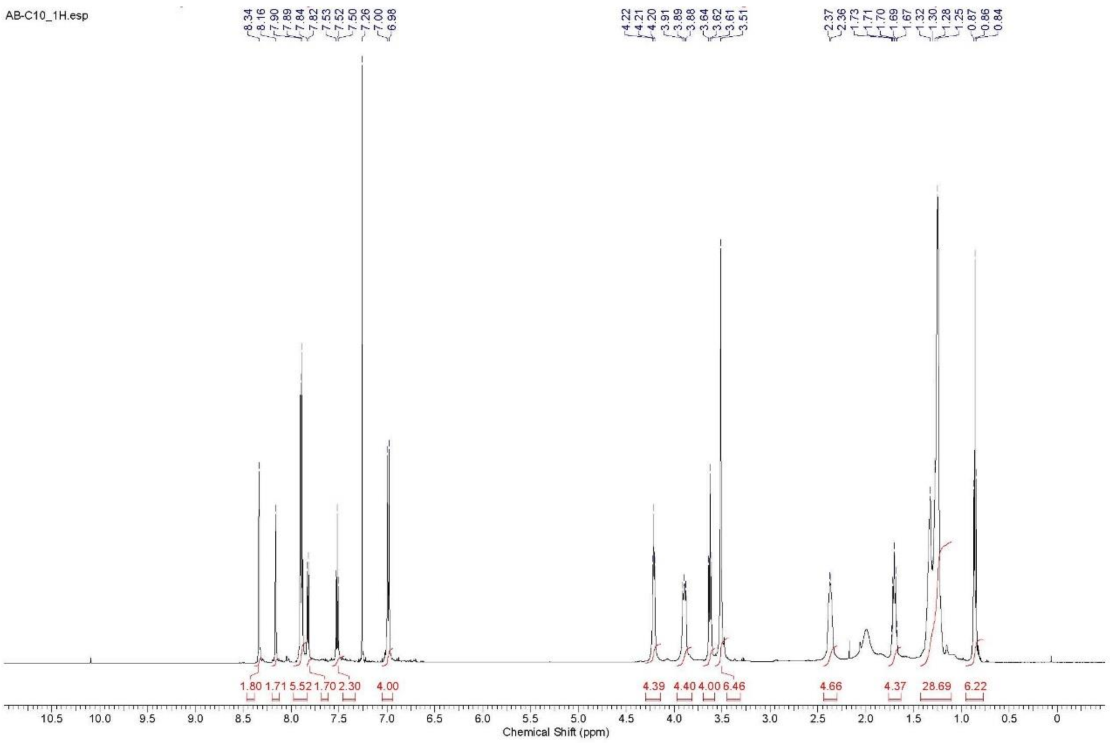

AB-C10_1H.esp
8.34 8.16 7.90 7.89 7.84 7.82 7.53 7.52 7.50 7.26 7.00 6.98
4.22 4.21 4.20 3.91 3.89 3.88 3.64 3.62 3.61 3.51
2.37 2.36 1.73 1.71 1.70 1.69 1.67 1.32 1.30 1.28 1.25 0.87 0.86 0.84
1.80 1.71 5.52 1.70 2.30 4.00
4.39 4.40 4.00 6.46
4.66 4.37 28.69 6.22
10.5 10.0 9.5 9.0 8.5 8.0 7.5 7.0 6.5 6.0 5.5 5.0 4.5 4.0 3.5 3.0 2.5 2.0 1.5 1.0 0.5 0
Chemical Shift (ppm)

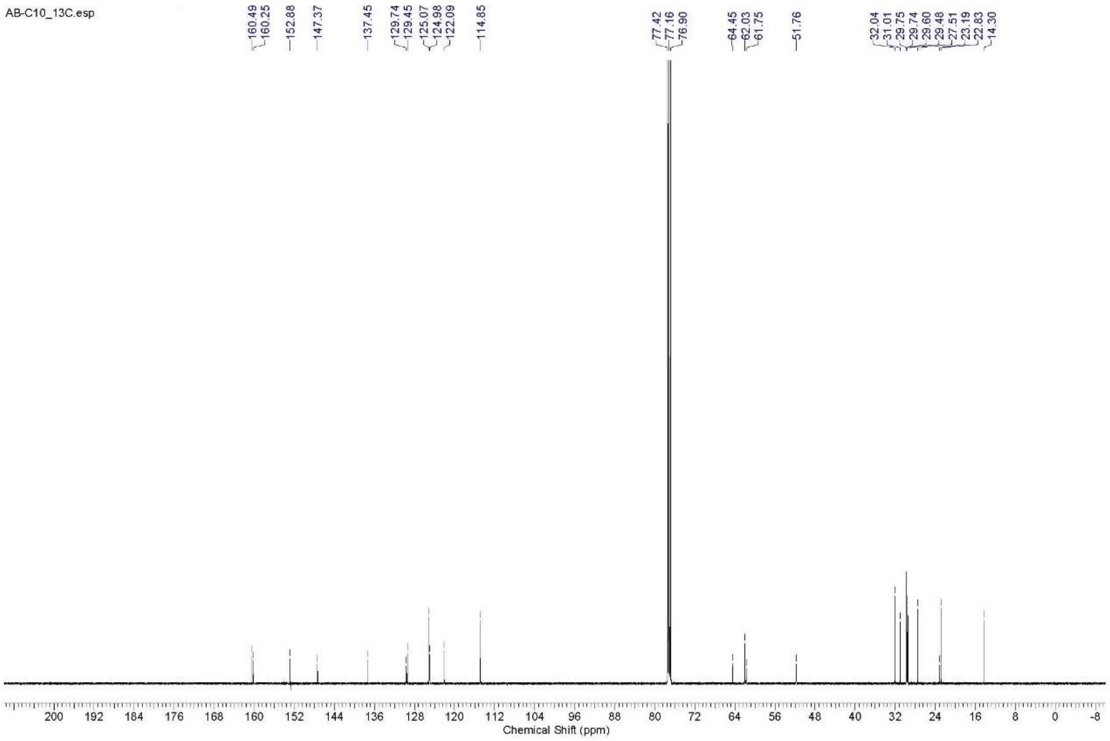

AB-C10_13C.esp
160.49 160.25 152.88 147.37 137.45 129.74 129.45 125.07 124.98 122.09 114.85
77.42 77.16 76.90 64.45 62.03 61.75 51.76
32.04 31.01 29.75 29.74 29.60 29.48 27.51 23.19 22.83 14.30
200 192 184 176 168 160 152 144 136 128 120 112 104 96 88 80 72 64 56 48 40 32 24 16 8 0 -8
Chemical Shift (ppm)

SA-C12

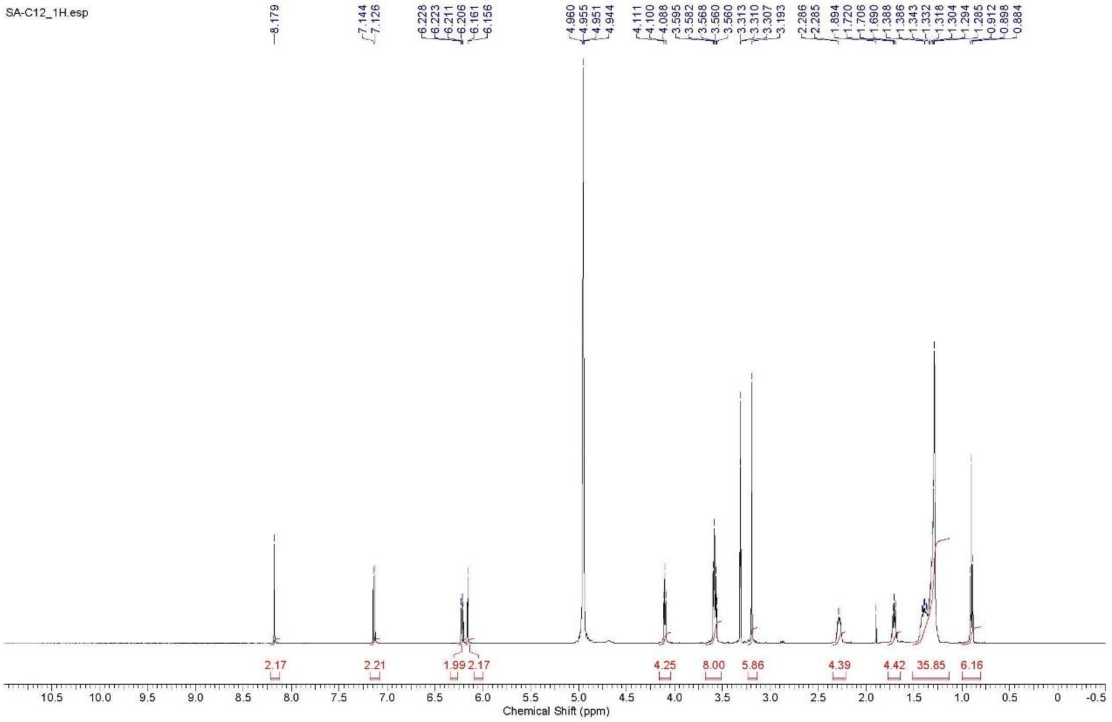
SA-C12_1H.esp
8.179
7.144
7.126
6.228
6.223
6.211
6.206
6.161
6.156
4.960
4.955
4.951
4.944
4.111
4.100
4.088
3.595
3.582
3.568
3.560
3.313
3.310
3.307
3.193
2.286
2.285
1.894
1.720
1.706
1.690
1.386
1.388
1.343
1.332
1.318
1.304
1.294
1.285
0.912
0.898
0.884
2.17 2.21 1.99 2.17 4.25 8.00 5.86 4.39 4.42 35.85 6.16
10.5 10.0 9.5 9.0 8.5 8.0 7.5 7.0 6.5 6.0 5.5 5.0 4.5 4.0 3.5 3.0 2.5 2.0 1.5 1.0 0.5 0 -0.5
Chemical Shift (ppm)

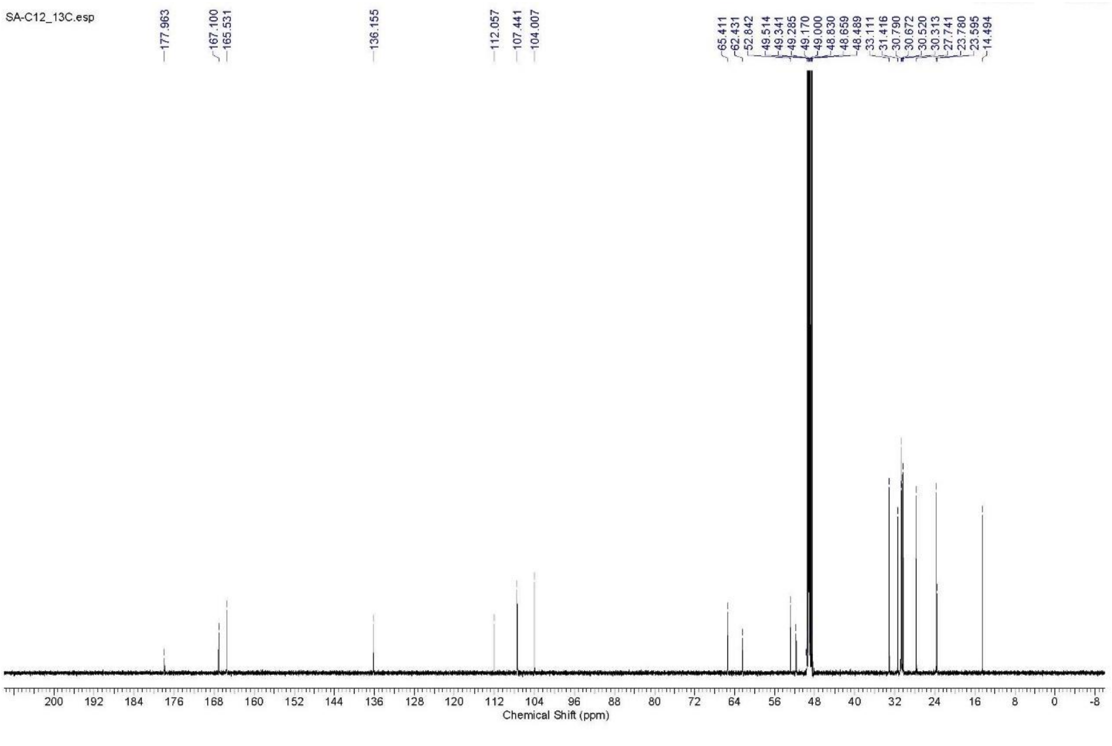
SA-C12_13C.esp
177.963
167.100
165.531
136.155
112.057
107.441
104.007
65.411
62.431
52.842
49.514
49.341
49.285
49.170
49.000
48.830
48.659
48.489
33.111
31.416
30.790
30.672
30.520
30.313
27.741
23.780
23.595
14.494
200 192 184 176 168 160 152 144 136 128 120 112 104 96 88 80 72 64 56 48 40 32 24 16 8 0 -8
Chemical Shift (ppm)

4. Supplementary figures S1 to S20

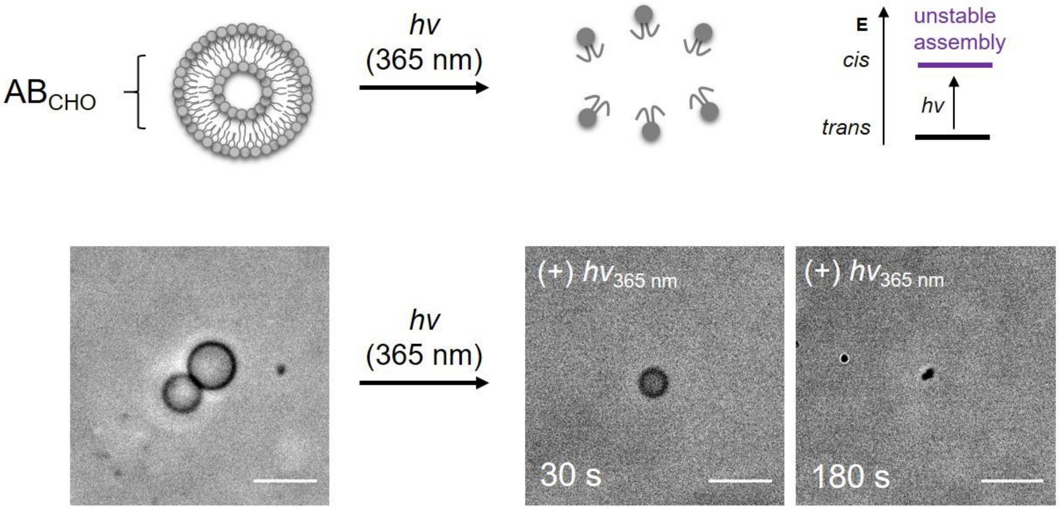

Figure S1. Light-induced disassembly of AB$_{CHO}$-based vesicles.

Top: Schematic illustration of 365 nm light-induced disassembly of vesicles consisting of **AB$_{CHO}$** building blocks. Bottom: Phase-contrast micrographs showing giant vesicles formed by **AB$_{CHO}$** (left), followed by their shrinking and disappearance under 365 nm light illumination (right). Images obtained in HEPES-buffered saline solution (pH 7.5, [HEPES] = 25 mM, [NaCl] = 50 mM). [**AB$_{CHO}$**]$_0$ = 1 mM, Scale bars, 10 µm.

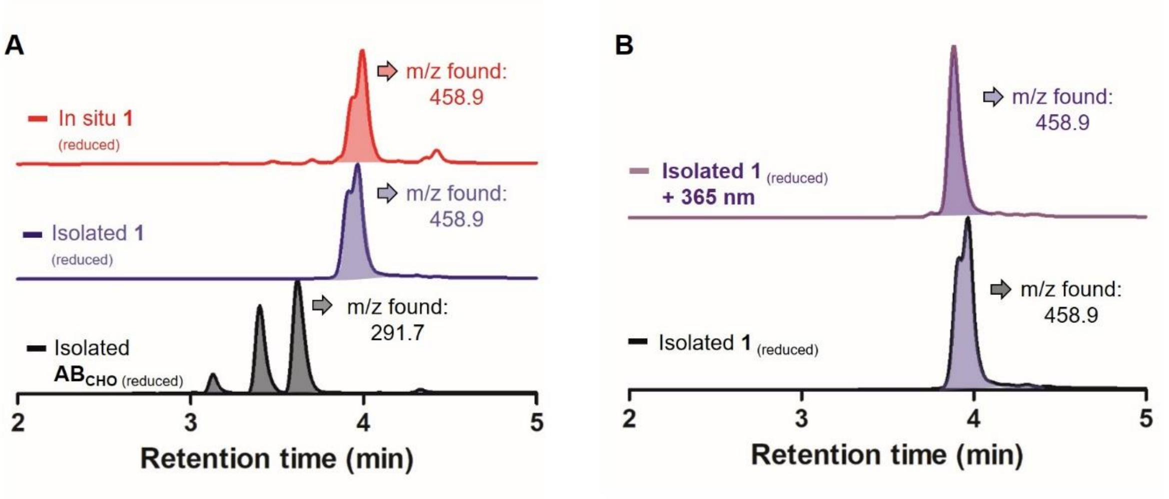

Figure S2. Characterization of in situ formed imine AB-C_{12} (compound 1) through reductive amination.

(A) LC-DAD chromatograms (at 250 nm) and observed mass values for reduced products of in situ formed **1** (top), isolated **1** (middle), and isolated starting material, **AB_{CHO}** (bottom). The observed mass values on the right correspond to the $[M+H]^{2+}/2$ of their reduced forms. The overlapping peaks observed in the chromatogram of in situ **1** correspond to *cis* and *trans* isomers, as demonstrated in (B). (B) The *trans*→*cis* isomerization of the reduced form of **1** under 365 nm light illumination, shown by the shift in chromatogram. In situ conditions: [**AB_{CHO}**]$_0$ = 1 mM, [**C_{12}-amine**]$_0$ = 2 mM in HEPES-buffered saline solution (pH 7.5, [HEPES] = 25 mM, [NaCl] = 50 mM), tumbling overnight at room temperature. Reduction conditions: $NaBH_4$ (100 equivalents) in 80 μL of methanol was mixed with 80 μL of buffered reaction solution, followed by sonication for 30 seconds.

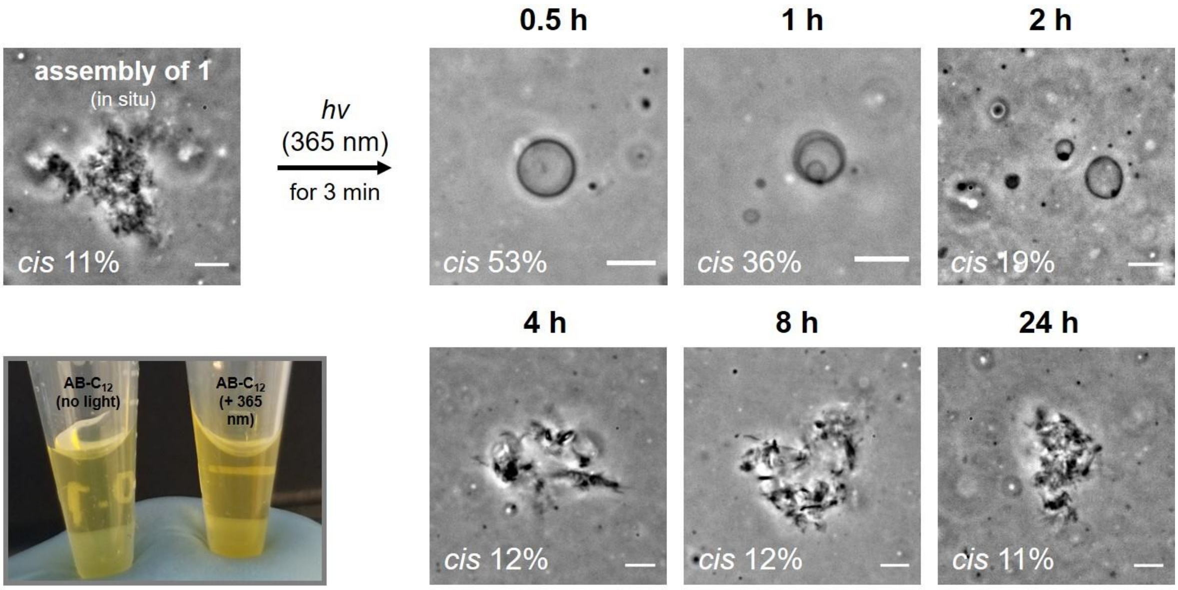

Figure S3. Time-dependent morphological changes of assemblies of AB-C_{12} (compound 1) after 365 nm illumination.

Time-series micrographs showing morphological changes of an in situ formed assembly of **1** after 365 nm light activation, followed by 24-hour exposure to ambient conditions without additional 365 nm irradiation. The percentage of *cis* composition was determined by LC-MS analysis at the indicated time points (see ‘Table S2’ for details). Ambient conditions: temperature, 20 °C; room light, 32 W-4100 K-fluorescent bulbs, 250–350 lux. Scale bars, 10 µm. The inset photograph represents the change in the reaction solution before and after 365 nm illumination.

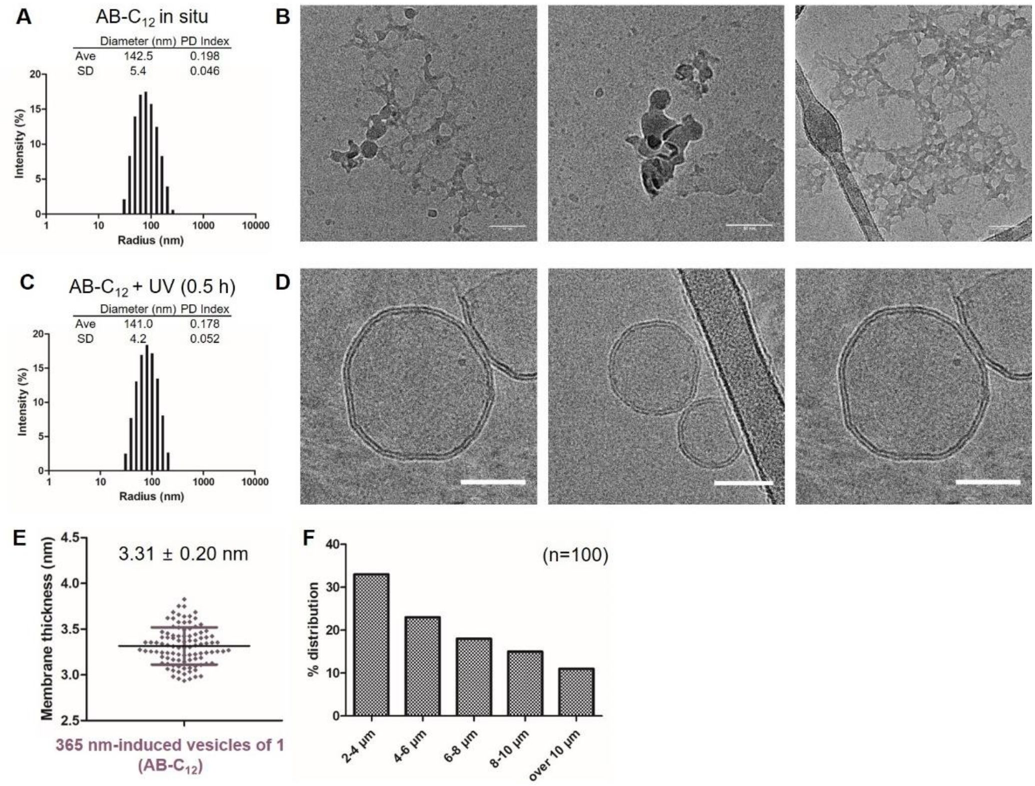

Figure S4. Characterization of light-driven assemblies of AB-C_{12} (compound 1).

(A) A histogram of sub-micron-sized assemblies of **AB-C_{12}** obtained from dynamic light scattering (DLS) analysis before exposure to 365 nm irradiation. The sample was collected from a section of the in situ reaction solution without suspended particles because large aggregates (over 10 μm) were not suitable for the DLS analysis. The table represents the average radius and polydispersity index (n=12). (B) Cryo-EM images of **AB-C_{12}** in situ assemblies before 365 nm illumination. Scale bars, 50 nm. (C) A DLS histogram of sub-micron-sized assemblies after 365 nm irradiation of the previously collected samples in (A), including their average radius and polydispersity index (n=12). (D) Cryo-EM images of **AB-C_{12}** assemblies after 365 nm illumination. Scale bars, 50 nm. (E) A scatter plot representing the measured bilayer thickness from ten different images (n=100). (F) A histogram depicting the relative size distribution (diameters) of micron-sized giant vesicles for light-driven **AB-C_{12}** assemblies, as determined by light microscopy analysis. (n=100). Vesicles with well-defined boundary structures exceeding 2 μm were considered for analysis.

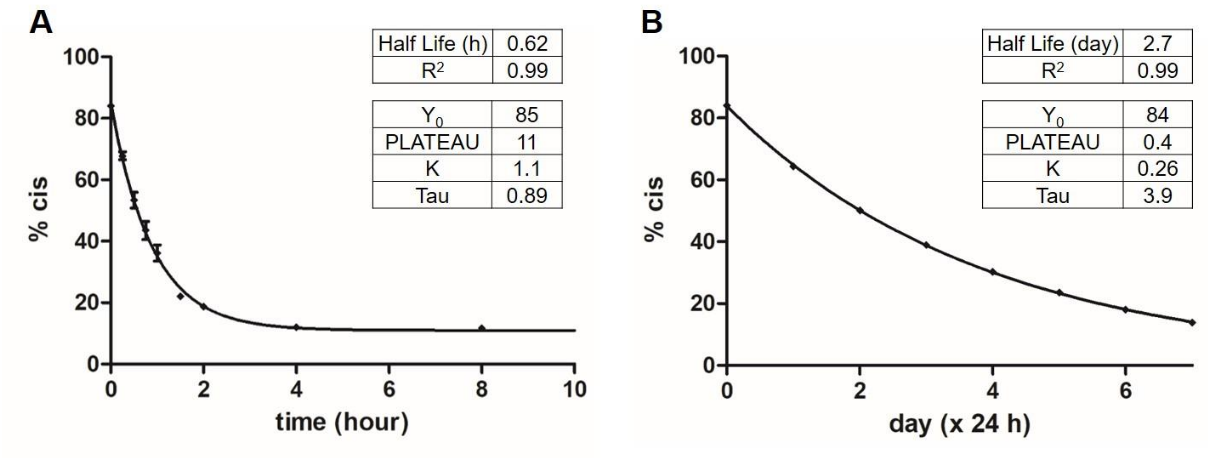

Figure S5. Kinetics of *cis*→*trans* isomerization of light-activated AB-C_{12} (compound 1).

Kinetic profiles illustrating the proportion of *cis* isomers under ambient conditions (A) and in dark (B) as a function of time after discontinuing 365 nm illumination. The graphs were fitted using a one-phase exponential decay model, following the equation Y = (Y_0 - Plateau) * exp(-K * X) + Plateau. Each value for (A) (at 0, 0.25, 0.5, 0.75, 1, 1.5, 2, 4, and 8 hour for A) or for (B) (at 1–7 days) was obtained from isomer analysis using LC-MS in triplicate. Error bars represent the standard deviation. Ambient conditions: temperature, 20 °C; room light, 32 W-4100 K-fluorescent bulbs, 250–350 lux.

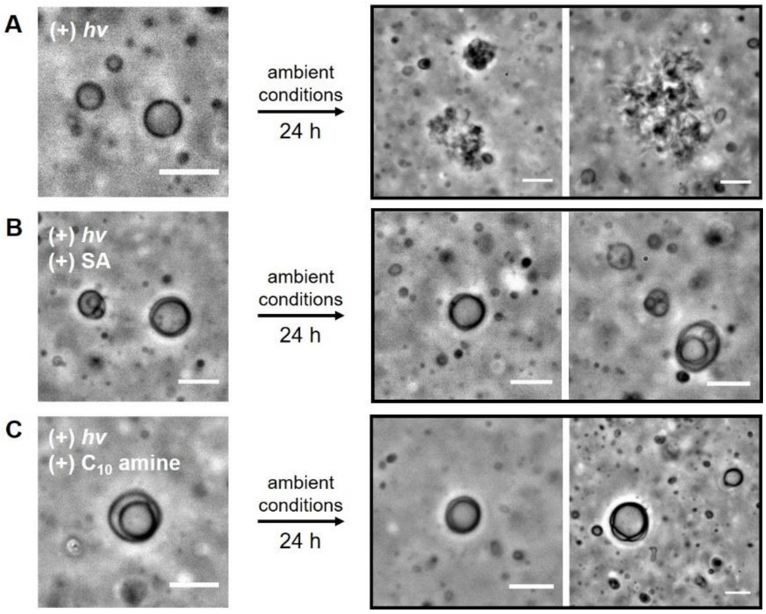

Figure S6. Morphological changes of remodeled membranes under ambient conditions for 24 hours.

Left: Phase-contrast micrographs showing vesicles prepared by 365 nm light activation, followed by subsequent lipid remodeling using chemical additives: (A) No additive control, (B) **SA** (1 equivalent), and (C) decylamine (C_{10}, 2.5 equivalent). Right: Phase-contrast micrographs captured after 24 hours of exposure to room lighting and temperature. Scale bars, 10 μm. Room conditions: temperature, 20 °C; room light, 32 W-4100 K-fluorescent bulbs, 250–350 lux.

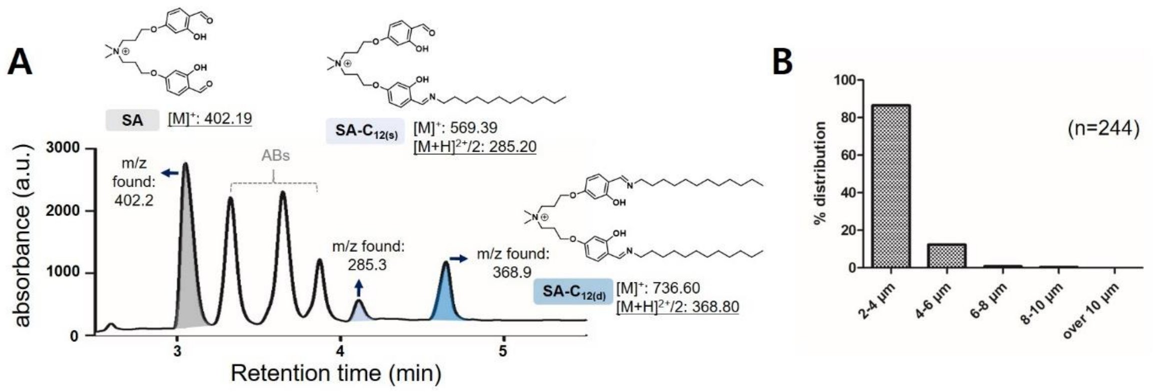

Figure S7. Representative chromatogram using a LC-DAD method (at 230 nm) of light-promoted head-group exchange of AB-C_{12} (compound 1) using SA and their relative size distributions.

(A) A chromatogram depicting the head-group exchange reaction between **1** and **SA**, performed after 365 nm light activation, followed by 24-hour tumbling under dark conditions. Reaction conditions: $[\mathbf{1}]_0$ = 1 mM, $[\mathbf{SA}]_0$ = 1 mM in HEPES-buffered saline solution (pH 7.5, [HEPES] = 25 mM, [NaCl] = 50 mM). (B) A histogram depicting the relative distribution of vesicle diameters after head-group exchange reactions (n=244).

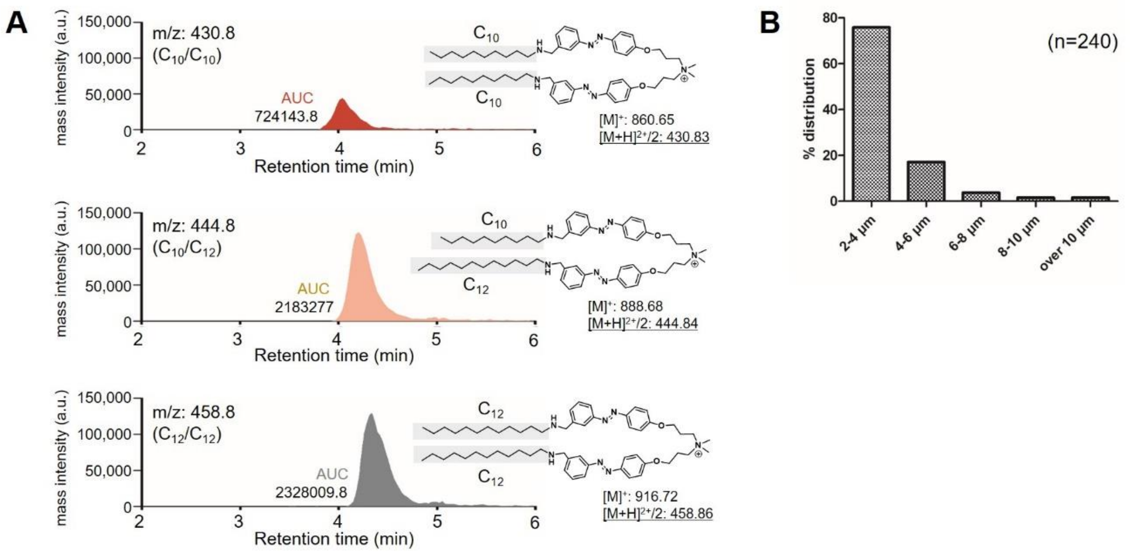

Figure S8. Representative extracted ion chromatograms (EICs) and corresponding chemical structures of tail exchanged products after reduction.

(A) Top: Double-tail exchanged products (C_{10}/C_{10}). Middle: single-tail exchanged product (C_{10}/C_{12}). Bottom: unexchanged starting material (C_{12}/C_{12}). Reducing conditions: $NaBH_4$ (100 equivalents) in 100 μL of methanol was mixed with 100 μL of tail-exchange reaction solution, followed by sonication for 30 seconds. (B) A histogram showing the relative distribution of vesicle diameters after tail exchange reactions (n=240).

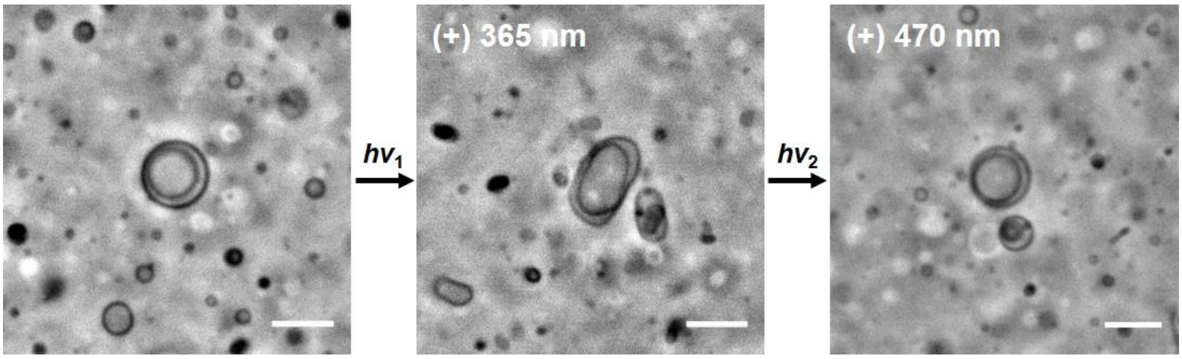

Figure S9. Light-responsive shape changes of C_{10}-tailed vesicles.

Phase-contrast micrographs showing the shape changes of tail-exchanged vesicles of **1** ($C_{12} \rightarrow C_{10}$) in three different conditions: initial ambient conditions (left), under 365 nm illumination (middle), and under subsequent 470 nm irradiation (right.) Scale bars, 10 μm.

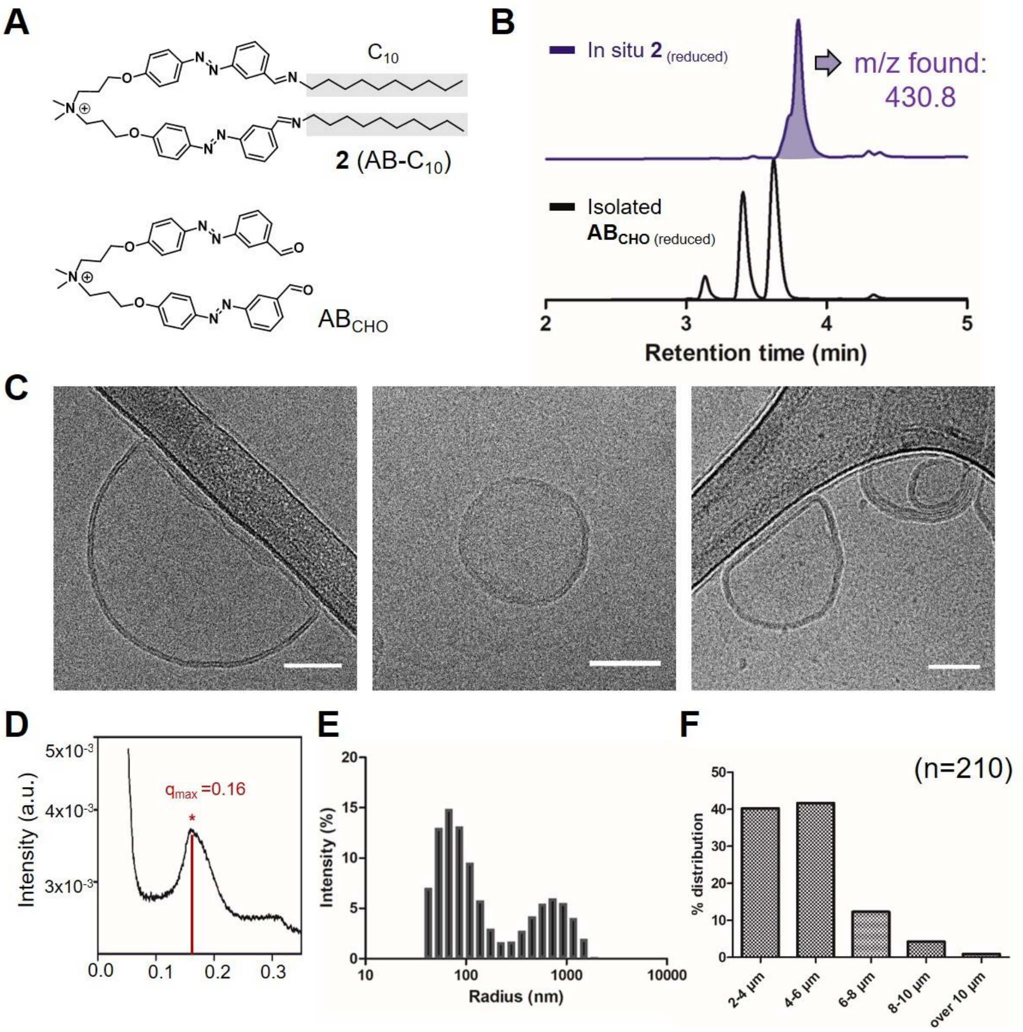

Figure S10. Characterization of vesicles formed after in situ formation of AB-C_{10} (compound 2).

(A) Chemical structures of the aldehyde precursor (**AB_{CHO}**) and the corresponding imine product (**2**) formed by reacting with decylamine (C_{10}). (B) LC-DAD chromatograms at 250 nm and the corresponding mass value found in MS chromatogram for reduced products of in situ formed **2** (top) compared to **AB_{CHO}** (bottom). The mass value on the right corresponds to $[M+H]^{2+}/2$ of reduced **2**. In situ conditions: [**AB_{CHO}**]$_0$ = 0.5 mM, [**C_{10} amine**]$_0$ = 2 mM in HEPES-buffered saline solution (pH 7.5, [HEPES] = 25 mM, [NaCl] = 50 mM), tumbling overnight at room temperature. Reduction conditions: $NaBH_4$ (100 equivalents) in 80 μL of methanol was mixed with 80 μL of the in situ reaction solution, followed by sonication for 30 seconds. (C) Cryo-EM images showing the bilayer structures of assemblies of **2** (**AB-C_{10}**). Scale bars, 50 nm. (D) Small-angle X-ray scattering (SAXS) profile from **AB-C_{10}** products. The spacing between the lamellar bilayers for **AB-C_{10}** was estimated to be 3.9 nm (d = $2\pi/q_{max}$). (E) A DLS histogram of in situ formed **AB-C_{10}** assemblies. (F) A histogram depicting the relative distribution of vesicle diameters for in situ formed **AB-C_{10}** giant vesicles (n=210).

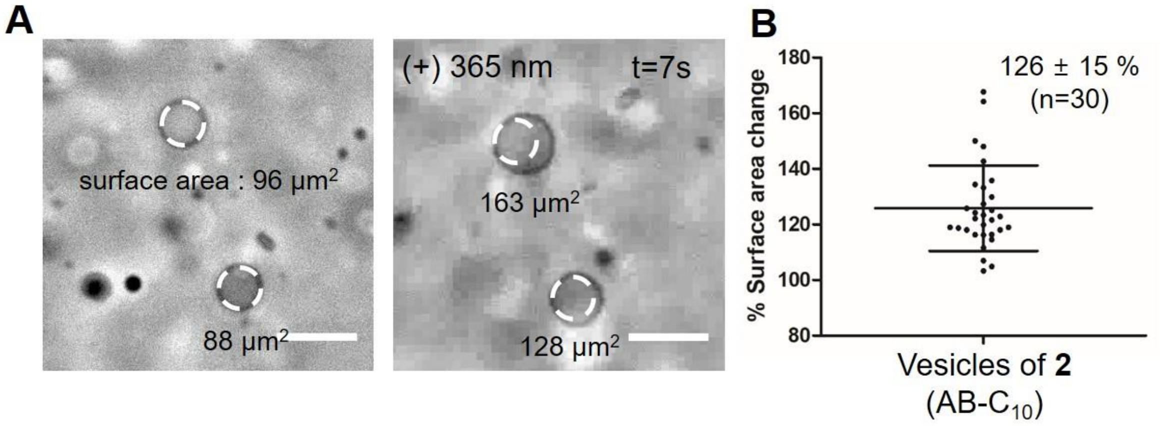

Figure S11. Light-induced surface expansion of vesicles consisting of AB-C_{10} (compound 2).

(A) Left: Phase-contrast micrographs of vesicles of **2** before light illumination. Right: Vesicles under 365 nm illumination (at 7 seconds). Scale bars: 10 μm. (B) Scatter plot depicting changes in surface area of vesicles of **2** before and after 365 nm illumination. To examine the surface area expansion during 365 nm irradiation, vesicles were chosen and measured immediately prior to elongation becoming observable.

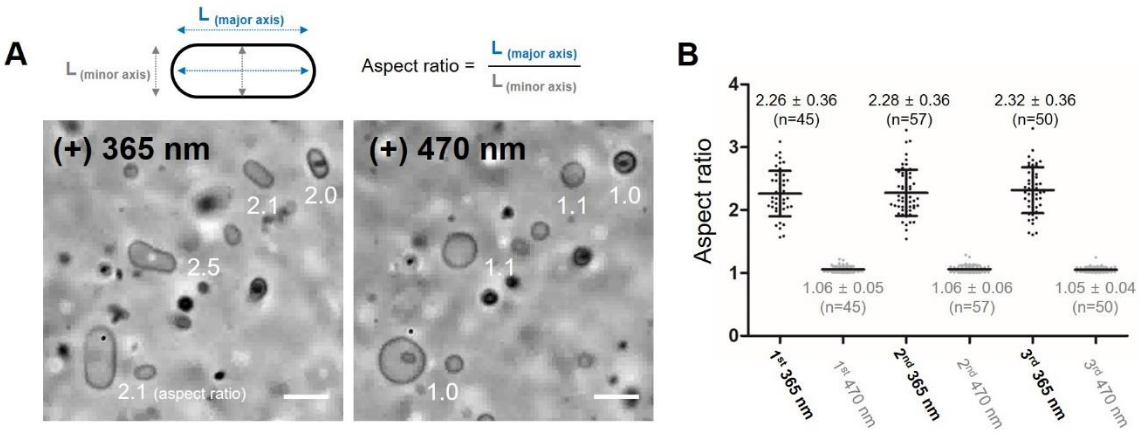

Figure S12. Light-induced aspect ratio changes of vesicles consisting of AB-C_{10} (compound 2) under 365 and 470 nm illumination.

(A) Top: Scheme depicting the aspect ratio measurement. Bottom: Phase-contrast micrographs of vesicles of **2** under 365 nm illumination (left) and under 470 nm illumination (right). Scale bars, 10 µm. (B) Scatter plot of the measured aspect ratio under three alternating illuminations of 365 and 470 nm LED lights. The numbers indicate the average ± SD.

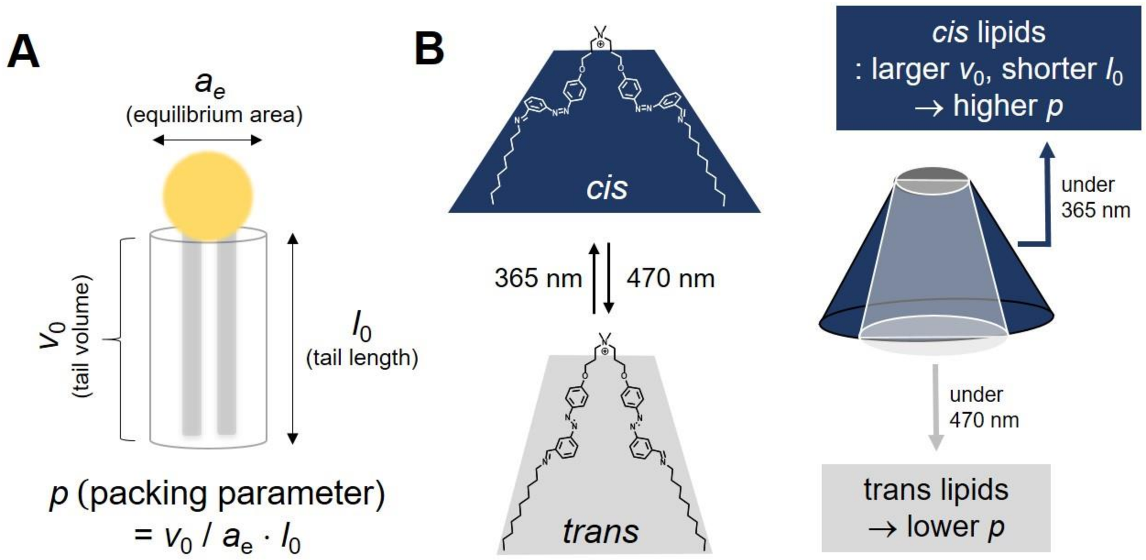

Figure S13. Chemical structures and relative packing properties of photoisomers of AB-C_{10} (compound 2).

(A) Scheme representing the packing parameter[S10] for a surfactant with a hydrophilic head (yellow) and hydrophobic tails (gray). *p*, packing parameter, v_0, surfactant tail volume, $l_{0,}$, surfactant tail length, a_e, equilibrium area per molecule. (B) Left: light-induced isomerization between *trans* and *cis* tailed-**2**. Right: Qualitative comparison of relative packing parameters between *trans* and *cis* tailed-**2**.

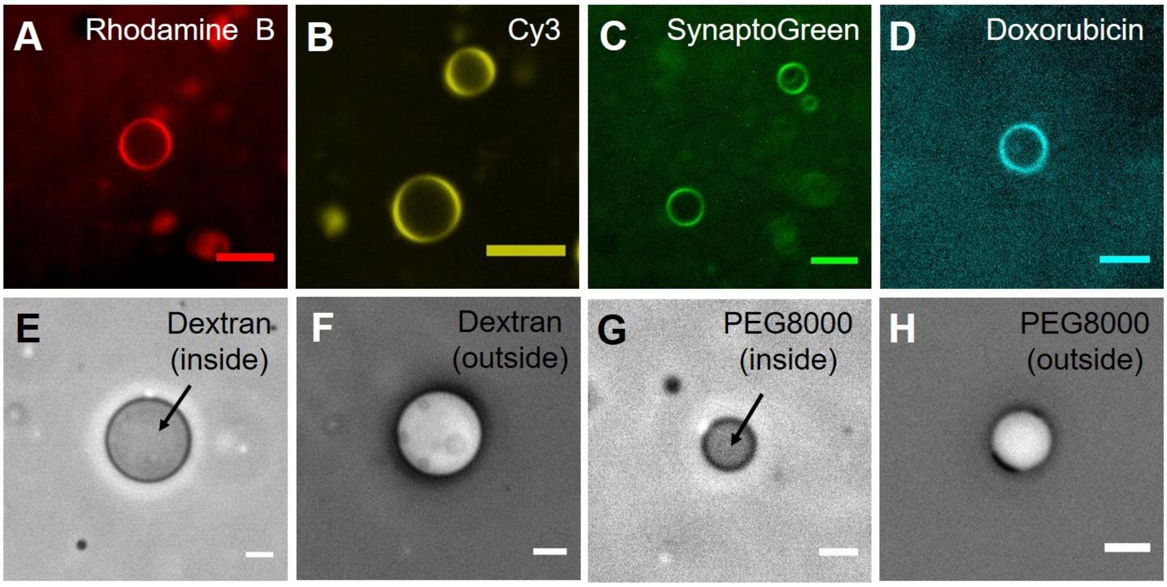

Figure S14. Encapsulating properties of vesicles of AB-C_{10} (compound 2) for aromatic and non-aromatic compounds.

(A–D) Confocal fluorescence micrographs of membranes based on **2** after adding 1 mol% of (A) Rhodamine B, (B) Cy3-alkyne, (C) SynaptoGreen, and (D) Doxorubicin, without further washing or dilution. (E and G) Phase-contrast micrographs after performing 1:10 (v/v) dilution by adding extra blank buffer (10-fold volume) to in situ vesicles of **2** prepared with (E) 5% (w/v) dextran, and (G) 5% (w/v) PEG 8000. (F and H) Phase-contrast micrographs after performing 1:10 (v/v) dilution by adding a solution (10-fold volume) containing (F) 5% (w/v) dextran, and (H) 5% (w/v) PEG 8000, to in situ vesicles of **2** (no additive inside). Scale bars, 5 µm.

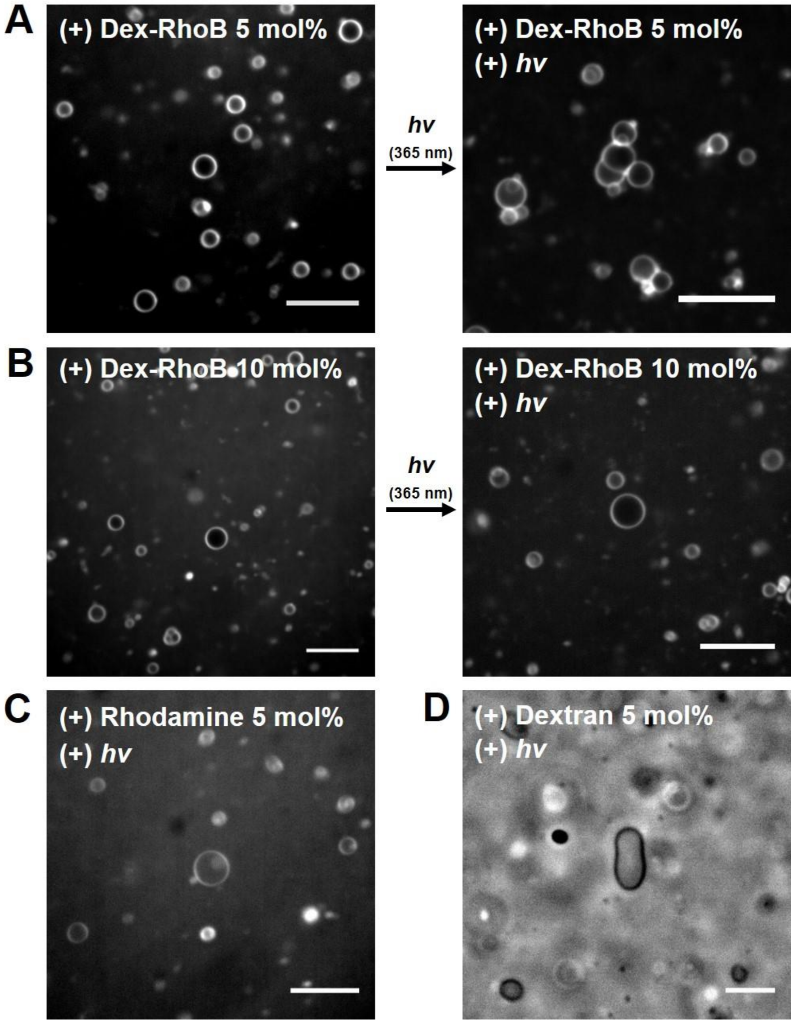

Figure S15. Light-driven properties of vesicles of AB-C_{10} (compound 2) after treatment with various additives.

(A) Confocal fluorescence micrographs of vesicles of **2** after treatment with 5 mol% of Dex-RhoB under ambient conditions (left) and successive irradiation with 365 nm light stimulus (right). (B) Fluorescence micrographs of vesicles of **2** after treatment with 10 mol% of Dex-RhoB (left) and under 365 nm light stimulus (right). (C) Fluorescence micrograph of vesicles of **2** after treatment with 5 mol% of Rhodamine B under 365 nm illumination. (D) Phase-contrast image of vesicles of **2** treated with 5 mol% of dextran (average molecular weight: 10,000) under 365 nm illumination. Scale bars, 10 μm.

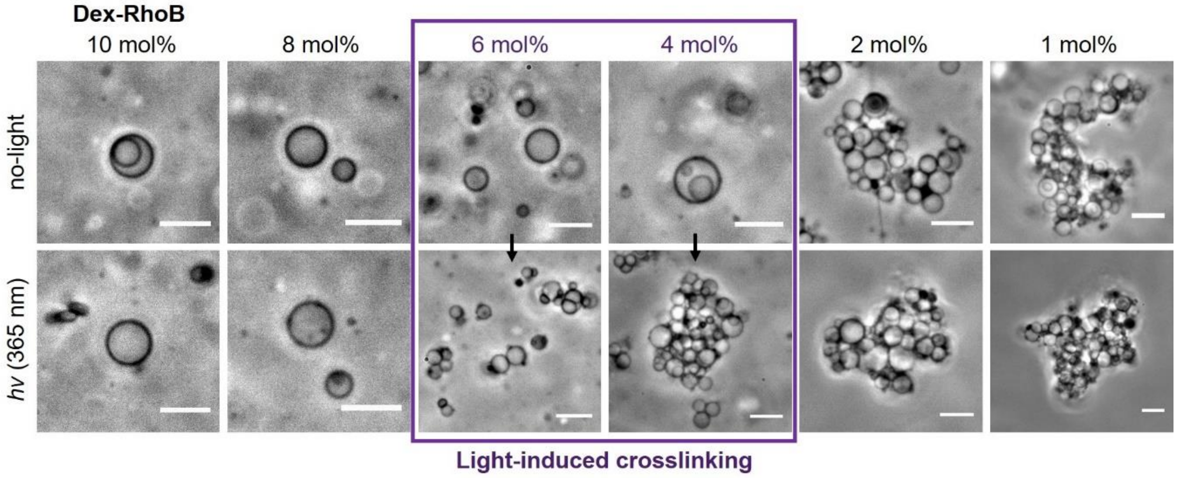

Figure S16. Dex-RhoB-concentration dependent crosslinking of vesicles based on AB-C_{10} (compound 2).

Phase-contrast images showing the crosslinking properties of vesicles of **2** after exposure to varying concentrations of Dex-RhoB under ambient conditions (top) and under 365 nm illumination (bottom). Scale bars, 10 µm.

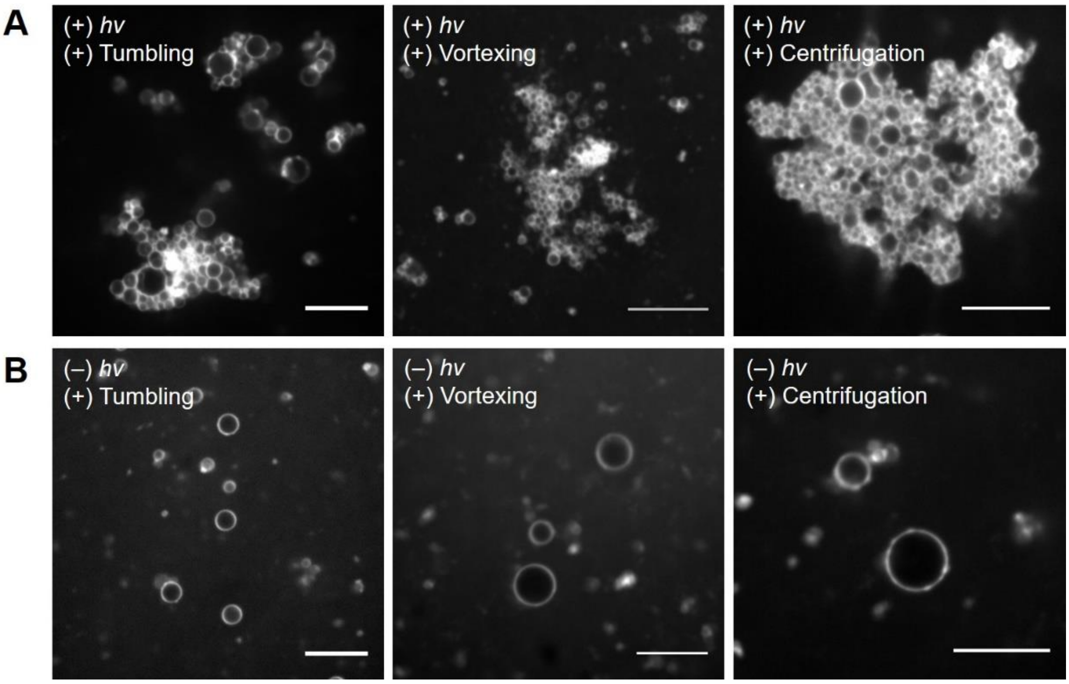

Figure S17. Physical agitation facilitates the light-driven crosslinking of vesicles of AB-C_{10} (compound 2).

(A) Confocal fluorescence micrographs of 5 mol% Dex-RhoB-coated vesicles of **2** under 365 nm light treatment, demonstrating crosslinking under various physical agitation conditions: tumbling for 1 hour (left), vortexing for 3 minutes (middle), or centrifugation for 5 minutes at 132,000 rcf (right). (B) Same physical agitation treatments but in the absence of light stimulus. Scale bars, 10 μm.

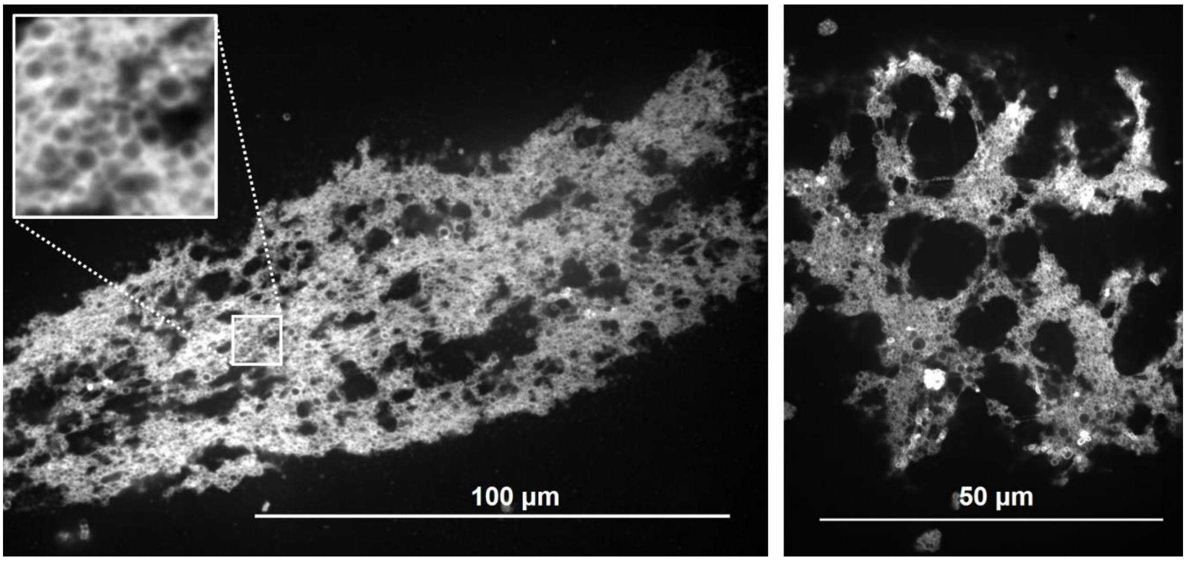

Figure S18. Representative images of giant synthetic tissues.

Confocal fluorescence micrographs of synthetic tissue networks exhibiting sizes over 50 um, prepared by 365 light stimulus and successive centrifugation (132,000 rcf for 5 min) of 5 mol% Dex-RhoB-coated vesicles of **2**. Inset: An enlarged image of a local area. Size, 10 µm x 10 µm.

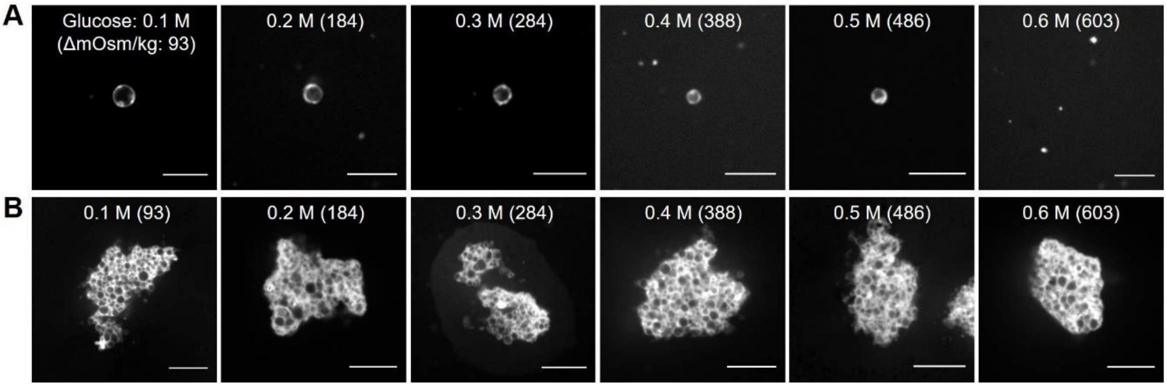

Figure S19. Stability comparison between vesicles and synthetic tissues under various osmotic pressures.

Confocal fluorescence micrographs of vesicles of **2** coated with 5 mol% Dex-RhoB (A) and their light-driven synthetic tissues (B) under a range of osmotic shocks induced by adding 0.1–0.6 M glucose solution (10-fold volume). Note that vesicles under glucose concentrations exceeding 0.3 M were extremely rare and difficult to find in their intact, round shape. Numbers in parenthesis indicate the differences in osmolality (mOsm/kg) between the initial in situ conditions and the external glucose solutions (osmotic shock). Scale bars, 10 μm.

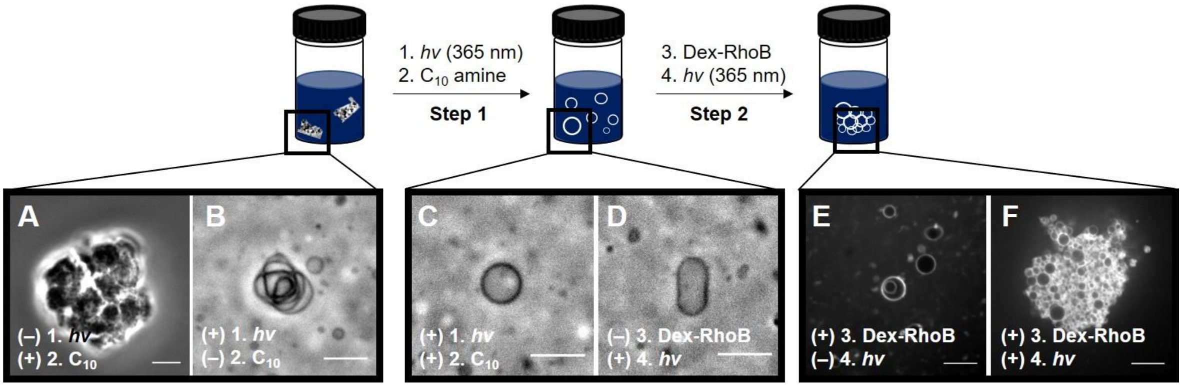

Figure S20. Stepwise one-pot transformation of lipid aggregates to synthetic tissues.

(A–B) Phase-contrast micrographs of the negative controls in the first reaction step in the absence of (A) 365 nm light or (B) C_{10} precursor. (C) Phase-contrast micrograph of the first step product generating vesicle assembly. (D–E) Negative controls in the second reaction step in the absence of (D) Dex-RhoB or (E) 365 nm light illumination. (F) Fluorescent micrograph of the second step products generating synthetic tissues. Reaction conditions: First step, $[\mathbf{1}]_0 = 0.8$ mM, $[C_{10}] = 4$ mM, $h\nu = 365$ nm illumination for 3 minutes. Second step, [Dex-RhoB] = 5 mol% compared to $[\mathbf{1}]_0$, $h\nu = 365$ nm illumination for 3 minutes followed by centrifugation at 132,000 rcf for 5 minutes. Scale bars, 10 μm.

5. Supplementary tables S1 to S8

Table S1. Isomer distribution of in situ product of **1** under varying light conditions (n=3)

	**%** ***c/c*** (SD)	**%** ***c/t*** (SD)	**%** ***t/t*** (SD)
**room light** (fluorescent bulb, 32 W, 4100 K)	2 (0.1)	18 (0.6)	80 (0.6)
**under 365 nm**	72 (0.4)	24 (0.2)	4 (0.4)
**under 470 nm**	2 (0.0)	19 (0.1)	79 (0.1)

Table S2. Temporal changes in the proportion of light-produced *cis*-isomers in **1** under room light or in the dark (n=3)

***cis*-1** (under room light)			***cis*-1** (in dark)		
**Time**	**Average (%)**	**SD (%)**	**Time**	**Average (%)**	**SD (%)**
0 h	84	0.7	0 h	84	0.7
0.25 h	68	1.1	24 h	64	0.1
0.5 h	53	2.1	48 h	50	0.1
0.75 h	44	2.4	72 h	39	0.3
1 h	36	2.2	96 h	30	0.1
1.5 h	22	0.5	120 h	24	0.2
2 h	19	0.7	144 h	18	0.2
4 h	12	0.2	168 h	14	0.6
8 h	12	0.2			
24 h	11	0.4			

Table S3. Integration of the area under the curve of LC-DAD chromatograms (at 230 nm) under no-light control condition

	**3.0 min** (SA)	**4.1 min** ($SA-C_{12}$, single tail)	**4.6 min** ($SA-C_{12}$, double tails)	**Total**
**Sample 1 (no-light)**	17896.6	252.7	1033.4	19182.7
%	93.3	1.3	5.4	
**Sample 2 (no-light)**	17842.2	405.7	1097.1	19345
%	92.2	2.1	5.7	
**Sample 3 (no-light)**	17819.7	307.7	1012.2	19139.6
%	93.1	1.6	5.3	
**Average (%)**	92.9	1.7	5.4	
**SD**	0.5	0.3	0.2	

Table S4. Integration of the area under the curve of LC-DAD chromatograms (at 230 nm) under 365 nm light condition

	**3.0 min** (SA)	**4.1 min** ($SA-C_{12}$, single tail)	**4.6 min** ($SA-C_{12}$, double tails)	**Total**
**Sample 1 (+365 nm)**	13983.7	1605.2	4426.2	20015.1
%	69.9	8	22.1	
**Sample 2 (+365 nm)**	13937.8	1739.4	4546.8	20224
%	68.9	8.6	22.5	
**Sample 3 (+365 nm)**	14049.8	1330.4	4494.9	19875.1
%	70.7	6.7	22.6	
**Average (%)**	69.8	7.8	22.4	
**SD**	0.7	0.8	0.2	

Table S5. Integration of the area under the curve of extracted ion chromatograms under no-light control condition

	**C_{10} / C_{10} (m/z: 430.8)**	**C_{10} / C_{12} (m/z: 444.8)**	**C_{12} / C_{12} (m/z: 458.8)**	**Total**
**Sample 1 (no-light)**	236130.5	875284.1	4432236	5543650.6
**%**	4.2	15.8	80.0	
**Sample 2 (no-light)**	229391.2	833419.6	4219275	5282085.8
**%**	4.3	15.8	79.9	
**Sample 3 (no-light)**	230086.3	853286.3	4080641.5	5164014.1
**%**	4.5	16.5	79.0	

**Average (%)**	4.4	16.0	79.6
**SD**	0.1	0.3	0.4

Table S6. Integration of the area under the curve of extracted ion chromatograms under 365 nm light condition

	**C_{10} / C_{10} (m/z: 430.8)**	**C_{10} / C_{12} (m/z: 444.8)**	**C_{12} / C_{12} (m/z: 458.8)**	**Total**
**Sample 1 (+365 nm)**	724143.8	2183277	2328009.8	5235430.6
**%**	13.8	41.7	44.5	
**Sample 2 (+365 nm)**	692906.5	2000537.9	2197486	4890930.4
**%**	14.2	40.9	44.9	
**Sample 3 (+365 nm)**	652623.5	2070652.8	2299357.3	5022633.6
**%**	13.0	41.2	45.8	

**Average (%)**	13.7	41.3	45.0
**SD**	0.5	0.3	0.5

Table S7. Isomer distribution of in situ product of **2** under 365 and 470 nm LED lights (n=3)

	**%** ***c/c*** (SD)	**%** ***c/t*** (SD)	**%** ***t/t*** (SD)
**under 365 nm**	71 (0.4)	23 (0.2)	6 (0.4)
**under 470 nm**	2 (0.0)	20 (0.3)	78 (0.3)

Table S8. Measurement of osmolality (mOsm/kg) in solutions containing a range of glucose concentrations (Blank: HEPES 25 mM and 50 mM NaCl, pH 7.5)

**Glucose**	**0 M**	**0.1 M**	**0.2 M**	**0.3 M**	**0.4 M**	**0.5 M**	**0.6 M**
Osmolality (mOsm/kg)	121	214	305	405	509	607	724
ΔmOsm/kg (compared to blank)	-	93	184	284	388	486	603

6. Captions for movies S1 to S4

Movie S1.

Light-triggered vesicle formation

Time-lapse video showing the transition of in situ formed disordered lipid aggregates of compound **1** (**AB-C$_{12}$**) into fluidic membrane-bound assemblies under exposure to 365 nm LED light for 3 minutes. In situ conditions: [**AB$_{CHO}$**]$_0$ = 1 mM, [C$_{12}$-amine]$_0$ = 2 mM in HEPES-buffered saline solution (pH 7.5, [HEPES] = 25 mM, [NaCl] = 50 mM), tumbled overnight at room temperature. The video was recorded using phase-contrast microscopy (50 ms exposure) from a 2 μL sample of the overnight in situ mixture.

Movie S2.

Light-induced vesicle expansion

Time-lapse video depicting the temporary expansion of in situ formed vesicles (compound **2**, **AB-C$_{10}$**) under 365 nm LED light for the initial 10 seconds. The subsequent shape change (Movie S3, sphere to rod-like) occurs following this transient expansion. In situ conditions: [**AB$_{CHO}$**]$_0$ = 0.5 mM, [C$_{10}$-amine]$_0$ = 2 mM in HEPES-buffered saline solution (pH 7.5, [HEPES] = 25 mM, [NaCl] = 50 mM), tumbled overnight at room temperature. The video was captured from a 2 μL sample of the overnight in situ mixture using phase-contrast microscopy (50 ms exposure).

Movie S3.

Light-driven reversible shape changes

Time-lapse video illustrating the reversible shape changes of in situ formed vesicles consisting of compound **2** (**AB-C$_{10}$**) under alternating illumination of 365 nm and 470 nm LED light. The video displays three cycles of alternation, with each irradiation lasting 1 minute. The same in situ reaction conditions and microscope settings from Movie S2 were employed.

Movie S4.

Light-driven vesicle crosslinking

Time-lapse video demonstrating light-induced crosslinking of vesicles consisting of **2** (**AB-C$_{10}$**) treated with 5 mol% of Dex-RhoB polymer. The video was recorded under 365 nm LED light illumination for 3 minutes. Fluorescence was captured using a mercury light source coupled with a Texas Red filter, integrated into the light microscopy setup. The video was taken from a 2 μL sample of the overnight in situ mixture (**AB-C$_{10}$**, as depicted in Movie S2), which had an additional treatment of 5 mol% of Dex-RhoB.

7. References

(S1) Fulmer, G. R.; Miller, A. J. M.; Sherden, N. H.; Gottlieb, H. E.; Nudelman, A.; Stoltz, B. M.; Bercaw, J. E.; Goldberg, K. I. NMR Chemical Shifts of Trace Impurities: Common Laboratory Solvents, Organics, and Gases in Deuterated Solvents Relevant to the Organometallic. *Organometallics* **2010**, *29*, 2176–2179.

(S2) McMurray, T. A.; Byrne, J. A.; Dunlop, P. S. M.; McAdams, E. T. Photocatalytic and electrochemically assisted photocatalytic oxidation of formic acid on TiO_2 films under UVA and UVB irradiation. *J. Appl. Electrochem.* **2005**, *35*, 723–731.

(S3) García-Amorós, J.; Velasco, D. Recent advances towards azoben-zene-based light-driven real-time information-transmitting materials. *Beilstein J. Org. Chem.* **2012**, *8*, 1003–1017.

(S4) Ryhänen, S. J.; Säily, V. M. J.; Parry, M. J.; Luciani, P.; Mancini, G.; Alakoskela, J.-M. I.; Kinnunen, P. K. J. Counterion-Controlled Transition of a Cationic Gemini from Submicroscopic to Giant Vesicles. *J. Am. Chem. Soc.* **2006**, *128*, 8659–8663.

(S5) Mirgorodskaya, A. B.; Kudryavtseva, L. A.; Zuev, Yu. F.; Archipov, V. P.; Idiyatullin, Z. Sh.; Kudryavtsev, D. B. The influence of hydrophobic amines on hydrolysis of bis(*p*-nitrophenyl) ethylphosphonate in micellar solutions of cetylpyridinium bromide. *Russ. Chem. Bull.* **2000**, *49*, 270–275.

(S6) Parasassi, T.; De Stasio, G.; Ravagnan, G.; Rusch, R. M.; Gratton, E. Quantitation of lipid phases in phospholipid vesicles by the generalized polarization of Laurdan fluorescence. *Biophys. J.* **1991**, *60*, 179–189.

(S7) Budin, I.; Debnath, A.; Szostak, J. W. Concentration-Driven Growth of Model Protocell Membranes. *J. Am. Chem. Soc.* **2012**, *134*, 20812−20819.

(S8) Blanco, M.; Lunardon, M.; Bortoli, M.; Mosconi, D.; Girardi, L.; Orian, L.; Agnoli, S.; Granozzi, G. Tuning on and off chemical- and photo-activity of exfoliated $MoSe_2$ nanosheets through morphologically selective “soft” covalent functionalization with porphyrins. *J. Mater. Chem. A*, **2020**, *8*, 11019–11030.

(S9) Takakura, K.; Yamamoto, T.; Kurihara, K.; Toyota, T.; Ohnuma, K.; Sugawara, T. Spontaneous Transformation from Micelle to Vesicle Associated with Sequential Conversions of Comprising Amphiphiles within Assemblies. *Chem. Commun.* **2014**, *50*, 2190–2192.

(S10) Nagarajan, R. Molecular Packing Parameter and Surfactant Self-Assembly: The Neglected Role of the Surfactant Tail. *Langmuir* **2002**, *18*, 31–38.
